# A viral ORFeome library for systems-level genetic dissection of host-pathogen interactions

**DOI:** 10.1016/j.cell.2026.05.024

**Published:** 2026-07-03

**Authors:** Eric Fujimura, Colin N. O’Leary, Mamie Z. Li, Rachel A. Roberts, Caleb R. Glassman, Joao A. Paulo, Hanjie Jiang, Nouran S. Abdelfattah, Eric C. Wooten, Zachary Mirman, J. Wade Harper, Philip A. Cole, Stephen J. Elledge

**Affiliations:** 1 Department of Genetics, Harvard Medical School, Boston, MA 02115, USA; 2 Division of Genetics, Department of Medicine, Brigham and Women’s Hospital, Boston, MA 02115, USA; 3 Program in Chemistry and Chemical Biology, Harvard University, Cambridge, MA 02115, USA; 4 Program in Virology, Harvard Medical School, Boston, MA 02115, USA; 5 Department of Cell Biology, Harvard Medical School, Boston, MA 02115, USA; 6 Department of Biological Chemistry and Molecular Pharmacology, Harvard Medical School, Boston, MA 02115, USA; 7 Program in Biological and Biomedical Sciences, Harvard Medical School, Boston, MA 02115, USA; 8 Howard Hughes Medical Institute, Boston, MA 02115, USA; 9 These authors contributed equally; 10 Lead contact

## Abstract

Virological research has traditionally focused on individual viruses or viral families. Advances in DNA synthesis now allow large-scale construction of individual gene products, enabling systematic exploration of the virome. Here, we developed a barcoded library of ~12,000 viral open reading frames (vORFs) from 513 viral species, which we leveraged to identify hundreds of viral regulators of cellular proliferation, MHC class I antigen presentation, and interferon signaling. Integrating results across these screens revealed unique phenotypic profiles and functional vORF modules, allowing the in-depth characterization of two previously uncharacterized viral proteins, MC162R and Yaba-like disease virus (YLDV) 151R, which impair MHC class I antigen presentation and interferon (IFN)-β signaling, respectively. Together, the viral ORFeome provides a scalable framework for dissecting viral protein function across the breadth of the virome.

## In brief

A scalable library of ~13,000 barcoded viral open reading frames from >500 viral species enables functional genomic screens across the virome. Applying this platform identified viral regulators of cellular proliferation, antigen presentation, and interferon signaling. This platform enables systematic exploration of viral protein function.

## Graphical Abstract

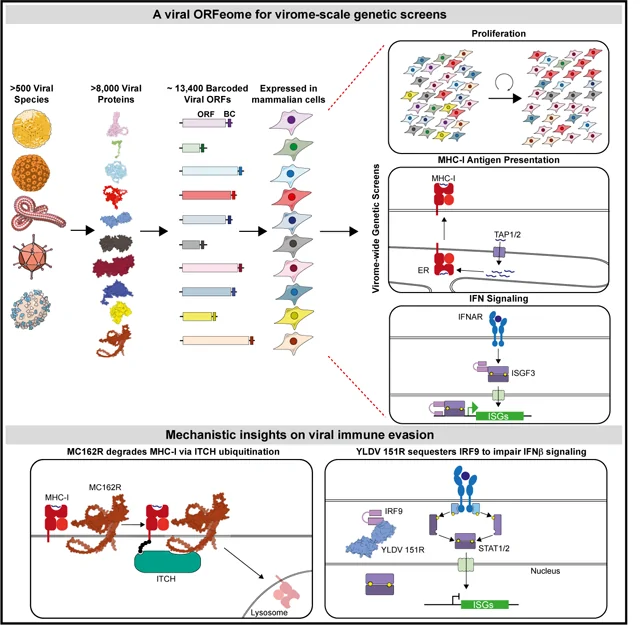

## INTRODUCTION

Viral pathogens pose a significant global threat, as evidenced by multiple pandemics that have engulfed humanity in the last century alone.^1–3^ Modern connectivity enables a single zoonotic transmission to rapidly spread at a time when zoonotic transmission to humans is increasing because of changing human interactions with nature.^4^ While zoonotic viruses that spill over into humans are often related to known human pathogens, there is a rich diversity of animal viruses, some of which differ significantly from their human homologs and, in principle, can evolve to transmit to and between humans.

The human virome spans 26 viral families, of which 20 are known to cause disease in humans, with diseases ranging from acute illness to chronic infection.^1^ Virology research tends to focus on priority pathogens or representative ‘‘prototype’’ or model pathogens, with individual labs often studying a single virus or viral family. These approaches leave many viral pathogens, including emerging viruses of potential concern, and their component proteins uncharacterized. Understanding the function of these viral components is essential for identifying viruses that have evolved sufficient molecular compatibility with potential hosts to enable cellular entry and viral replication, and for developing countermeasures against them. A critical component of this molecular compatibility is the ability to inhibit the host’s innate and adaptive immune responses.

Previous efforts to perform genetic screens of viral open reading frames (vORFs) primarily used ORF libraries from individual viruses/viral families, or ORF collections computationally predicted to exhibit a specific phenotype.^2–6^ While useful, these approaches leave much of the human pathogenic virome unprobed, can be cost-prohibitive, and, in most cases, do not allow for comparison of evolutionarily related viral ORFs. Advances in gene synthesis technology, however, enable the construction of large-scale libraries of synthesized genes.

Here, we develop a library of vORFs that captures the diversity of the known human and animal virome to enable systematic analysis of viral protein function. We leveraged this viral ORFeome to identify previously uncharacterized regulators of diverse cellular processes, including >700 viral regulators of cellular proliferation, MHC class I antigen presentation, and interferon (IFN) signaling. Characterization of specific regulators identified a molluscum contagiosum virus (MCV) protein, MC162R, that recruits multiple host E3 ligases to degrade MHC class I, and a selective inhibitor of IFN-β signaling, Yaba-like disease virus (YLDV) 151R, that is the first poxvirus protein characterized to inhibit IRF9 functionality. Our results reveal that numerous viral proteins that regulate mammalian cellular processes and may contribute to pathogenicity remain uncharacterized. Moreover, by probing across the evolutionary space of the virome, we identify related viral proteins that have divergent phenotypes, underscoring the constant evolution of viral proteins. Understanding the function of these viral proteins may yield further insight into the mechanisms by which viruses manipulate host cellular processes. Future viral ORFeome screening will enable the large-scale discovery of viral proteins that impact a multitude of cellular processes and set the stage for a systems biology effort to characterize viral protein function across the virome, shedding light on known human pathogens and emerging viruses with zoonotic potential.

## RESULTS

### Development of a viral ORFeome

**Figure 1.**
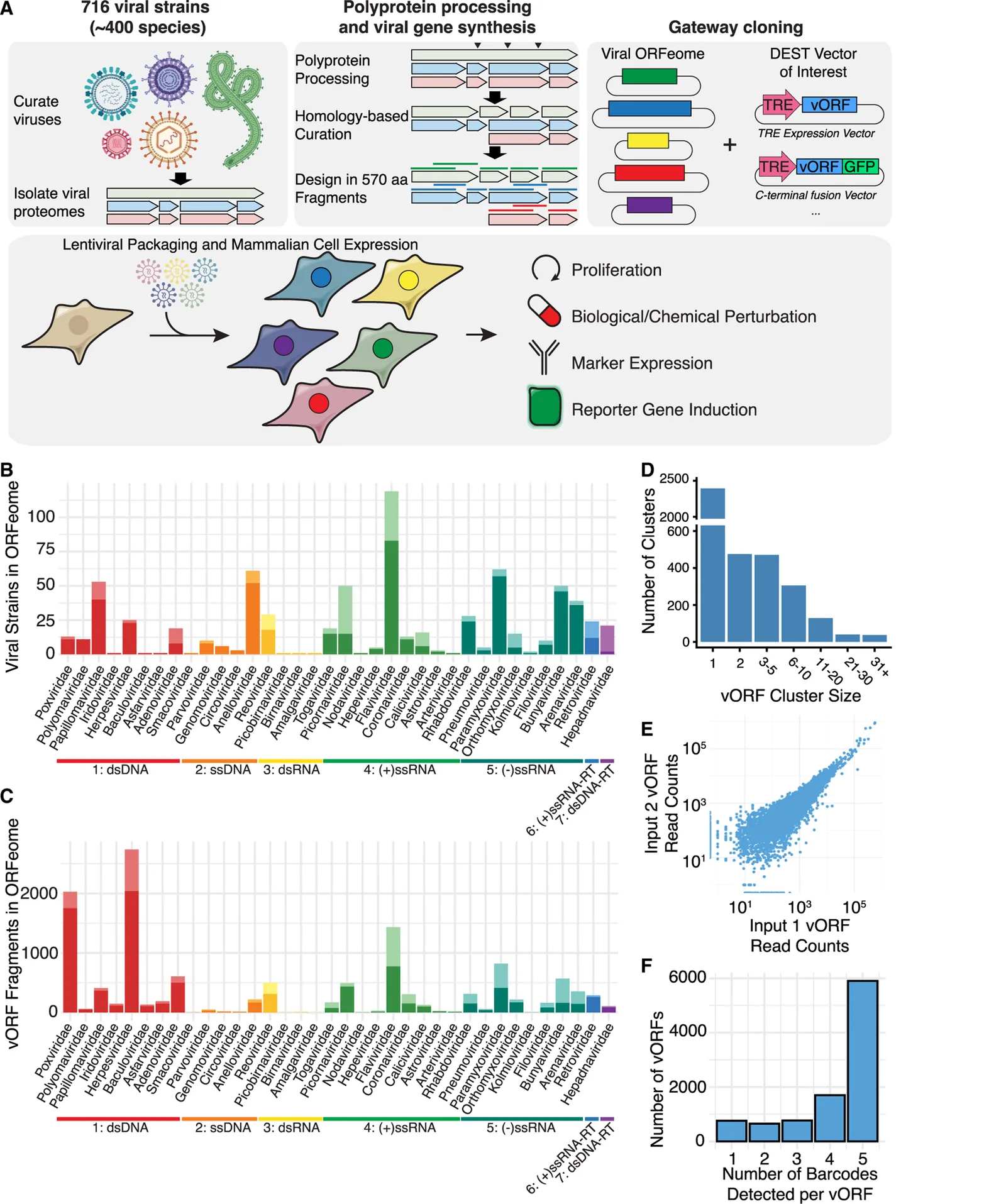
Development of a viral ORFeome (A) Schematic of the design and utility of the viral ORFeome. (B) Distribution of viral species in the viral ORFeome collection by viral family (with non-arenavirus Bunyaviridae ORFs consolidated). Within each family, dark coloring indicates the total number of unique viral species, and light coloring indicates the number of additional strains of viruses present in the viral ORFeome. (C) Distribution of vORF constructs in the viral ORFeome collection by viral family (with non-arenavirus Bunyaviridae ORFs consolidated). Within each family, dark coloring indicates the total number of unique UniProt identifiers from each viral family represented in the viral ORFeome, while the light coloring indicates the additional vORF constructs resulting from the tiling of UniProt entries that were greater than 570 amino acids in length. (D) Representation of distribution of vORF sequence homology-based clusters by cluster size. (E) The total read counts associated with each vORF detected following barcode amplification from genomic DNA from two populations of A375 cells independently transduced with the viral ORFeome following 48 h of doxycycline induction. (F) The number of barcodes per vORF detected following barcode amplification from A375 cells transduced with the viral ORFeome following 48 h of doxycycline induction. See also Figure S1 and Table S1.

We designed a viral ORFeome collection containing the sequences of individual proteins or genome polyproteins identified from 513 viral species with human tropism or animal viruses closely related to human viruses in the UniProt database (Figures 1A and 1B).^7^ This collection was supplemented with several additional strains of viruses from antigenically diverse viral families, such as influenza A viruses (IAVs), and a complete collection of human cytomegalovirus (CMV) ORFs that were identified by ribosome profiling.^8^ For genome polyproteins, we separated out post-cleavage protein products where annotated; otherwise, the polyprotein was synthesized as fragments. As our DNA synthesis length was limited to 1,710 base pairs (570 amino acids), larger proteins were divided into 570 amino acid fragments, with 285 amino acid overlaps between the fragments (Figure S1A). Conversely, small vORFs were synthesized as concatemers and deconvoluted via restriction cloning (Figures S1A and S1C). In this manner, we synthesized constructs encoding 11,821 vORFs (and 777 non-viral ORFs derived from the Immune Epitope Database),^9^ of which ~10,000 were deconvoluted and pooled for screening (Figures 1B and 1C; Table S1). These vORFs formed 1,458 clusters of >2 vORFs with at least 25% sequence identity, of which 18 included vORFs from multiple viral families, plus 2,377 singletons (Figures 1C and S1F).

Each vORF fragment was synthesized with a unique barcode for genetic screening and a removable stop codon and was cloned into the pFuji101 recipient vector, a vector which supports (1) gateway recombination of the complete viral ORFeome, (2) T7-driven *in vitro* transcription/translation and PLATO-based ribosome display, and (3) flexible transfer of vORFs between vectors using meganuclease sites (Figure S1B).^10,11^ Individual ORF barcodes were diversified (up to five per vORF in this study) by recoding the final 10 nucleotides of the barcode region via restriction digestion (Figures S1D and S1E). We introduced the vORF library into a TRE-inducible mammalian expression vector and lentivirally transduced it into A375 cells expressing the reverse tet transactivator (rtTA). Amplification and next-generation sequencing of the barcodes from the cellular genomic DNA revealed that we could readily detect over 9,000 vORFs associated with over 43,000 unique barcodes following two unique transductions of the vORF library, with the median ORF having 5 unique barcodes attributed to it (Figures 1D, 1F, and S1G).

### Identification of viral ORFs that regulate cellular proliferation

Viral proteins hijack cellular functions, steering them to generate an optimal environment for viral propagation, often at the expense of normal cellular homeostasis. Thus, we sought to characterize the utility of the viral ORFeome for genetic screens by screening vORFs for regulators of cellular proliferation, a phenotype that integrates many cellular pathways. We introduced and induced expression of the viral ORFeome in retinal pigment epithelium (RPE1) cells, human colonic epithelial cells (HCECs), and the human melanoma cell line A375, which expressed rtTA, and passaged them for 5–7 population doublings. Afterward, cells were collected, barcodes were amplified, and the samples were sequenced. Barcode abundance was analyzed with MAGeCK to determine the enrichment or depletion of specific vORFs within the population.^12^

**Figure 2.**
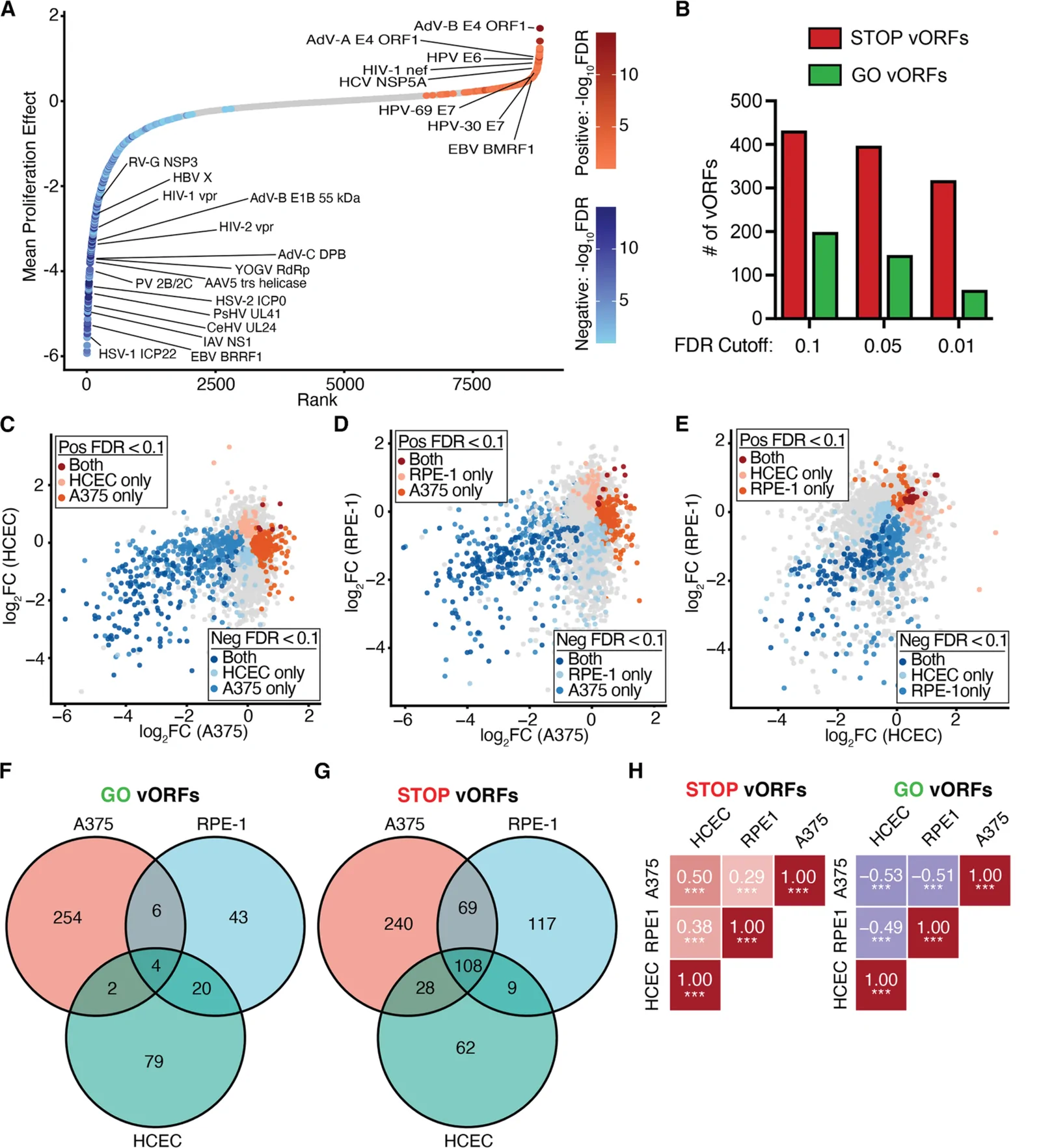
Identification of vORFs that regulate cellular proliferation (A) Meta-analysis of the proliferation screens in A375, HCEC, and RPE1 cells identifying vORFs with shared phenotypes across the three screens. (B) The number of vORFs achieving significance at the indicated false discovery rate (FDR) cutoff values in the combined analysis of the three screens. (C–E) Pairwise comparisons of the log_2_fold changes for vORFs from the (C) A375 and HCEC screens, (D) A375 and RPE1 screens, and (E) HCEC and RPE1 screens. Individual vORFs are colored by whether they achieved significance in the screen and whether they were enriched or depleted. (F) A Venn diagram representation of the overlap of vORFs detected in all three proliferation screens that increased cellular proliferation (positive FDR < 0.1, as calculated via MAGeCK). (G) A Venn diagram representation of the overlap of vORFs detected in all three proliferation screens that decreased cellular proliferation (negative FDR < 0.1, as calculated via MAGeCK). (H) Pairwise Pearson correlations of the STOP or GO vORFs that achieved significance in each of the three proliferation screens. See also Figure S2 and Table S2.

We assessed the performance of the vORFs across all three screens and found that twice as many vORFs consistently reduced (‘‘STOP vORFs’’) versus increased (‘‘GO vORFs’’) cellular proliferation at each significance cutoff examined (Figures 2A and 2B; Table S2). This observation was repeated within each of the individual cell line proliferation screens (Figures S2A–S2F; Table S2). The tendency of vORFs to impair cellular proliferation likely reflects the fact that many viral proteins are involved in reprogramming membrane, metabolic, transcription, and replication dynamics, resulting in ER stress responses, DNA damage, oxidative stress, induction of apoptosis, and other deleterious responses that likely impair proliferation and survival.^13,14^ We identified several homologous vORFs that perform similarly in the screens (Figures S2G and S2H).^15–20^

Comparison of individual screens revealed that, for each cell line, at least 44% of STOP vORFs identified in each cell line were found in multiple cell lines (A375: 44%; HCEC: 70%; RPE-1: 61%), whereas for the GO vORFs, the number achieving significance in one cell line and shared with others was consistently lower (A375: 5%; HCEC: 25%; RPE-1: 58%) (Figures 2C–2G). This high level of GO vORF tissue specificity aligns with previous research on human ORFs that increase proliferation.^21^ This notion of cell-type specificity is reinforced by the finding that STOP vORFs are positively correlated across cell types, whereas GO vORFs are not (Figure 2H). Among the STOP vORFs identified in multiple screens were known toxic proteins, including herpesvirus UL41 host shutoff proteins derived from cercopithecine herpesvirus (CeHV), gallid herpesvirus (GaHV), psittacid herpesvirus (PsHV), and the rotavirus protein synthesis inhibitor NSP3, and many proteins not previously implicated in cellular proliferation.^17,22^ Likewise, the GO vORFs identified across multiple screens include well-established factors that promote cellular proliferation, such as human immunodeficiency virus (HIV) nef, adenovirus E4-ORF1, and human papillomavirus (HPV) E7.^19,20,23^

### A genetic screen identifies vORFs that regulate MHC class I antigen presentation

**Figure 3.**
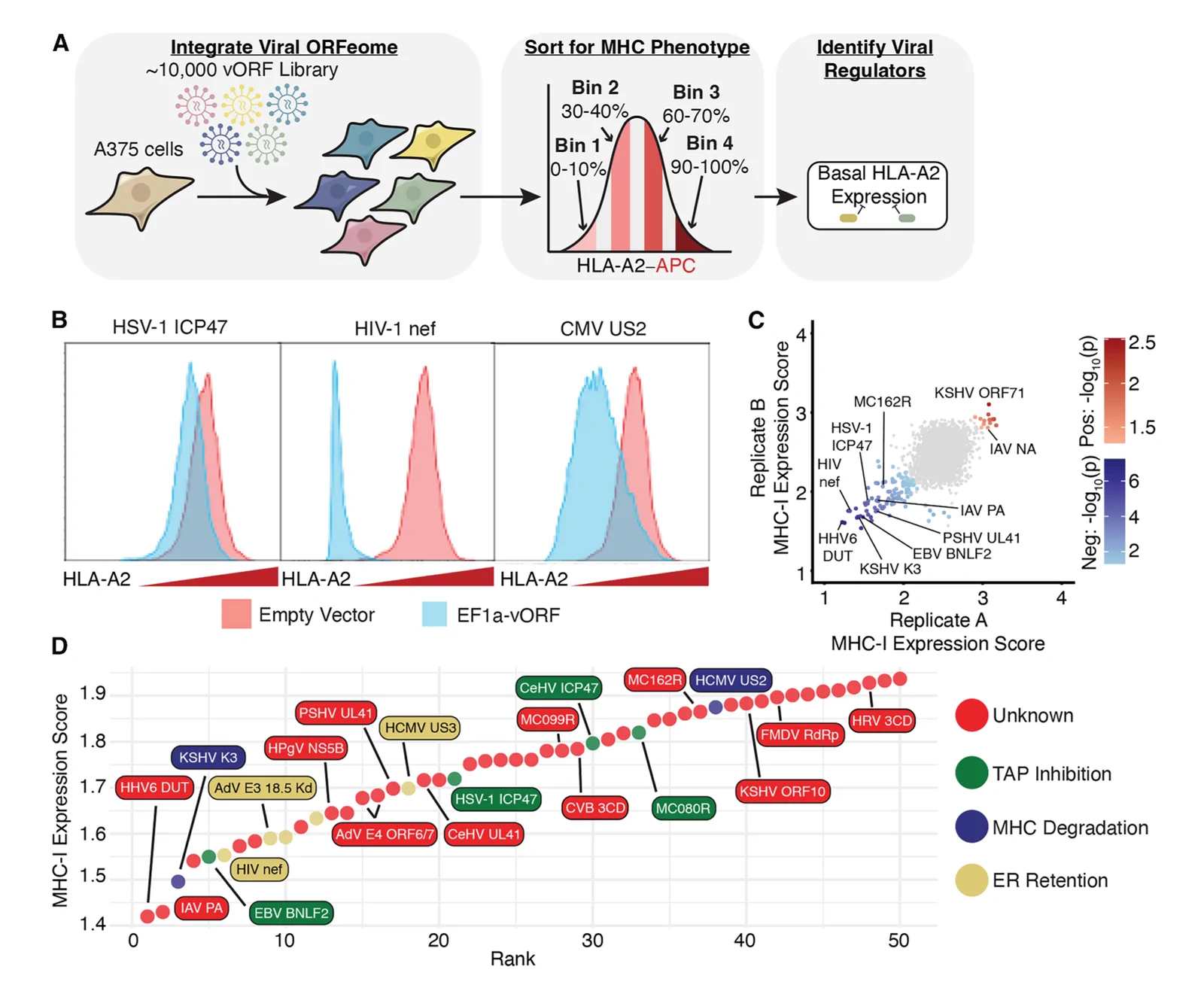
A genetic screen identifies vORFs that regulate MHC class I antigen presentation (A) The design of the screen for vORFs that regulate HLA-A2 cell surface expression. (B) HLA-A2 cell surface staining as measured by flow cytometry in A375 cells constitutively expressing the indicated vORF. (C) The HLA-A2 expression scores for all vORFs identified across two replicates. (D) A histogram representation of the top 50 vORFs that reduce surface HLA-A2 staining identified in the screen, ranked by their MHC class I expression score. Each vORF is colored based on its characterized role in regulating MHC class I expression, or colored red if not previously implicated. See also Figure S3 and Table S3.

We next sought to probe how vORFs enable immune evasion through regulation of MHC class I antigen presentation, which is critical for CD8 T cell-mediated killing of virally infected cells (Figure 3A).^24–26^ A375 cells express the MHC class I molecule HLA-A2, which is readily detectable via cell surface staining (Figure S3A) and downregulated by several known viral proteins known to reduce cell surface MHC class I (Figure 3B).^27–30^ To identify MHC class I regulators, we expressed the viral ORFeome in A375 cells and performed a fluorescence-activated cell sorting (FACS)-based screen for HLA-A2 expression (Figure 3A). We devised an MHC class I expression score for each vORF to rank the vORFs with respect to the cell surface HLA-A2 expression level based on the abundance of vORF barcodes across four bins, with a lower MHC-I expression score indicating less cell surface MHC class I on cells expressing a given vORF (Figures 3A–3C and S3B).

The screen identified several viral proteins known to antagonize MHC class I antigen presentation, including HIV nef and Kaposi sarcoma-associated herpesvirus (KSHV) K3, as well as many candidate vORFs that have not been previously implicated in MHC class I downregulation (Figure 3D; Table S3).^31^ We mapped the vORFs that were significant in this screen onto sequence-based clusters and identified several related vORFs that reduced HLA-A2 cell surface expression, including both known and previously uncharacterized antagonists (Figure S3C).^32^ Interestingly, the screen identified several vORFs that increased HLA-A2 surface staining, including two IAV neuraminidase vORFs (Figure S3C; Table S3).

### MC162R is an antagonist of antigen presentation via MHC class I lysosomal degradation

**Figure 4.**
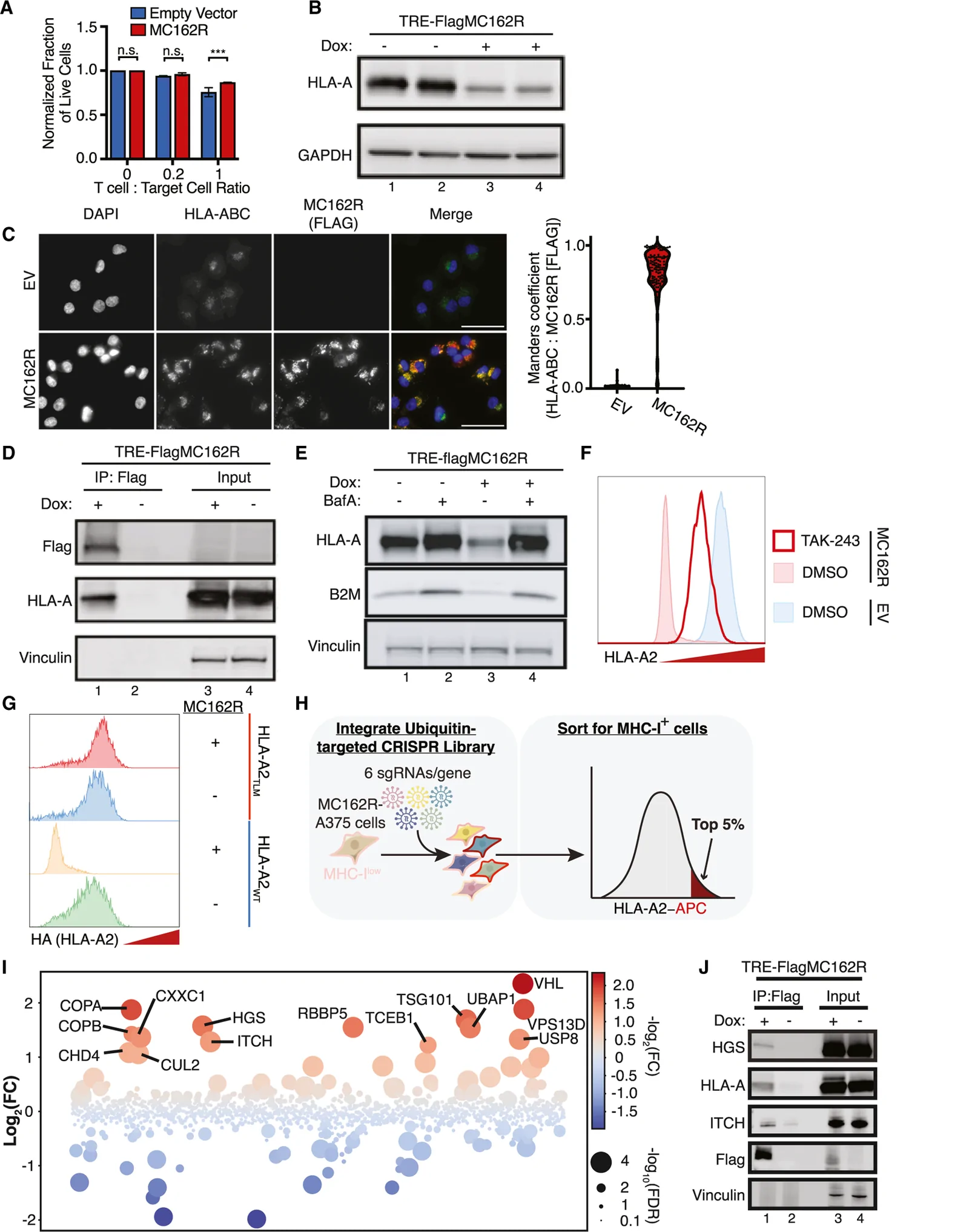
MC162R antagonizes MHC class I antigen presentation via MHC class I lysosomal degradation (A) The fraction of live CellTrace Violet^+^ A375 cells expressing MC162R or an empty vector control following a 12 h incubation at the indicated ratio with CFSE^+^ CD8 T cells recognizing the NY-ESO-1 epitope as determined by flow cytometry (*n* = 3 biological replicates). Data are represented as mean ± SD. (B) Immunoblot for HLA-A in A375 cells expressing doxycycline-inducible FLAG-MC162R in the presence or absence of doxycycline. (C) (Left) Fluorescence microscopy for FLAG-MC162R and HLA-ABC in A375 cells expressing FLAG-MC162R (*n* = 110 cells) or an empty vector control (*n* = 128 cells) following 36 h doxycycline induction and 12 h bafilomycin A treatment. Scale bar, 50 μm. (Right) Quantification of the colocalization between MC162R (FLAG) and HLA-ABC via Manders coefficient. (D) Immunoblot for FLAG-MC162R and HLA-A following FLAG immunoprecipitation in FLAG-MC162R or empty vector expressing A375 cells. (E) Immunoblot for HLA-A expression in A375 cells expressing doxycycline-inducible FLAG-MC162R or an empty vector control following treatment with bafilomycin A1. (F) Expression of cell surface HLA-A2 in A375 cells expressing doxycycline-induced FLAG-MC162R or an empty vector control following treatment with TAK-243. (G) Expression of either a WT HA-tagged HLA-A2 or an HA-tagged HLA-A2 containing three lysine to alanine mutations (HLA-A2_TLM_) in the cytoplasmic domain of the protein corresponding to potential ubiquitination sites, as determined by anti-HA staining and flow cytometry analysis. (H) The design of a CRISPR screen for host genes required for MC162R-mediated downregulation of HLA-A2 surface expression. (I) Results of genome-wide CRISPR screens for host factors that regulate MC162R-mediated downregulation of HLA-A2 surface expression analyzed via MAGeCK. (J) Immunoblot for HLA-A, ITCH, and HGS following anti-FLAG immunoprecipitation from tetracycline-inducible FLAG-MC162R expressing cells. Statistical comparisons were performed using a two-way ANOVA with Sidak’s post hoc test *** *adj p* < 0.001, not significant (n.s.) (A). See also Figure S4 and Table S4.

We selected an uncharacterized protein, MC162R, from MCV for in-depth characterization. MCV is the causative agent of molluscum contagiosum cutaneous infection associated with benign skin neoplasms common in childhood.^33^ We validated that expression of MC162R reduced surface HLA-A2 staining (Figure S4A) and that this phenotype extended across HLA-A, -B, and -C alleles, suggesting that MC162R broadly reduces surface MHC class I expression (Figures S4B and S4C). MC162R expression did not impact the cell surface expression of unrelated cell surface molecules, such as HER2, ERBB3, and CD54, demonstrating its selectivity (Figure S4D).^34^ Functionally, MC162R expression partially protected A375 cells from T cell killing when incubated with T cells recognizing the NY-ESO-1 antigen (Figure 4A).^35^

Not only was HLA-A removed from the surface, but its total protein levels also decreased in MC162R-expressing cells (Figure 4B), whereas the levels of HLA-A transcripts were unchanged (Figure S4E), suggesting that MC162R facilitated the degradation of HLA-A. Colocalization between MHC class I and MC162R was observed in all cells with detectable MC162R expression, suggesting proximity within cells (Figure 4C). Immunoprecipitation of MC162R pulled down HLA-A, suggesting a possible direct interaction (Figure 4D). We hypothesized that MC162R facilitated the degradation of MHC class I via the lysosomal pathway, which typically degrades membrane proteins. Treatment with the lysosome inhibitor bafilomycin A1 attenuated MC162R-mediated degradation of MHC class I (Figures 4E and S4F) and increased MHC class I accumulation in LAMP1^+^ late endosomal and lysosomal compartments, confirming degradation via this pathway (Figure S4G).

During our examination of inhibitors of protein degradation pathways, we found that treatment of MC162R-expressing cells with an E1 inhibitor, TAK-243, or a 26S proteasome inhibitor, MG-132 (which also depletes the cellular ubiquitin pool), partially restored HLA-A2 surface staining, suggesting that MC162R is reliant on the cellular ubiquitination machinery to target MHC class I for surface removal and destruction (Figures 4F and S4H).^36,37^ We then sought to determine whether MC162R facilitates the ubiquitination of MHC class I. MHC class I molecules contain three lysine residues in their cytoplasmic domain that could be ubiquitylated by E3 ligases to target them for lysosomal degradation.^31,38^ To assess their role in MC162R regulation of MHC class I levels, we generated an HLA-A2 triple lysine mutant (TLM) replacing all three lysines in the cytoplasmic domain with alanines. Cell surface expression of HLA-A2_TLM_ was not impacted by MC162R expression, implying that MC162R facilitated the ubiquitination of MHC class I on its cytoplasmic domain to promote its degradation (Figure 4G).

### MC162R exploits multiple E3 ligases to degrade MHC class I

MC162R does not contain domains that directly facilitate MHC class I ubiquitination, suggesting that it may co-opt host E3 ligases to degrade MHC class I.^39^ To identify host genes involved in MC162R function, we performed a FACS-based CRISPR suppression screen for increased HLA-A2 expression using a ubiquitin-related library in MC162R-expressing cells (Figures 4H and S4I).^40^ This screen identified several genes that restored HLA-A2 expression in MC162R-expressing cells, including two ubiquitin E3 ligases, the CUL2^VHL^ E3 ligase complex and NEDD4-like E3 ligase ITCH, as well as components of the endosomal sorting complex required for transport (ESCRT) machinery, such as HGS (also known as HRS) and TSG101, which recognize ubiquitylated cargo and sort it into multivesicular bodies, respectively (Figure 4I; Table S4).^41–43^ NEDD4 family E3 ligases are known to regulate cell surface receptor degradation via ubiquitination and lysosomal degradation, though none have been implicated in MHC class I regulation.^44^ The ESCRT machinery facilitates the transport of ubiquitylated cell surface proteins through the endosome to the lysosome for degradation, consistent with our findings that MC162R promotes lysosomal degradation of MHC class I.^43^

Using small interfering RNA (siRNA) depletion and CRISPR-mediated mutation, we validated the suppressor screen in cells expressing MC162R and found that siRNA depletion (Figure S4J) caused mild restoration of MHC class I on the cell surface, whereas gRNA-Cas9-mediated mutation (Figure S4K) showed stronger effects. Mutations in COPA, HGS, ITCH, TSG101, UBAP1, and VPS13D increased HLA-A2 surface staining, although none fully restored cell surface expression. Notably, perturbing genes involved in intracellular trafficking (VPS13D, HGS, and COPA) led to more pronounced increases in HLA-A2 surface staining than disrupting individual E3 ligases, such as ITCH. Additionally, we successfully coimmunoprecipitated MC162R with both ITCH and HGS, providing further evidence for their involvement in MC162R’s function (Figure 4J).

**Figure 5.**
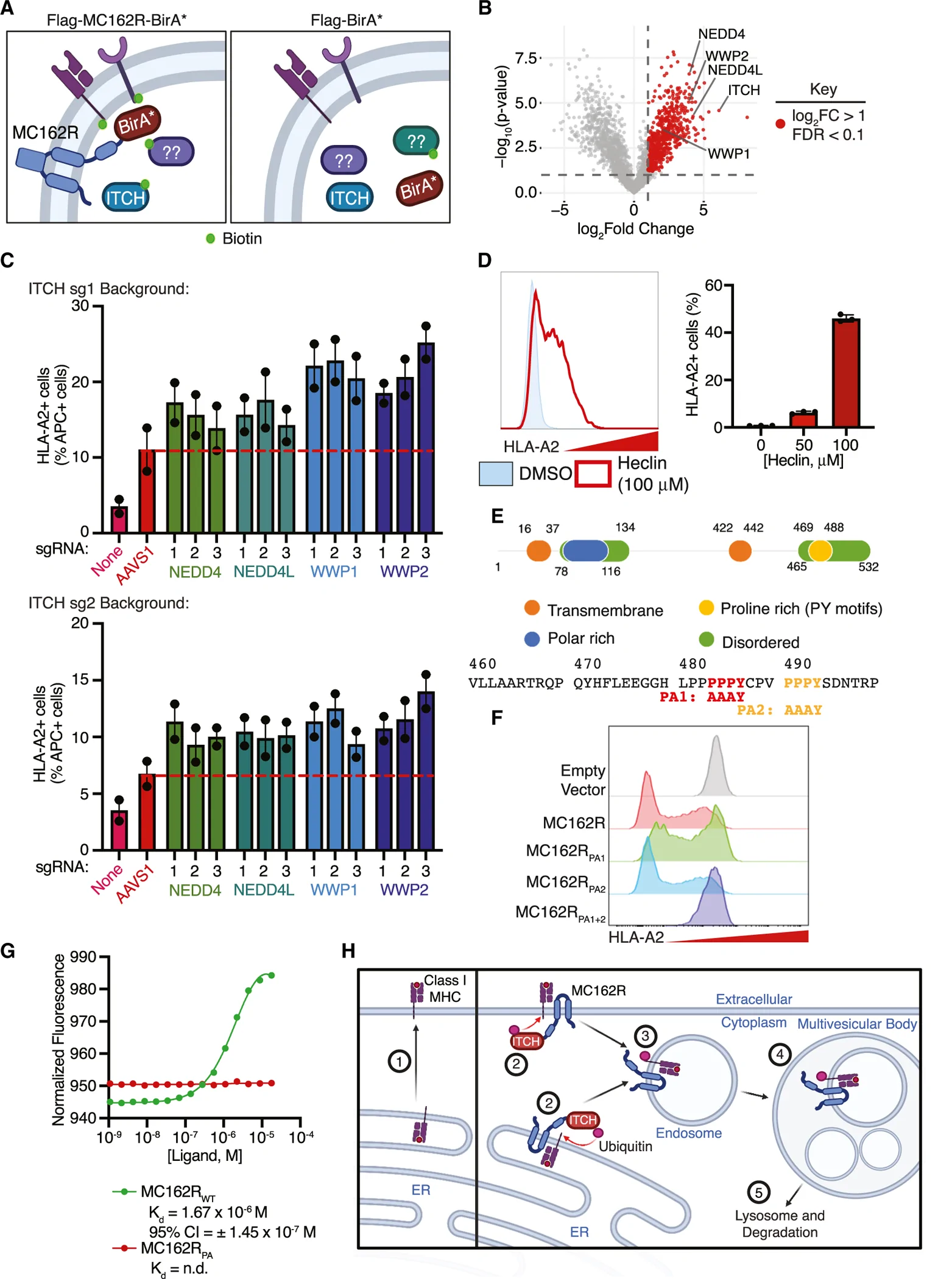
MC162R exploits multiple E3 ligases to degrade MHC class I (A) A schematic depiction of the BirA*-FLAG-MC162R BioID experiment design. This image was generated with BioRender. (B) Volcano plot for proteins that were enriched or depleted following streptavidin pull-down in BirA*-FLAG-MC162R expressing cells compared with BirA*-FLAG expression cells. Selected proteins are indicated. (C) Quantification of the fraction of HLA-A2^+^ A375 cells detected by flow cytometry harboring the indicated sgRNA targeting a NEDD4-like E3 ligase in cells expressing an sgRNA targeting ITCH. ‘‘None’’ refers to cells expressing MC162R, but not an sgRNA or Cas9. Data are replicates assayed in duplicate and depicted as mean ± range with individual values plotted. Data are representative of multiple independent experiments. (D) Representative flow cytometry histograms of the expression of cell surface HLA-A2 in A375 cells expressing tetracycline-inducible MC162R and treated with heclin (left) and quantification of HLA-A2 surface staining in A375 cells expressing MC162R and treated with heclin at the indicated concentration (right). Data are technical replicates assayed in triplicate and depicted as mean ± standard deviation with individual points plotted. Data are representative of multiple independent experiments. (E) A schematic depiction of the structure of MC162R (top) and the sequence of the region surrounding the two identified PY motifs in the MC162R disordered region. The mutations generated in the PY motifs are designated as PA1 and PA2. (F) Cell surface HLA-A2 staining assessed by flow cytometry in A375 cells constitutively expressing the indicated MC162R construct or an empty vector control. See also Figure S5 and Table S5. (G) Microscale thermophoresis assay for the ability of peptides containing the WT MC162R PY motifs or PPPY > AAAA mutations to both PY motifs to bind to purified murine ITCH143–864. (H) Proposed mechanism of MC162R-mediated MHC class I downregulation. (1) Under basal conditions, class I HLA is loaded with peptides in the endoplasmic reticulum and traffics to the cell surface. (2) MC162R associates with class I HLA and recruits ITCH and other NEDD4-like ligases to ubiquitylate MHC class I. (3) MHC class I, in complex with MC162R, is internalized and trafficked via the ESCRT machinery into the endosomal sorting pathway. (4 and 5) Ubiquitylated MHC class I is sorted into multivesicular bodies and degraded via the lysosome. This image was generated with BioRender.

The partial restoration of HLA-A2 surface staining following ITCH mutation suggested that key components may have been missing. To identify other proteins that cooperate with MC162R to degrade MHC class I, we created an MC162R-BirA* fusion construct for proximity labeling, streptavidin pull-down, and mass spectrometry analysis (Figures 5A and S5A).^45^ MC162R-BirA expressing cells showed enriched biotinylation of numerous cell surface proteins and lysosomal-trafficking factors (Figure S5B). Importantly, MC162R-enriched biotinylated proteins included the five cytoplasmic NEDD4-like E3 ligase family members (Figures 5B and S5C; Table S5) but not nuclear family members, such as SMURF1 and SMURF2.^44^

The identification of multiple associated NEDD4-like E3 ligases suggested that MC162R may redundantly recruit multiple NEDD4-like E3 ligases to promote MHC class I degradation. Mutation of other NEDD4-like E3 ligase family members revealed that WWP1 and WWP2 loss modestly increased HLA-A2 surface staining relative to ITCH (Figure S5D). To test whether the mutation of multiple NEDD4-like ligases improved HLA-A2 cell surface expression, we generated mutations of NEDD4, NEDD4L, WWP1, and WWP2 in ITCH mutant cells. In all cases, mutation of an additional E3 ligase increased HLA-A2 surface staining compared with ITCH mutation alone (Figure 5C). We then used a pan-HECT ligase inhibitor heclin, which inhibits multiple HECT-domain-containing E3 ligases, and observed inhibition of MC162R-mediated MHC-I downregulation (Figure 5D).^46^ Moreover, heclin treatment of cells deficient in a NEDD4-like E3 ligase further increased surface HLA-A2 (Figure S5E). These findings support a model in which MC162R redundantly recruits NEDD4-like ligases to degrade MHC class I, in turn requiring multiple NEDD4-like E3 ligases to be inhibited to revert the phenotype.

NEDD4-like E3 ligases contain WW-domains that recruit substrates through an association with substrate polyproline motifs. We identified two polyproline motifs on MC162R and generated mutants of one or both (Figure 5E). The wild-type (WT), but not the double-mutant, MC162R polyproline motif was sufficient to bind ITCH *in vitro* (Figure 5G). Consistent with this, expression of MC162R with both polyproline motifs mutated completely ablated MC162R-induced loss of HLA-A2 cell surface expression (Figure 5F). This suggests that MC162R recruits NEDD4-like E3 ligases through its polyproline motifs to degrade MHC class I. Thus, we propose a model in which MC162R recruits NEDD4 family ligases to ubiquitinate and redirect MHC class I from the cell surface into the lysosomal degradation pathway (Figure 5H), thereby evading T cell killing.

The large number of biotinylated proteins found in MC162R-BirA cells indicated potential additional MC162R substrates. Whole-cell proteomics (WCP) revealed downregulation of many of these proteins in MC162R-expressing cells (Figure S5F; Table S6). We compared both the WCP and proximity biotinylated protein sets and examined the behavior of the MC162R biotinylated proteins as we applied increasingly stringent cutoffs for proteins depleted in the WCP. We observed progressive enrichment of proximity-labeled proteins as a fraction of those reduced in abundance as the significance threshold increased, suggesting that several proteins depleted by MC162R expression may be directly regulated (Figure S5G). While some may be direct MC162R substrates, others may be indirectly depleted via MHC class I colocalization and internalization. Notably, among the most strongly reduced proteins are known MHC class I interactors, such as a calreticulin (CALR) isoform, which may be lost through MHC class I association.^47^ However, given CALR’s role as an ‘‘eat-me’’ signal for macrophages and dendritic cells, its loss alone could benefit viral propagation.^48^ Overall, MC162R appears to broadly remodel cellular protein composition beyond impairing MHC class I antigen presentation.

### Identification of vORFs that regulate IFN signaling

**Figure 6.**
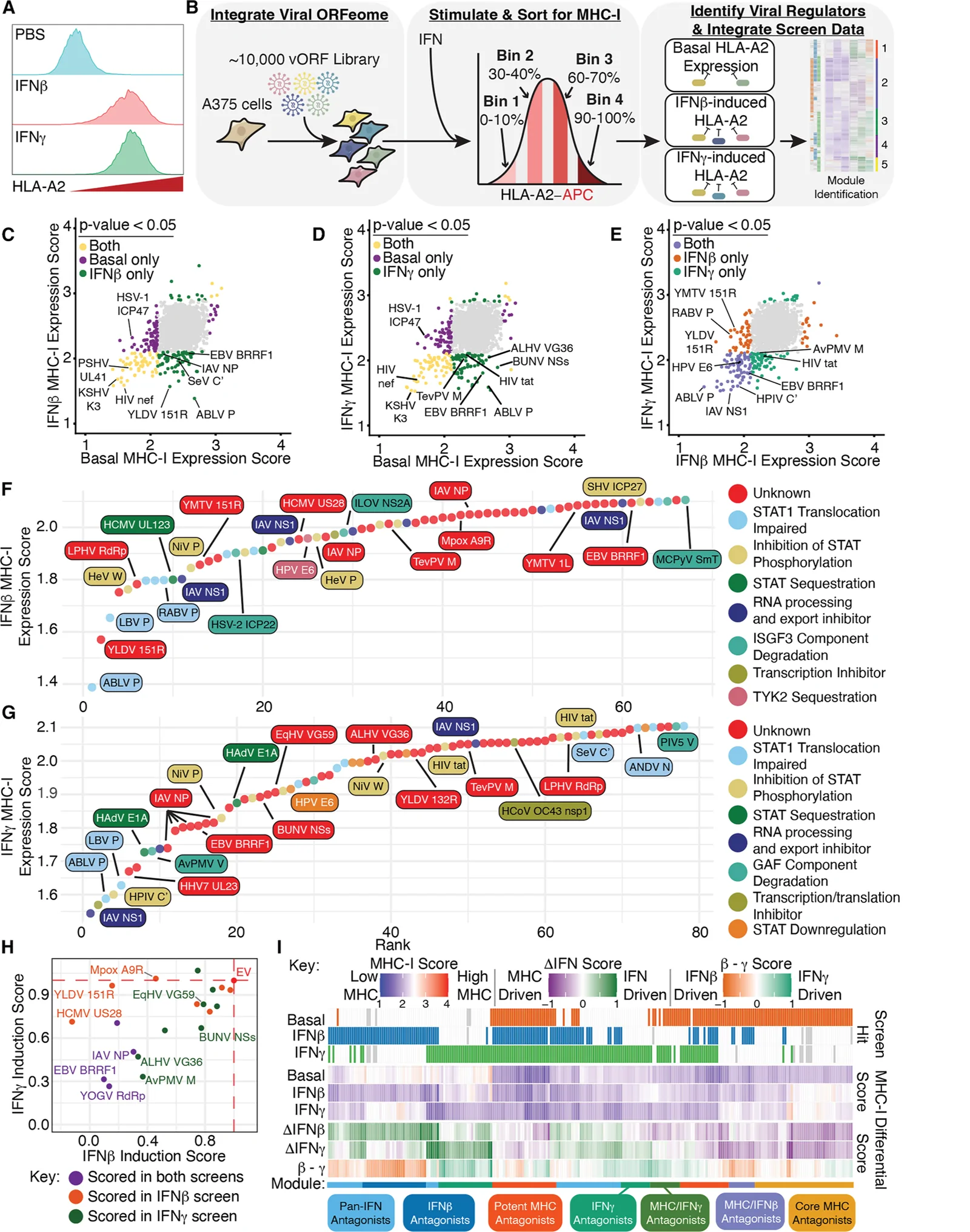
Identification of viral ORFs that regulate interferon signaling (A) Expression of cell surface HLA-A2 in A375 cells following treatment with PBS, IFN-β, or IFN-γ as determined by flow cytometry following staining with an APC-conjugated HLA-A2 primary antibody. (B) Schematic representation of the design of a screen for vORFs that regulate IFN-induced increases in surface HLA-A2 expression. (C–E) Pairwise comparisons of the (C) basal MHC class I expression screen and the IFN-β-induced MHC class I expression screen, (D) basal MHC class I expression screen and the IFN-γ-induced MHC class I expression screen, or (E) A pairwise comparison of the IFN-β-induced MHC class I expression screen and the IFN-γ-induced MHC class I expression screen. Individual vORFs are colored by the screen in which they achieved significance. (F) A schematic of the vORFs that achieved significance in the IFN-β-induced HLA-A2 expression screen (excluding vORFs that achieved significance in the basal HLA-A2 expression screen), ranked by their IFN-β-induced HLA expression score. For known regulators of IFN-β signaling, the vORF’s activity is indicated. (G) A schematic of the vORFs that achieved significance in the IFN-γ-induced HLA-A2 expression screen (excluding vORFs that achieved significance in the basal HLA-A2 expression screen), ranked by their IFN-γ-induced HLA expression score. For known regulators of IFN-γ signaling, the activity of the vORF is indicated. (H) Validation of vORFs not known to regulate IFN signaling as IFN-β or IFN-γ antagonists by flow cytometry. Changes in HLA-A2 staining were determined by taking the log_2_ of the MFIs between cells treated with IFN/cells treated with PBS and normalized to the empty vector control. (I) A combined analysis of all vORFs achieving significance and reducing MHC class I expression in at least one of the basal, IFN-β-induced, or IFN-γ-induced MHC class I expression screens. vORFs were clustered based on MHC expression scores and differentials across the three screens. Each vORF was further annotated with the screen in which it achieved significance (Screen Hit, *p* < 0.05) and its relationship with the other screens, calculated as the pairwise difference in MHC class I expression score between each combination of screens. Named modules were manually defined based on MHC expression patterns and differential expression across the three screens. See also Figure S6 and Table S3.

Viruses subvert both adaptive and innate immunity to efficiently produce progeny virions. Type I and II IFNs are secreted cytokines of the innate and adaptive immune responses, respectively, that induce an antiviral state in cells to limit viral infection and spread via ISG induction.^43–45^ Therefore, we sought to identify vORFs that impair signaling by type I and type II IFNs. Conveniently, HLA-A2 is an ISG that is increased on the cell surface following IFN-β or IFN-γ treatment, with the effects of IFN-β abrogated by the expression of rabies virus (RABV) phosphoprotein (P) (Figures 6A and S6A). We thus assessed MHC class I cell surface expression to perform a FACS-based screen for viral antagonists of IFN-β and IFN-γ signaling (Figures S6B and S6C; Tables S4). To account for vORFs that affect the basal MHC class I expression level, we restricted our analysis to vORFs that did not achieve significance in the basal MHC class I screen (Figures 6C, 6D, and S6D).

Most vORFs identified in this screen were not previously known to antagonize IFN signaling (Figures 6F and 6G). Among the many known IFN-β or IFN-γ signaling antagonists were RABV P, Nipah virus W, Sendai virus C^′^, and herpes simplex virus 1 (HSV-1) ICP22, many of which were part of families in which multiple homologous vORFs achieved significance (Figures 6F, 6G, and S6E).^49–55^ We selected a subset of 19 vORFs that scored in either the IFN-β or IFN-γ screens and were not previously implicated in antagonizing IFN signaling and validated that 16 reduced IFN-β- or IFN-γ-mediated induction of HLA-A2 when tested individually (Figures 6H and S6F). Several vORFs were validated to impair *ISG15* mRNA expression in cells treated with IFN-β to varying degrees (Figure S6G).

To identify vORFs with unique phenotype combinations, we integrated all three vORF screens utilizing MHC class I as a marker and identified multiple distinct functional modules (Figure 6I). This analysis allowed us to identify vORFs that produce phenotypes that were not readily apparent from examining the screens individually. For example, we identified several vORFs that achieved significance in only one of the IFN-β or IFN-γ screens (Figure 6E) but clustered in the pan-IFN antagonist module, suggesting effects in both. Some of these vORFs, such as AvPMV M and ALHV VG36, inhibited both IFN-β and IFN-γ signaling (Figure 6H), despite only achieving significance in the IFN-γ screen. Identifying these vORF phenotypes shows the power of performing multiple screens and integrating the results. Likewise, examining expression and differential scores reveals distinct module placement of lyssavirus P vORFs: vORFs such as ABLV P cluster as potent pan-IFN inhibitors, whereas IRKV P and SHIBV P cluster in a less potent pan-IFN module, and GBLV P clusters in an IFN-β-specific module and differentially inhibit IFN-γ signaling when validated (Figure S6H). Moreover, in conducting parallel screens, we identified vORFs that preferentially regulate IFN-β signaling (poxvirus C4/C10-like ORFs) or IFN-γ signaling (HIV tat) (Figures 6E and S6E). We were particularly intrigued by the ability of poxvirus C4/C10-like ORFs to specifically inhibit IFN-β signaling, which appeared to be limited to ORFs from the *Yatapoxvirus* genus.

### *Yatapoxvirus* 151R/1L vORFs specifically regulate IFN-β signaling via an IRF9 interaction

**Figure 7.**
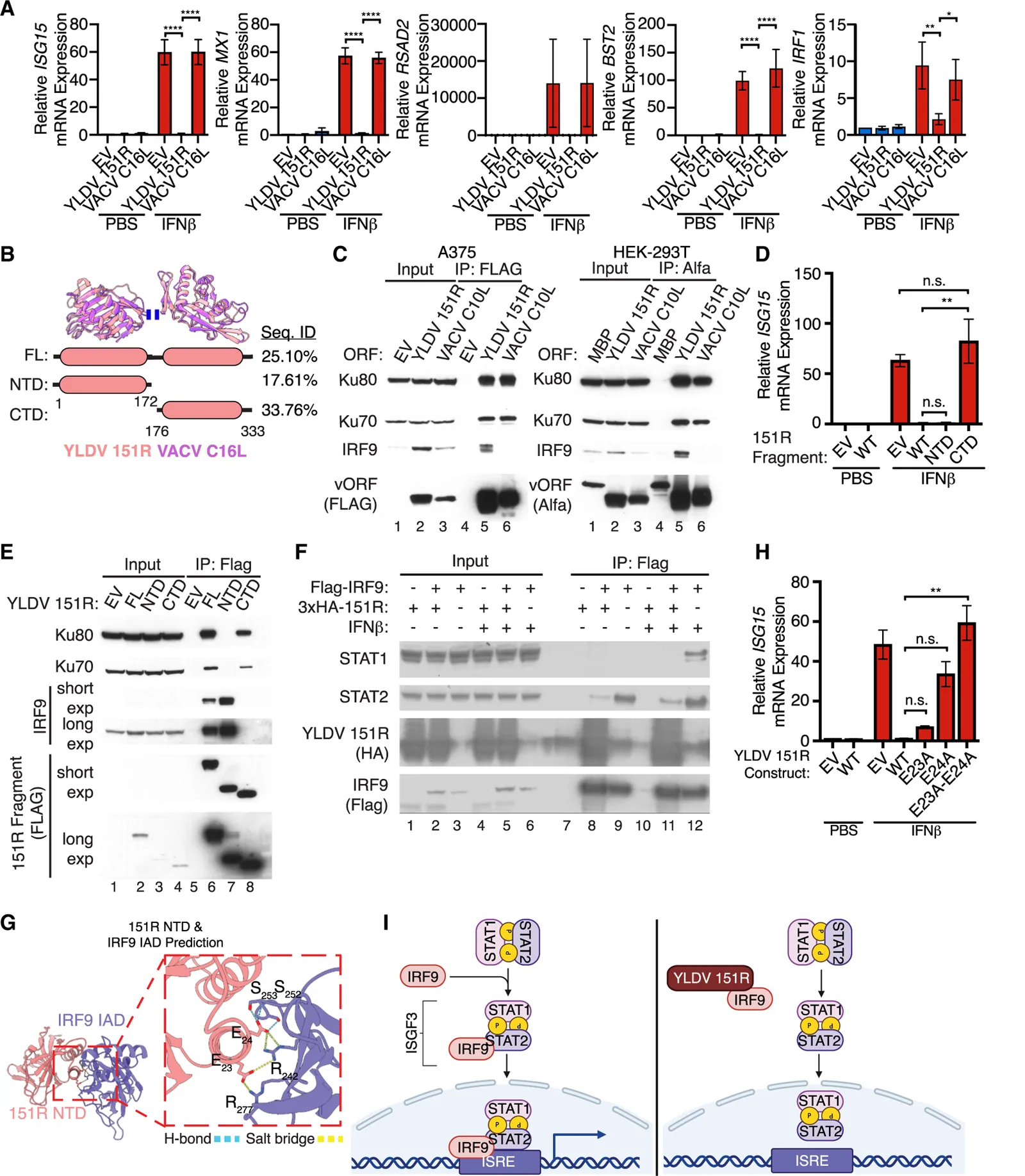
*Yatapoxvirus* 151R vORFs specifically regulate IFN-β signaling via an interaction with IRF9 (A) RT-qPCR for *ISG15*, *MX1*, *RSAD2*, *BST2*, and *IRF1* mRNA expression following IFN-β stimulus in A375 cells expressing an empty vector, YLDV 151R, or VACV C16L (*n* = 3 biological replicates). Data are represented as mean ± SD. (B) The alignment between an AlphaFold3 predicted structure of YLDV 151R and a previously described structure of VACV C16L (PDB: 8AG5/C). The NTDs and CTDs of YLDV 151R and VACV C16L were aligned individually. The linker region is depicted as a dashed line. A schematic of the domain structures used for deletion variants is below. (C) Immunoblot for IRF9, Ku70, Ku80, and FLAG-tagged vORFs following (left) FLAG immunoprecipitation from A375 cells or (right) ALFATag immunoprecipitation from HEK-293T cells of the indicated viral ORF. (D) RT-qPCR for *ISG15* mRNA expression following IFN-β stimulus in A375 cells expressing full-length YLDV 151R, 151R NTD, or 151R CTD (*n* = 3 biological replicates). Data are represented as mean ± SD. (E) Immunoblot for IRF9, Ku70, Ku80, and FLAG-tagged YLDV 151R deletion variants following immunoprecipitation of FLAG-tagged full-length YLDV 151R, YLDV 151R NTD, and YLDV 151R CTD from A375 cells. (F) Immunoblot for FLAG-IRF9, STAT1, STAT2, and 3xHA-YLDV 151R following immunoprecipitation of FLAG-IRF9 from A375 cells with and without IFN-β stimulation. (G) AlphaFold 3 structural prediction of the complex formed by the YLDV 151R NTD (pink) and IRF9 IAD (blue) (left). The predicted interacting residues within the predicted structure were identified by ePDB PISA. Interacting amino acid residues are indicated (right). (H) RT-qPCR for *ISG15* mRNA expression following IFN-β stimulus in A375 cells expressing YLDV 151R, YLDV 151R_E23A_, YLDV 151R_E24A_, or YLDV 151R_E23A-E24A_ (*n* = 3 biological replicates). Data are represented as mean ± SD. (I) A model of YLDV 151R-mediated restriction of IFN-β signaling. Briefly, in the absence of YLDV 151R (left), IRF9 is free to associate with STAT1 and STAT2 following IFN-β stimulus to form a complete ISGF3 complex that translocates to the nucleus to activate ISGs. YLDV 151R expression (right) results in YLDV 151R binding and sequestering IRF9, preventing its interaction with STAT1 and STAT2, thus resulting in an incomplete ISGF3 complex. This image was generated with BioRender. Statistical comparisons were performed with a two-way ANOVA with pairwise comparisons via Tukey’s multiple comparisons test, with significant results between IFN-treated samples noted: **** *adj p* < 0.0001, ** *adj p* < 0.01, and * *adj p* < 0.05 (A, D, and H). See also Figure S7.

*Yatapoxviruses* are a poorly characterized genus of the Poxviridae family of double-stranded DNA viruses that replicate in the cytoplasm. YLDV (also known as tanapox virus) was implicated in two epidemics in the Tana River valley in Kenya and subsequent spillover events in several sub-Saharan African countries, Spain, and the United States.^56–58^ YLDV 151R and Yaba monkey tumor virus (YMTV) 151R and 1L scored in the IFN-β screen and were predicted to be homologs of VACV C10L/C16L.^59–61^ VACV C10L/C16L is part of the poxvirus C4/C10 protein family, which is characterized as an inhibitor of Ku70/80-dependent DNA sensing and indeed a sequence identity search clusters YLDV 151R and YMTV 151R in this family (Figure S7A).^61–64^ None of the four other *Orthopoxvirus* vORFs from the C4/C10 family in the viral ORFeome—VACV C4L, VACV C10L, Mpox D13L, and CPXV022—scored in our screen (Figure S7B; Table S3). Moreover, we validated that YLDV 151R and YMTV 151R, but not VACV C4L, VACV C10L, or Mpox D13L, impaired IFN-β-mediated MHC-I induction when assayed individually (Figure S7C). While both YLDV 151R and VACV C16L were able to partially inhibit *IFNB1* mRNA expression induced by DNA sensing (Figure S7D),^63–65^ of these two, only YLDV 151R was able to antagonize IFN-β signaling, blocking the induction of multiple ISGs (Figures 7A and S7E). Further validating our screens, the ability to inhibit IFN signaling was limited to IFN-β, with no impact of IFN-γ-mediated *IRF1* induction (Figure S7F).

To explore the YLDV 151R IFN-β restriction mechanism, we first examined the JAK-STAT pathway and found that YLDV 151R did not block STAT1 or STAT2 phosphorylation or phospho-STAT1 nuclear translocation (Figures S7G and S7H). Mass spectrometry revealed that a functional YLDV 151R interacted with Ku70, Ku80, and IRF9, the latter being a component of the ISGF3 complex that is formed in response to IFN-β stimulus alongside STAT1 and STAT2 (Figures S7I and S7J; Table S7).^63,64^ YLDV 151R expression also increased the level of IRF9, potentially impacting IRF9 turnover in the cell (Figure S7G). The ability of YLDV 151R to bind and sequester IRF9 could explain its specificity for inhibiting IFN-β signaling, as IFN-β, but not IFN-γ, utilizes IRF9 in its downstream signaling.^66^

The predicted structure of YLDV 151R is markedly similar to the structure of VACV C10L/C16L, each containing N- (NTD) and C-terminal domains (CTD) connected by a short linker (Figure 7B). Despite similarities, YLDV 151R, but not VACV C10L/C16L, immunoprecipitated with IRF9, explaining their differential ability to suppress IFN-β signaling (Figure 7C). Expressing these domains independently revealed that the N-terminal domain was necessary and sufficient to completely ablate IFN-β signaling (Figure 7D). Immunoprecipitation identified that IRF9 interacted specifically with the N-terminal domain, whereas Ku70 and Ku80 interacted with the C-terminal domain (Figure 7E).

The impact of the interaction between IRF9 and YLDV 151R can be observed at both steady state and following IFN-β treatment. YLDV 151R expression disrupts the steady-state interaction between IRF9 and STAT2, which facilitates the constitutive expression of a subset of ISGs, and prevents the formation of an intact ISGF3 complex following IFN-β stimulation, with IRF9 losing its ability to interact with the STAT1/STAT2 heterodimer (Figure 7F).^67,68^ AlphaFold 3 predicted structures of YLDV 151R binding to IRF9 suggest that it could occlude the STAT2 binding site on the IRF9 IRF association domain (IAD), thereby preventing interaction (Figure 7G).^69,70^ To define the mechanism of interaction, we found that expressing the IRF9 IAD was sufficient to mediate the YLDV 151R-IRF9 interaction (Figures S7K and S7L).^67^

Analyzing the YLDV 151R NTD-IRF9 IAD predicted structure with ePDB Proteins, Interfaces, Structures, and Assemblies (PISA) identified several potential interacting residues.^71^ This included two glutamic acid residues, E23 and E24, predicted to form a network of interactions between the YLDV 151R NTD and the IRF9 IAD (Figure 7G). Mutagenesis of these residues revealed that E23A mutants were partially defective, E24A mutants were strongly defective, and the double mutants were completely defective at suppressing *ISG15* induction (Figure 7H). Consistent with this, these mutants displayed a marked reduction in their ability to coimmunoprecipitate with IRF9, while retaining their interactions with Ku70 and Ku80 (Figure S7M). These findings support a model in which YLDV 151R binds IRF9 and impairs its interaction with both basal STAT2 and IFN-β-induced STAT1/STAT2 heterodimers, thereby preventing the formation of an intact ISGF3 complex and downstream transcription of ISGs (Figure 7I).

## DISCUSSION

The generation of a viral ORF library and its use to dissect viral regulation of host cellular pathways represents a comprehensive systems-level approach to understanding host-virus interactions. While traditional genetic and biochemical approaches have yielded critical insights into viral protein function, they often fail to capture the full diversity of the virome. Likewise, other efforts to develop collections of vORFs from across the virome typically have focused on a subset of viral ORFs with predicted phenotypes, prioritized a subset of pathogens of interest, or were designed without features (such as diversified barcodes, simple stop codon removal for C-terminal fusions, simple subcloning mechanisms, T7 *in vitro* translation) that enable robust and versatile pooled genetic screening. In addition, commercial viral ORF collections are prohibitively expensive for applications at scale and confined to a smaller subset of viral pathogens. The scale and diversity of this viral ORF library, initially consisting of ~12,000 viral ORFs from >500 viral species (which we recently expanded to ~13,400), cover most of the known mammalian viral proteome. This size, together with the versatile design of its vector, makes it particularly powerful, enabling the identification of vORF homologs that either conserve or diverge in their functions via pooled genetic screens, providing a broad evolutionary perspective on the genes identified by its use.

The viral ORFeome includes many understudied species, enabling the characterization of vORFs with no previously known function or homology. Our initial screen for regulators of cell proliferation showed that the majority of viral ORFs exhibit deleterious activities, consistent with viruses disrupting critical cellular processes while hijacking them for their own purposes. Strikingly, we observed strong cell-type specificity, reflecting viral adaptation to diverse cellular environments, which may also be linked to their diverse viral tropisms. For example, HIV exists primarily in CD4^+^ T cells, and genetic studies have shown that it depends on genes expressed in CD4^+^ T cells for its replicative fitness.^72^ We note that each toxic viral interaction represents a potential therapeutic target for blocking viral functions and restoring normal cellular activity.

Our examination of antigen presentation and IFN stimulus unveils functions central to the virus’s evolutionary pressure to evade the immune system. Several key observations emerged. First, we identified many previously known regulators of these pathways, validating the robustness of our high-throughput screening approach. Secondly, we discovered numerous previously uncharacterized modulators of these pathways, many in understudied viruses. Thirdly, using sequence similarity searches, we demonstrated that evolutionarily related proteins share similar phenotypes, further reinforcing the power of this library approach. Finally, while most proteins are annotated with a particular function, many exhibit additional distinct activities. This highlights the notion that the surfaces of viral proteins are evolutionarily sculpted to carry out many distinct roles, maximizing function within a limited proteomic space.

Immune evasion through reduced detection by the adaptive immune system can occur through diverse mechanisms, but many questions remain. For example, MC162R appears to recognize most HLAs; it remains unclear whether it acts on the plasma membrane, on ER-localized HLA, or on both. Nor do we know exactly how it recognizes MHC class I, potentially via β2M or some common region of the different HLA proteins. Furthermore, the proteomic analyses suggested that additional proteins may be strongly reduced by MC162R, including SPRY4, CALR, and TFRC, among others. The significance of these relationships to viral function remains to be determined. IFN signaling, a barrier to viral replication, is also frequently targeted by viruses. We found 118 candidate viral proteins that specifically inhibit IFN-β and/or IFN-γ signaling. Of these, >60 vORFs had not been previously implicated in IFN antagonism. We focused on two poxvirus vORFs from the relatively uncharacterized *Yatapoxvirus* genus, YLDV 151R and YMTV 151R, both of which specifically impaired IFN-β signaling.

Interestingly, both MC162R and YLDV 151R belong to the family Poxviridae but lie outside of the well-characterized *Orthopoxvirus* genus. Virome-wide vORF studies thus provide a unique opportunity for the identification of viral genes from understudied species that are not classified as priority pathogens but can impact human protein function. These viral proteins from understudied species can be sequence and structural homologs to characterized viral proteins that nonetheless diverge in their function, as YLDV 151R does, or can have previously unknown functions, as in the case of MC162R. These functions are important because viruses within the same family can exchange genetic material (e.g., via recombination or reassortment), potentially transferring genes from less-studied viruses to human pathogens.^73^ Likewise, viruses that are primarily found in animals but capable of manipulating human immunity may pose zoonotic threats to human health.

The utility of this library goes beyond single vORF functional screens. Future applications should include comprehensive proteomic and transcriptomic analyses to map vORF interactomes and to elucidate global effects on cellular physiology, thereby guiding the way toward a system-level understanding of virus-host interactions. While imperfect, standardized, flexible viral ORFeome resource empowers the community by providing functional insights, facilitating tool development, and enabling new research directions to decipher viral mechanisms and vulnerabilities, thereby reducing experimental barriers and jump-starting scientists in new directions to understand viral protein function.

### Limitations of the study

Despite its utility, viral ORF libraries have several limitations. They require continual updating as new viral genes are discovered. In addition, technical constraints in gene synthesis and lentiviral delivery limit the inclusion of very large proteins. Because this approach examines viral proteins individually, it enables mechanistic insight but may miss functions dependent on multi-protein complexes. This library should therefore be viewed as an evolving resource that will improve over time. Additional limitations include the lack of robust cell culture or reverse genetics systems for many of the included viruses, and the use of expression systems that may not directly represent the levels of expression observed during viral infection. Nonetheless, these constraints do not diminish the value of vORFs as tools to probe host pathways or to identify viral proteins that have evolved to manipulate cellular processes.

## RESOURCE AVAILABILITY

### Lead contact

Further information and requests for reagents may be directed to the lead contact, Stephen J. Elledge (selledge@genetics.med.harvard.edu).

### Materials availability

Materials generated in this study will be made available upon request. The viral ORFeome will be deposited to Addgene in a pooled format.

### Data and code availability

The mass spectrometry proteomics data have been deposited to the ProteomeXchange Consortium via the PRIDE partner repository with the dataset identifiers PXD077896 (related to Figure 5B; Table S5), PXD077768 (related to Figure S5F; Table S6), and PXD077910 (related to Figure S7J; Table S7). Other data will be made available upon request.

## STAR★METHODS

### EXPERIMENTAL MODEL AND STUDY PARTICIPANT DETAILS

#### Cell lines and culturing

All cell lines used in this study are detailed in the key resources table. HEK-293T cells obtained from the American Tissue Culture Collection were maintained in DMEM supplemented with 10% fetal bovine serum (FBS) (v/v), 100 units/mL penicillin, and 100 μg/mL streptomycin. A375 cells were obtained from the American Tissue Culture Collection and maintained in DMEM supplemented with 10% FBS, 100 units/mL penicillin, and 100 μg/mL streptomycin. HCECs were maintained in DMEM supplemented as previously described.^74^ RPE1 cells were maintained in DMEM/F12 medium supplemented with 10% FBS, 100 units/mL penicillin, and 100 μg/mL streptomycin. THP-1 cells were maintained in RPMI-1640 (ATCC) supplemented with 10% FBS (v/v), 100 units/mL penicillin, and 100 μg/mL streptomycin.

### METHOD DETAILS

#### Viral ORF library design and construction

##### vORF sequence curation

All viral species with human tropism were downloaded from the UniRef90 protein sequence collection UniProt and collapsed on 90% sequence identity. We then created fragments of up to 570 amino acids in length by tiling through the sequences collected, such that each sequence smaller than 570 amino acids was designed as a full fragment and sequences greater than 570 amino acids were designed as a series of 570 amino acid fragments with 285 amino acid overlap between the tiles. These fragments were computationally translated and codon optimized to enable expression in eukaryotic cells. A series of synonymous mutations were made in the vORF sequences to remove restriction sites used for downstream cloning (*Eco*RI, *Hind*III, *Bsm*BI, *Bam*HI, *Spe*I, *Sbf*I, and *Not*I, as well as the meganuclease sites) from all vORF sequences. Additional codon optimization and redesign occurred to ensure compatibility for synthesis in line with TWIST Bioscience’s guidelines for synthesis (i.e., reducing GC content and eliminating repetitive sequences, where possible). In the case of smaller vORF constructs, multiple constructs were combined to form concatenated constructs.

A unique barcode was appended to the 3‘ end of each vORF sequence prior to synthesis. These barcodes were designed to maintain an open reading frame within the vORF. In the case of vORFs that were synthesized individually or are the first vORF in a concatenated construct, the vORF was synthesized with a stop codon and a forward PCR primer site for barcode sequencing included in the synthesis prior to the barcode; for subsequent vORFs in concatenated constructs, these sites had to be inserted later.

##### Vector design and synthesized fragment insertion

pFuji101 was generated by PCR amplification of three fragments from pBR322 and pENTR-dTOPO isolation of those fragments, and reassembly via NEBuilder HiFi DNA Assembly (E2621S, New England Biolabs). The reverse PCR primer site for next-generation sequencing of vORF barcodes was synthesized as oligonucleotides that were annealed and phosphorylated with T4 PNK. The reverse PCR primer binding site was inserted by digesting the assembled vector with *Eco*RI, dephosphorylating it via CIP treatment, and ligation of the phosphorylated oligonucleotide cassette with T4 ligase.

To insert synthesized constructs, pFuji101 was linearized and each synthesized construct was inserted into pFuji101 by TWIST Biosciences. Individual clones were sequenced by TWIST Biosciences to confirm the orientation and sequence of the inserts and arrayed in 96-well plates. Upon receipt, each construct was transformed into DH5a E. coli. Vectors from up to four plates were pooled to form subpools of viral ORF vectors and between two and four subpools were combined to form pools that served as the basis for subcloning the viral ORF library.

##### Subcloning the viral ORFeome: Constructing single-vector constructs

The first vORF in each synthetic construct was isolated by digestion with *Eco*RI-HF, ligation with T4 ligase, and then counter-selection with *Hind*III-HF digestion prior to transformation. The plasmid DNA was then electroporated into ElectroMAX DH10B electrocompetent cells (Thermo, 18290015), recovered, and then plated onto kanamycin-containing LB agar plates. Following overnight culture, bacterial colonies were scraped and combined in LB broth, grown overnight in the presence of kanamycin, and then plasmid DNA was extracted.

Subsequent vORFs (positions 2–6) in each synthetic construct were isolated by *Bsm*BI digestion, followed by *Hind*III digestion. The digested product was then run on a 2% agarose gel and all fragments < 800 bp were gel purified as a pool. These fragments were then ligated into a *Bse*RI digested recipient vector with overhangs to enable vORF insertion in the proper orientation. These vectors were then selected for single vORF integration by digestion with *Hind*III and then transformed into DH10B electrocompetent cells, recovered, and then plated onto kanamycin-containing LB agar plates. Following overnight culture, bacterial colonies were scraped and combined in LB broth, grown overnight in the presence of kanamycin, and then plasmid DNA was extracted.

##### Subcloning the viral ORFeome: Inserting stop codons into subsequent vORF vectors

Single vORF vectors containing vORFs originally in positions 2–6 in the synthesized fragment had a stop codon inserted via a synthesized stop cassette. The stop cassette was amplified via PCR, digested with *Bsm*BI to generate compatible overhangs, isolated via agarose gel electrophoresis on a 2% agarose gel, and gel purified. Single vORF vectors were digested with *Spe*I and *Bam*HI and then treated with Quick CIP. The digested vectors were isolated via agarose gel electrophoresis on a 1.5% agarose gel, and extracted. The stop cassette was then ligated into the cut vectors, transformed into DH10B electrocompetent cells, recovered, and then plated onto kanamycin-containing LB agar plates. Following overnight culture, bacterial colonies were scraped and combined in LB broth, grown overnight in the presence of kanamycin, and then plasmid DNA was extracted.

##### Subcloning the viral ORFeome: Barcode diversification

For all vORFs, barcodes were diversified by annealing hemiphosphorylated oligonucleotides containing the barcode diversification cassettes. The four barcodes diversification cassettes were annealed in separate reactions by incubating the oligonucleotides at 95°C for 5 min and then reducing the temperature at a rate of 5°C/min until the temperature reached 25°C. Annealing reactions were combined such that the four additional barcodes were each present at 10 μM. The oligonucleotide sequences used for barcode diversification can be found in Table S8.

Each vORF pool was digested with *Eco*RI, then treated with Quick CIP (NEB, M0525S), heat inactivated, and purified via PCR purification (Macherey-Nagel). The digested vector was then digested with *Sbf*I and then purified via PCR purification as above. The digested vector and annealed barcode cassettes were mixed at a 1:10 ratio, ligated with T4 ligase, and then PCR purified. The purified vector was then selected by digestion with *Eco*RI, transformed into DH10B electrocompetent *E. coli*, recovered, and then plated onto kanamycin (50 μg/mL) containing LB agar plates. Following overnight culture, bacterial colonies were scraped and combined in LB broth, grown overnight in the presence of kanamycin, and then plasmid DNA was extracted. Sufficient bacterial colonies were collected in order to maintain 100x representation at the barcode level (~500x representation at the vORF level) within each pool.

##### Recombination into expression vector

Barcode diversified vORFs were mixed with vORFs containing the original barcode in a 4:1 ratio. The vORFs and their corresponding barcodes were introduced into pHAGE-TRE-DEST-*Pme*I via the LR reaction (Gateway^™^ LR Clonase^™^ II Enzyme mix, Invitrogen, 11791020). The vector was transformed into DH10B *E. coli* via electroporation and plated on ampicillin (100 μg/mL) containing LB agar plates. Bacterial colonies were collected at 500x representation at the vORF level and grown overnight in suspension in LB broth containing carbenicillin (100 μg/mL), then plasmid DNA was extracted.

##### Sequencing and analysis of the viral ORFeome collection

We amplified barcodes for Illumina sequencing from plasmid stocks using two rounds of PCR amplification using Q5 Hot Start polymerase. The first round of amplification used a staggered set of primers to amplify the barcodes from 50 ng of plasmid from each vORF packaging pool in a 100 μL reaction. The second round of amplification used 200 ng of purified PCR product from the first reaction and introduced a different unique indexing primer for each pool. After the second round of PCR, the PCR product was purified and mixed in equivalent ratios based on DNA concentration as determined via NanoDrop. The mixed samples were then subject to gel electrophoresis, gel extracted, and cleaned up with AmPure beads per the manufacturer’s protocol. Following clean up, the pooled barcode DNA was sequenced by the Harvard Medical School Biopolymers Facility using a 75 bp read cycle on an Illumina NextSeq 500. Barcodes were extracted from the sequencing reads using Cutadapt, which were then were aligned to the barcode reference via Bowtie2 to generate read counts.^79,81^

#### Plasmids and cloning

The Gateway entry clones for viral ORFs used in this study were obtained from the viral ORFeome collection described in this paper. The individual viral ORFs from the viral ORFeome that were used in this study are detailed in the key resources table. VACV WR C16L dsDNA was obtained from IDT DNA. The Gateway entry cDNA clone for IRF9 (Clone ID: IOH28745; Accession: NM_006084.4) was obtained from the Ultimate ORF Clone collection (ThermoFischer).

The following lentiviral Gateway destination vectors were used in this study: pINDUCER20, pHAGE-TRE-DEST-*Pme*I-Puro, pHAGE-TRE-3xHA-DEST-Puro, pHAGE-TRE-AlfaTag-DEST-puro, pHAGE-EF1-DEST-NAT, pHAGE-TRE-DEST-NAT-CD52, pHAGE-TRE-FlagHA-DEST-Puro, pHAGE-EF1-FlagHA-DEST-Puro, pHAGE-TRE-BirA*-DEST-Neo. Briefly, to generate expression clones from destination vectors, entry vector plasmid DNA was incubated with the indicated destination vector with LR Clonase II per the manufacturer’s instructions and transformed into *Stbl3* chemically competent *E. coli*. All Gateway reaction products were verified by whole-plasmid sequencing (Plasmidsaurus).

YLDV 151R and IRF9 truncation mutants were generated by PCR with primers corresponding to the region of interest featuring an N-terminal FLAG tag and attB1 and attB2 sites that would append the appropriate sequences to the 5‘ and 3‘ ends of the PCR product. MC162R and YLDV 151R point mutants were generated by overlap extension PCR with primers that corresponding to the region of interest featuring an N-terminal FLAG tag and attB1 and attB2 sites that would append the appropriate sequences to the 5‘ and 3‘ ends of the PCR product. PCR products were recombined into pDONR223 with BP Clonase II and then into the relevant DEST vector with LR Clonase II. The following truncation and point mutants were generated for this study:

YLDV 151R NTD: M_1_-K_172_ of YLDV 151R with an N-terminal FLAG tag.

YLDV 151R CTD: V_176_-I_333_ of YLDV 151R with an N-terminal FLAG tag.

YLDV 151R_E23A_: Full length Flag-YLDV 151R with an E23A mutation.

YLDV 151R_E24A_: Full length Flag-YLDV 151R with an E24A mutation.

YLDV 151R_E23A-E24A_: Full length Flag-YLDV 151R with E23A and E24A mutations.

IRF9 DBD: M_1_-V_120_ of IRF9 with an N-terminal FLAG tag.

IRF9 IAD: S_182_-V_393_ of IRF9 with an N-terminal FLAG tag.

MC162R PA1: Full length MC162R with P474A, P475A, and P476A mutations.

MC162R PA2: Full length MC162R with P481A, P482A, and P483A mutations.

MC162R PA1 + PA2: Full length MC162R with P474A, P475A, P476A, P481A, P482A, and P483A mutations.

HLA-A2_TLM_: Full length HA-HLA-A2 with K336A, K340A, and K358A mutations.

Lentiviral CRISPR-Cas9 constructs utilized either the pLentiCRISPR v2, pLentiCRISPR v2-NAT, or pLentiCRISPR v2-EGFP plasmids. The vectors were digested with *Bsm*BI and gel purified. sgRNA oligonucleotides with the appropriate overhangs were synthesized by IDT, phosphorylated and annealed, and ligated into the relevant backbone. For the purposes of this study, 3 sgRNAs apiece against COPA, HGS, ITCH, TSG101, UBAP1, VHL, and VPS13D were cloned into pLentCRISPR v2, 3 sgRNAs apiece against ITCH, NEDD4, NEDD4L, WWP1, and WWP2 were cloned into pLentiCRISPR v2-NAT, and 3 sgRNAs apiece against NEDD4, NEDD4L, WWP1, and WWP2 were cloned into pLentiCRISPR v2-EGFP. The sgRNA target sequences used in this study are provided in Table S8.

#### Lentivirus production and titering

For packaging of the viral ORFeome and CRISPR libraries into lentivirus, HEK-293T cells were seeded in 15 cm tissue culture dishes at a density of 12×10^6^ cells per dish. Library DNA and four packaging plasmids (VSV-G, gag-pol, tat, and rev) were diluted in a 4:1:1:1:1 ratio in serum and antibiotic-free DMEM, with a total of 12 mg of DNA per transfection. DNA was transfected with PolyJet transfection reagent per the manufacturers protocol (utilizing a 3:1 ratio of PolyJet transfection reagent (μL) to plasmid DNA (μg). After ~12 h, the media was refreshed and then the supernatant was harvested 24–48 h later, filtered through a 0.45 μm filter and stored at −80° C. For packaging individual expression vectors, HEK-293T cells were seeded in 6-well tissue culture plates at a density of 8×10^5^ cells per well. Lentiviral expression vectors, psPAX2 (500 ng per transfection) and pMD2.G (50 ng per transfection), and expression vector plasmid DNA (500 ng per transfection) were diluted in serum and antibiotic-free DMEM and incubated with PolyJet transfection reagent diluted in serum and antibiotic-free DMEM for 10 m and then added to cells. After ~12 h, the media was refreshed and 24–48 h later the supernatant was harvested, filtered through a 0.45 μm filter and stored at −80°C.

To titer lentivirus, the cell line of interest was transduced with serial dilutions of lentivirus in the presence of polybrene (8 μg/mL) and incubated for 48 h. Following incubation, media was replaced with puromycin (2 μg/mL) media and cells were maintained in puromycin until such a time as colonies were visible (typically 5–7 d). Colonies were then stained with crystal violet and counted to determine lentiviral titer. For lentiviral infections, cells were transduced with lentivirus in the presence of polybrene (8 μg/mL), recovered for at least two days, and then selected with the relevant antibiotic for at least 3 d. For selection, the following antibiotics were used: puromycin (2 μg/mL), nourseothricin (NTC) (200 μg/mL), and hygromycin B (250 μg/mL).

#### Viral ORFeome proliferation screens

##### Conduct of screens

For proliferation screens, HCEC and RPE1 cells were transduced with a lentiviral vector encoding pINDUCER20-mCD19 and ping-pong sorted via positive and negative selection to achieve a population with uniform CD19 expression following treatment with doxycycline (200 ng/mL).^21^ A375 cells were transduced with a lentiviral vector encoding pINDUCER20-BFP. For ping-pong sorting, expression of BFP or mCD19 was induced with doxycycline (200 ng/mL) and the top 10% of cells expressing BFP or mCD19 were isolated via FACS; after passage in media without doxycycline for several days, cells were then sorted to isolate BFP or mCD19 negative cells. For A375 pINDUCER20-BFP cells, single cell clones were isolated via single-cell sorting and expanded.^21^

The A375 cells expressing pINDUCER20-BFP or the HCEC and RPE1 cells expressing pINDUCER 20-mCD19 were transduced with the viral ORFeome collection at an MOI < 0.3 at a representation of at least 1,000X in the presence of polybrene (8 μg/mL). Each cell line was transduced independently in duplicate. Media was replaced with complete growth media appropriate for the cell line without polybrene following incubation with lentivirus overnight. Approximately 48 h following transduction, the cells were split into media containing puromycin (2 μg/mL) and selected for at least 48 h with regular passaging, until such a time that a control plate of cells was killed. Cells were then expanded.

For the A375 and RPE1 proliferation screens, an input sample from each replicate of transduced cells with sufficient cells for at least 1,000X representation was collected on day 0, before vORF induction with doxycycline. A375 and RPE1 cells were then separated into doxycycline-induced and non-induced arms and doxycycline (200 ng/mL for A375; 100 ng/ml for RPE1) was added to complete growth media to induce vORF expression in the doxycycline-induced arm. The cells were passaged regularly and counted to calculate population doublings. After approximately five to seven population doublings, sufficient cells for at least 1,000X representation were collected from the uninduced and doxycycline-induced arms. For the HCEC proliferation screen, cells were induced with doxycycline (100 ng/mL) on day 0 and an input sample equivalent to at least 1,000X representation was collected on day two. Cells were passaged in the presence of doxycycline for a total of seven days, at which point sufficient cells for at least 1,000X representation of the vORF collection were collected.

##### gDNA isolation, barcode amplification, next-generation sequencing, and analysis

Genomic DNA was extracted from each sample by incubation in lysis buffer (10 mM Tris-HCl pH 8.0, 10 mM EDTA, 0.5% SDS, and 0.5 mg/mL proteinase K) overnight, followed by phenol-chloroform extraction and ethanol precipitation. Barcodes were amplified using the previously described method for amplifying barcodes from the gDNA samples from the A375 input samples. Barcodes were adapted from the sequencing reads using Cutadapt, which then were aligned to the barcode reference via Bowtie2 to generate read counts, which were analyzed via MAGeCK.

Combined analysis of the three proliferation screens was conducted using Stouffer’s method to generate a Stouffer p-value which was then corrected for multiple hypotheses via the Benjamini-Hochberg method. Log_2_Fold Changes in vORF abundance were converted into z-scores for each screen and then averaged across the three screens to generate a log_2_fold change z-score. All analyses were performed at the vORF level across the three screens.

#### FACS-based viral ORFeome HLA screens

For FACS-based screens, A375 cells expressing pINDUCER20-BFP were transduced with the viral ORFeome collection at an MOI < 0.3 at a representation of at least 1,000X in the presence of polybrene (8 μg/mL). Each cell line was transduced independently in duplicate. Media was replaced with complete growth media appropriate for the cell line without polybrene following incubation with lentivirus overnight. Approximately 48 h following transduction, the cells were split into media containing puromycin (2 μg/mL) and selected for at least 48 h with regular passaging, until such a time that a control plate of cells was killed. Cells were then expanded.

A375 cells harboring the viral ORFeome were seeded at least 1,000X representation of the viral ORFeome and expression was induced with 200 ng/mL doxycycline. After 48 h of doxycycline induction, the basal MHC expression arm of the screen was collected for sorting. After 48 h of doxycycline induction, the IFNβ and IFNγ-induced MHC expression arms of the screen had the media refreshed with complete DMEM containing 200 ng/mL doxycycline and either recombinant IFNβ (10 ng/mL) or recombinant IFNγ (10 ng/mL). Sixteen-eighteen h after beginning treatment with the indicated IFN, the cells were sorted and collected for sequencing. In all cases, an untreated input sample that was not sorted was collected that was equal to 1,000X representation of the library.

For FACS sorting, cells were stained with APC-conjugated human HLA-A2 antibody per the manufacturer’s instructions and FACS sorted. For FACS sorting, cells were sorted into four equally sized bins, each equal to 10% of the cellular population. Bins were arrayed in such a way that the top and bottom 10% of the population were collected and then two additional bins that were separated by 20% of the population from the top and bottom bins were collected. Sufficient cells were sorted to ensure at least 1000X representation of the viral ORFeome (~1.3×10^7^ cells per screen). Each screen was completed in duplicate. The sorted cell pellets were then lysed by repeated freeze-thawing and stored at −80°C. Genomic DNA was extracted as in the proliferation screens or via silica-based spin column extraction per the manufacturer’s instructions and barcodes were amplified using an identical protocol.

##### Analysis of screen results

Barcodes were isolated with Cutadapt and then aligned to the viral ORFeome barcode reference using Bowtie. Read counts within each bin were adjusted for sequencing depth and then an expression score was calculated, equivalent to summing the products of the fraction of reads for a given barcode in each bin by the number of that bin (1–4), generating an expression score between 1 and 4, using the following equation,

$$\mathrm{Expresson}\;\mathrm{Score}=\sum_{i=1}^{4}i\times fi,$$

where *i* is the bin number and *fi* is the fraction of reads for an individual barcode in that bin. For statistical analysis, a Z-score was calculated for each barcode and an aggregate Z-score was calculated for each vORF based on the corresponding barcode Z-scores using both Stouffer’s method and an average Z-score method. P-values for all Z-scores were calculated using a two-tailed normal distribution.

#### Ubiquitin sublibrary CRISPR screen

A375 cells were transduced with pHAGE-EF1-MC162R-NAT lentivirus, recovered for two days, and then selected with NTC for one week to generate a population of A375 cells that constitutively express MC162R. These cells were transduced with a CRISPR library targeting ubiquitin-proteasome system associated genes at an MOI < 0.3 at a representation of at least 1,000X in the presence of polybrene (8 μg/mL).^87^ Two days following transduction the cells were plated into puromycin (2 μg/mL) containing complete media and selected until such a time as an untransduced cell line was completely killed (~2–3 d). Cells were then expanded.

Following expansion, the cells were stained with APC-conjugated human HLA-A2 antibody per the manufacturer’s instructions and FACS sorted. For FACS sorting, cells were sorted into a single bin equal to the top 5% of the APC+ cellular population. Sufficient cells were sorted to ensure at least 1000X representation of ubiquitin CRISPR library at the sgRNA level (~1×10^7^ cells per replicate). The screen was completed in triplicate. The sorted cell pellets were then lysed by repeated freeze-thawing and stored at −80°C. Genomic DNA was extracted as in the HLA screen. sgRNAs were amplified, prepared for sequencing, and sequenced as described above.

#### Flow cytometry and FACS

All flow cytometry experiments were conducted on a CytoFLEX LX flow cytometer (Beckman Coulter) using CytExpert software. All fluorescence activated cell sorting experiments were conducted on a Sony MA900. All data were analyzed using FlowJo v10.

In general, for HLA-A2, PD-L1, HER2, B2M, and HA staining experiments, cells were washed with PBS and trypsinized. Trypsin was neutralized with complete media and cells were centrifuged (1000 x g), washed with PBS, centrifuged (1000 x g), and incubated with the relevant fluorophore-conjugated antibody. After 30 min of incubation, the cells were centrifuged, washed twice with PBS, and then analyzed or sorted. The following fluorophore-conjugated antibodies were used in this study: HA-Tag (6E2) Mouse mAb (Alexa Fluor^®^ 647 Conjugate) (CST, #3444), allophycocyanin-conjugated (APC) anti-human β2-microglobulin Antibody (Biolegend, #316312), APC anti-human HLA-A2 Antibody (Biolegend, #343308), APC anti-HLA-A,B,C Antibody (Biolegend, #311410), APC anti-CD274 (PD-L1) Antibody (Biolegend, #329708), APC anti-CD340 Antibody (Biolegend, #324408), APC anti-Erbb3 Antibody (Biolegend, #324707), and APC anti-CD54 Antibody (Biolegend, #353111).

For drug treatment experiments with MC162R, cells were pretreated with bafilomycin A1 (100 nM) (CST, #54645S), MG-132 (2 μM) (Selleck, S2619), TAK-243 (2 μM) (Selleck, S8341) for 2 h and then induced with doxycycline (200 ng/mL) in the presence of the indicated molecule. For heclin experiments, cells were pretreated with heclin (50–200 μM) (Selleck, E1216) for 12 h and then media was replaced with fresh media containing heclin at the indicated concentration and doxycycline (200 ng/mL). For all small molecule inhibitors, cells were analyzed via flow cytometry 12–14 h after induction of MC162 expression with doxycycline.

For siRNA experiments, sets of four siRNAs against COPA (Dharmacon, LQ-011835–00-0002), ITCH (Dharmacon, LQ-007196–00-0002), TSG101 (Dharmacon, LQ-003549–00-0002), VHL (Dharmacon, LQ-003936–00-0002), and VPS13D (Dharmacon, LQ-021567–02-0002) were diluted in siRNA buffer (Dharmacon, B-002000-UB-100) to 20 μM. siRNA:lipid complexes were formed using Lipofectamine RNAiMAX (Thermo, 13778075) per the manufacturer’s instructions. Cells were reverse transfected with siRNA, the media was changed 24 h later, and then cells were incubated for 48 h. After incubation, MC162R expression was induced with doxycycline for 12 h. Cells were then stained as above and analyzed via flow cytometry. For sgRNA experiments, cells were transduced with the indicated pLentiCRISPR v2 constructs, incubated for 2 days, selected as above, and then passaged. All sgRNA experiments were performed within two weeks of lentiviral transduction. For experiments using doxycycline-inducible MC162R, MC162R expression was induced for 12 h followed by staining and analysis via flow cytometry.

For IFNβ (Peprotech, 300–02BC) or IFNγ (Peprotech, 300–02) incubations, vORF expression was induced for 48 h with doxycycline and then the media was replaced with media containing doxycycline (200 ng/mL) and the indicated IFN. IFNs were used at a final concentration of 10 ng/mL unless otherwise indicated. Cells were collected 12–16 h following addition of IFN and stained with the indicated antibody as above. Briefly, cells were washed once with PBS, stained with an APC-conjugated HLA-A2 antibody (5 μL/1 million cells) for 30 minutes, washed thrice with PBS, and analyzed immediately thereafter.

#### Immunoblotting

Whole cell lysates were prepared in RIPA buffer (Boston BioProducts, #BP-115X) containing 1X Halt Protease and Phosphatase Inhibitor Cocktail (Thermo, #78441). Samples were boiled at 95°C and clarified by centrifugation for at least 15 min at 4°C. Protein concentration was measured by Bradford assay. Protein lysates were loaded and run on 4–12% Bis-Tris gels and transferred to nitrocellulose membranes. Membranes were blocked in 5% non-fat dry milk in TBS-T for 1 h at room temperature. Primary antibodies were diluted in TBS-T and incubated overnight at 4°C. HRP-conjugated antibodies were used for detection with enhanced chemi-luminescence substrate (Perkin-Elmer, NEL104001EA). The following antibodies were used in this study: GAPDH (Santa Cruz Biotechnology, 1:1000), FLAG M2 (Sigma Aldrich F1804, 1:1000), β-tubulin (CST #2128, 1:1000), pSTAT1 (CST #9167, 1:500), pSTAT2 (CST #90740, 1:500), STAT1 (CST #14994, 1:1000), STAT2 (CST #72604, 1:500), IRF9 (CST #76684, 1:500), HA-tag (CST #3724, 1:1000), DYKDDDDK-tag (CST #14793, 1:1000), MX1 (CST #37849, 1:1000), IFITM1 (CST #13126, 1:1000), Ku70 (CST #4588, 1:1000), Ku80 (CST #2180, 1:1000), HLA-A (1:500), Vinculin (Sigma Aldritch #V9131, 1:1000), β2-microglobulin (Thermo #MA1–19141, 1:1000), ITCH (CST #12117, 1:500), and HRS (#15087, 1:1000).

#### Immunoprecipitation and mass spectrometry

For all immunoprecipitation experiments, A375 pINDUCER20 or HEK-293T pINDUCER20 cells harboring a doxycycline-inducible ORF or empty vector control had expression induced for 48 h with doxycycline (200 ng/mL). In the case of immunoprecipitation of FlagMC162R, media was replaced with media containing doxycycline (200 ng/mL) and bafilomycin A (250 nM) for the final 12 h of induction.

Cells were lysed with lysis buffer (25 mM HEPES pH 7.4, 150 mM NaCl, 5 mM EDTA, and 1% Triton X-100 supplemented with 1x Halt Protease and Phosphatase Inhibitor Cocktail. Lysates were rotated at 4C for 30 min before clarification by centrifugation for 15 min at 4°C. Following clarification, up to 10% of the total volume of the lysate was collected as an input and stored at −80C. Anti-FLAG (Sigma), anti-HA (Thermo), or ALFASelector magnetic beads were rinsed in lysis buffer and added to the remaining lysate. Beads were incubated with lysate for 2 h at 4°C. Immunoprecipitants were washed once in lysis buffer, transferred to a new tube, washed thrice with lysis buffer, and then transferred to a new tube. Beads were resuspended in 2X sample buffer (Invitrogen, NP0007) and boiled for 5 min. The supernatant was transferred to a new tube and then stored at −80°C before immunoblotting.

#### YLDV 151R IP-MS

Immunoprecipitated samples were prepared for mass spectrometry as previously described. Briefly, at least five 15 cm plates were seeded with 3 × 10^6^ A375 cells harboring a 3xHA-tagged vORF or empty 3xHA vector in complete media containing doxycycline (200 ng/mL). After 48 h, cells were washed once with PBS, lysed in 1 mL/plate ice cold IP lysis buffer (25 mM HEPES pH 7.4, 150 mM NaCl, 5 mM EDTA, 1% Triton X-100) supplemented with 1x Halt Protease and Phosphatase Inhibitor (Thermo), and scraped off the plate and collected. Cell samples were lysed for 30 min with end-over-end rotation and then the lysate was clarified via centrifugation for 15 min at 21,000 x g. Anti-HA magnetic beads (Pierce) were washed twice with lysis buffer, added to the clarified supernatant (100 mL bead slurry/sample), and samples were incubated for 2 h at 4°C. Following incubation, the supernatant was removed and beads were washed once in lysis buffer, transferred to a new tube, and then washed thrice in lysis buffer. Beads were then resuspended in elution buffer (10% SDS, 50 mM Tris pH 7.5) and boiled for 5 min at > 95°C.

Samples were reduced by incubation with 5 mM TCEP for 15 min at 55°C and then alkylated with 20 mM iodoacetamide for 30 min at room temperature. Samples were acidified with 2.5% phosphoric acid and quenched with 100 mM Tris pH 7.5 and 90% MeOH. Samples were then bound to an S-Trap Micro column (Protifi, CO2-micro-10) per the manufacturer’s protocol, washed thrice with quench buffer (100 mM Tris pH 7.5, 90% MeOH), and digested with trypsin (2 mg in 50 mM ammonium bicarbonate pH 8) overnight at 37°C. Peptides were eluted in 50 mM ammonium bicarbonate pH 8 via centrifugation and columns were washed with 0.2% formic acid in water followed by 50% acetonitrile in water via centrifugation. The eluate was dried under reduced pressure via SpeedVac and resuspened in 0.1% formic acid in water. LC-MS/MS data were acquired using a VanquishFlex LC directly interfaced with a Q Exactive mass spectrometer and analyzed with the FragPipe graphical user interface using the MSFragger algorithm as previously described.^88,89^

#### BioID and mass spectrometry

For BioID experiments, A375 cells expressing BirA*-Flag or BirA*-FlagMC162R were incubated with 250 nM bafilomycin A and 50 μM D-biotin (Thermo, #B20656) overnight, then dissociated via cell scraping and flash frozen in liquid nitrogen. Cell pellets were lysed in 1x RIPA buffer (Boston BioProducts) supplemented with Complete EDTA-free protease inhibitor for 30 min at 4°C, sonicated, and clarified via centrifugation at 28000 x g for 30 min. Clarified supernatant was then desalted and free biotin removed by passing the sample through a 7K MWCO Zeba Spin Desalting Column (Thermo, #89882). Streptavidin magnetic beads (Thermo, #88817) were washed in RIPA buffer and then a volume equivalent to 300 mL slurry was added to each sample and incubated overnight at 4°C with rotation. Beads were washed five times in 1x RIPA buffer and thrice in PBS, then resuspended in PBS.

Quantitative proteomics were performed at the Taplin Mass Spectrometry Facility at Harvard Medical School. Excised gel bands were subjected to an in-gel trypsin digestion procedure. Briefly, gel pieces were treated with acetonitrile for 10 m, acetonitrile was removed, and gel pieces were dried with a speed-vac. Gel pieces were rehydrated with 50 mM ammonium bicarbonate solution with 12.5 ng/mL sequencing-grade trypsin (Promega) and incubated for 45 minutes, after which typsin was removed and gel slices were incubated overnight at 37 C in 50 mM ammonium bicarbonate. Peptides were extracted by washing with 50% acetonitrile and 1% formic acid, dried in a speed-vac, and stored at 4 C until analysis.

Samples were reconstituted for analysis in a solution of 2.5% acetonitrile, 0.1% formic acid. Samples were loaded via a Famos auto-sampler (LC Packings) onto a nano-scale reverse-phase capillary column. Peptides were eluted with increasing concentrations of a solvent of 97.5% acetonitrile, 0.1% formic acid. Eluted peptides were detected in a Velos Orbitrap Elite mass spectrometer. MS/MS spectra were searched using the Sequest algorithm against a database of all human proteins in both the forward and reversed orientations.

#### RT-qPCR

For RT-qPCR, A375 pINDUCER20 cells harboring a viral ORF or empty vector control had ORF expression induced for 48 h with doxycycline (200 ng/mL). Following induction, cells were stimulated with either IFNβ (10 ng/mL), IFNγ (10 ng/mL), or the equivalent volume of PBS for 4 h in the presence of doxycycline (200 ng/mL). For cells transfected with poly(dA:dT) (1 μg/mL) or mock transfected using Lipofectamine 2000, cells were collected 6 h after transfection.

mRNA was extracted from cell pellets using the RNeasy Plus RNA Extraction Kit (Qiagen) per the manufacturer’s protocol. 500 ng of total RNA were used for cDNA synthesis using the iScript cDNA Synthesis Kit (Bio-Rad) per the manufacturer’s protocol. The cDNA reaction was diluted with 4 volumes of RNase-free water. qPCR reactions were run with 2 μL (12.5 ng theoretical yield) of cDNA using Platinum SYBR Green qPCR Supermix (Invitrogen). A QuantStudio 6 Pro (ThermoFisher Scientific) was used to run the qPCR reactions. The following pre-mixed Prime Time qPCR primers were obtained from IDT: Human *BST2* (Hs.PT.58.2711844), human *IFNB1* (Hs.PT.58.39481063), human *ISG15* (Hs.PT.58.39185901), human *IRF1* (Hs.PT.58.26847423), human *MX1* (Hs.PT.58.40261042), human *RSAD2* (Hs.PT.58.713843), and human *TBP* (Hs.58v.39858774). HLA-A qPCR was performed with TaqMan qPCR probes in a premixed assay (Thermo Fisher Scientific, Assay ID# Hs01058806_g1).

#### Immunofluorescence

For MC162R experiments, A375 pINDUCER20 cells harboring doxycycline-inducible FlagMC162R or an empty vector control were grown on coverslips overnight prior to doxycycline-induction (200 ng/mL) of ORF expression for 24 hours. After induction, media was replaced with media containing doxycycline (200 ng/mL) and bafilomycin A (250 nM) for 12 hours, at which point cells were washed with PBS and fixed. For YLDV 151R experiments, A375 pINDUCER20 cells harboring doxycycline-inducible FlagYLDV 151R or an empty vector control were grown on coverslips for 36 hours in the presence of doxycycline (200 ng/mL). After ORF induction, media was replaced with doxycycline-containing media with IFNβ (10 ng/mL) or the equivalent amount of PBS and cells were incubated 30 minutes at 37° C, followed immediately by washing with PBS and fixation.

Cells grown on coverslips were fixed for 15 min at room temperature in 3.7% formaldehyde diluted in PBS. Cells were then washed twice in PBS. For phosphor-STAT1 stains, cells were then permeabilized with ice-cold methanol for 2 min at −20° C and washed thrice with PBS. Cells were then permeabilized for 30 min in 0.25% Triton X-100, 0.3 M glycine in PBS and blocked for one h in 3% BSA in PBS with 0.1% Triton X-100. Cells were washed thrice in with PBS and then incubated with primary antibody diluted in PBS with 0.1% Triton X-100 and 3% BSA for 2 h at room temperature. Cells were washed thrice and then incubated with secondary antibody diluted in PBS with 0.1% Triton X-100 and 3% BSA for 1 h at room temperature in the dark. Coverslips were then mounted on slides with ProLong Gold Antifade Reagent with DAPI. The following antibodies were used in this study: HLA-A (Abcam ab52922), HLA-ABC (Biolegend 311402, 1:200) DYKDDDYK (CST #14793, 1:200), Lamp1 (CST #9091, 1:500), pSTAT1 (CST #9167, 1:200). Secondary antibodies used included AlexaFluor 488 goat anti-mouse IgG (Invitrogen, 1:1000), AlexaFluor 488 goat anti-rabbit IgG (Invitrogen, 1:1000), and AlexaFluor 647 goat anti-rabbit IgG (Invitrogen, 1:1000).

All coverslips were imaged on Axio Observer 7 (Zeiss) with Plan-Apochromat 63x/1.40 Oil DIC M27 objective (Zeiss, 420782–9900-000), Colibri 7 Type R[G/Y]CBV-UV light source (Zeiss, 423052–9741-000), and Axiocam 705 mono camera (Zeiss, 426560–9060-000). Images were processed using FIJI. Analysis was conducted using CellProfiler. HLA-ABC/MC162R and HLA-ABC/LAMP1 colocalization were quantified via calculation Manders coefficients on a per cell basis with CellProfiler. Phospho-STAT1_Y701_ localization and intensity were quantified via CellProfiler using a DAPI nuclear counterstains and a uniformly expanded perinuclear region to quantify the fraction of pSTAT1_Y701_ in the nucleus relative to the combined nuclear and perinuclear regions.

#### T cell killing assays

T cells were expanded as previously described.^78^ Briefly, apheresis collars were obtained from the Brigham and Women’s Hospital Specimen Bank. Peripheral blood mononuclear cells were purified and irradiated with 60 Gy IR. T cells were then cultured with irradiated PBMCs in RPMI-1640, supplemented with 10% FBS, 100 units/mL penicillin, and 100 mg/mL streptomycin, 50 units/mL IL-2, and 100 ng/mL anti-CD3 antibody for one week.

Following expansion, T cells were collected and co-cultured with A375 cells that had previously had viral ORF expression induced for 48 h prior to co-culture at a 1:1 or 1:5 ratio. Prior to co-culture, T cells were incubated with CellTrace^™^ carboxyfluorescein succinimidyl ester (CFSE) (Invitrogen #34554) and A375 cells were incubated with CellTrace^™^ Violet (Invitrogen #34557). Following 12 h of co-culture, cells were analyzed via flow cytometry. Briefly, the CellTrace^™^ Violet+ population was gated on, following by live/dead gating based on forward and side scatter. The ratio of live CellTrace^™^ Violet+ cells to total CellTrace^™^ Violet+ cells was then calculated. Biological replicates represent T cells from different expansions or assayed on different days in technical triplicates.

#### *In vitro* ITCH binding assay

BL21(DE3) cells expressing mouse ITCH (residues 143–864) in the pGEX vector with an N-terminal Glutathione S-transferase (GST) tag and 3C protease cleavage sequence, were induced with 0.5 mM IPTG for 16 h at 20°C. The cells were resuspended in lysis buffer (25 mM HEPES, pH 7.8, 250 mM NaCl, 1 mM TCEP) supplemented with Complete protease inhibitors (Sigma 11836170001) and lysed by French press. The clarified lysate was passed over GSH agarose resin (MClab GAB-300), washed once with lysis buffer supplemented with 0.1% Triton X-100 followed by a final wash with lysis buffer. The protein was eluted in lysis buffer containing 50 mM reduced glutathione. Eluted protein was incubated with 3C protease (GE 270843–0.5KU) and dialyzed against lysis buffer using a 3 kDa cutoff membrane. The dialyzed protein was passed over a GSH column to remove un-cleaved material and free GST, and the flow-through was concentrated and subjected to size exclusion chromatography on an S200 column. Fractions containing ITCH were concentrated and stored in lysis buffer supplemented with 10% glycerol.

Peptides corresponding to MC162R residues 469–489 (GHLPPPPPYCPVPPPYSDNTR) or the mutant MC162R-PA (GHLPPAAAA CPVAAAASDNTR), each with an N-terminal 5-FAM dye, were ordered from Genscript. MicroScale Thermophoresis (MST) was performed at the Center for Macromolecular Interactions, Harvard Medical School (RRID:SCR_018270). Labeled peptides (final concentration at 20 nM) were combined with varying concentrations of ITCH that were prepared using a two-fold serial dilution with lysis buffer supplemented with 5% glycerol, 1 mg/mL BSA, and 0.05% Tween 20. The samples were incubated at room temperature for 10 min for equilibration before being transferred into Monolith NT.115 standard capillaries (Nanotemper MO-K022) and analyzed using a Nanotemper Monolith NT.115pico instrument. The MST power was set to Medium and the laser power was set at 40%. Fluorescent intensity change was monitored for 20 s during the MST measurement and F_norm_ was calculated as the ratio of real-time fluorescence over initial fluorescence (F_0_). Binding affinity (Kd) was determined by plotting the concentration of ITCH and F_norm_ at t=1 s with a quadratic equation binding Kd model using MO. analysis (v3.2) software.

#### Whole-cell proteomics

For whole-cell proteomics, A375-pINDUCER20-BFP cells were transduced with vORFs in pHAGE-TRE-3xHA-DEST-PGK-puro. Following puromycin selection, cells were seeded in triplicate on 10 cm plates and allowed to reach >80% confluency before induction with 200 ng/mL DOX for 16 h. Cells were washed in ice-cold PBS and lysed in 8 M urea, 200 mM EPPS pH 8.5 supplemented with protease inhibitor (Pierce A32953) by passing through a 1.5 inch 21G needle for 10 strokes. Protein concentration was quantified using Pierce BCA assay (Thermo Fisher Scientific 23227) and normalized to 1 mg/mL. 100 mL of normalized lysate was reduced with 5mM TCEP for 15 min, alkylated with 10 mM iodoacetamide for 30 min in the dark, and quenched with 5 mM dithiothreitol (DTT) for 15 min. Protein was extracted via methanol-chloroform precipitation. The following were added sequentially, vortexing for 5 seconds between each addition: 400 mL methanol, 100 mL chloroform, 300 mL water. The samples were centrifuged for 1 min at 14,000xg before removal of organic and aqueous phases. The samples were washed once with 400 mL methanol and centrifuged at 21,000xg for 2 min. Pellets were resuspended in 200 mM EPPS pH 8.5 and treated with 1 mg Lys-C protease (FUJIFILM, 121–05063) overnight at room temperature. The following day, 1 mg trypsin (ThermoFisher Scientific, 90305) was added for 6 h at 37°C. Following digestion, samples were labeled with 200 mg TMT reagent (Thermo Fisher Scientific, A52045) in 30% acetonitrile for 1 h at room temperature, quenched with 0.3% hydroxylamine, desalted with C18 solid-phase extraction (Waters, Cat # WAT054925), and centrifuged under reduced pressure to dry.

#### Off-line high-pH reversed-phase (BPRP) fractionation

The pooled TMT-labeled peptides were fractionated using an Agilent 1260 HPLC system. Peptides were separated over a 560-min linear gradient from 5% to 35% acetonitrile in 10 mM ammonium bicarbonate (pH 8) at a flow rate of 0.25 mL/min, utilizing an Agilent 300Extend C18 column (3.5 μm particles, 2.1 mm ID, 25 cm length). The peptide mixture was divided into 96 fractions, which were then combined into 24 super-fractions for FAIMS-MS/MS analysis. Each super-fraction was acidified with 1% formic acid, concentrated to near dryness via vacuum centrifugation, desalted using StageTips, dried again, and reconstituted in 5% acetonitrile with 5% formic acid for LC-MS/MS analysis.

#### Mass spectrometric data collection

Mass spectrometric data were collected on an Orbitrap Ascend mass spectrometer coupled to a Vanquish Neo UHPLC for twelve non-adjacent superfractions. Approximately 1μg of peptide was separated at a flow rate of 450 nL/min on a 100 μm capillary column that was packed with 35 cm of Accucore 150 resin (2.6 μm, 150Å; ThermoFisher Scientific). The scan sequence began with an MS1 spectrum (Orbitrap analysis, resolution 60,000, 350–1350 Th, automatic gain control (AGC) target is set to 100%, maximum injection time set to 50 ms). Data were acquired 90 min per fraction. The hrMS2 stage consisted of fragmentation by higher energy collisional dissociation (HCD, normalized collision energy 36%) and analysis using the Orbitrap (AGC 200%, maximum injection time 120 ms, isolation window 0.6 Th, resolution 45,000). Data were acquired using the FAIMSpro interface the dispersion voltage (DV) set to 5,000V, the compensation voltages (CVs) were set at −40V, −60V, and −70V. The TopSpeed parameter was set at 1 sec per CV. Data analysis was performed as described previously.^90^

### QUANTIFICATION AND STATISTICAL ANALYSIS

#### Quantification of screen data

Quantification of data from the proliferation screens, MHC-I screens, and ubiquitin sublibrary CRISPR screen are described in the methods details above.

#### Analysis of flow cytometry data

Flow cytometry data was analyzed with FlowJo. In the case of quantification of MC162R-mediated changes in cell surface HLA-A2 expression (Figures 5C, 5D, S4J, S4K, S4D, and S4E), the fraction of HLA-A2^+^ cells is reported. In the case of quantification of IFN-induced increases in HLA-A2 expression (Figures 6H and S7C), the median fluorescence intensity (MFI) of the APC channel was measured and the ratio of IFN treated to untreated MFI_APC_ was taken. This ratio was log_2_ transformed and normalized to the empty vector control (EV = 1).

#### Analysis of qPCR data

Changes in mRNA levels were determined using the ΔΔCq method, with *TBP* serving as the housekeeping gene. In all instances, values were normalized to the untreated empty vector control. All assays were analyzed using a two-way ANOVA with multiple comparisons testing via Tukey’s multiple comparisons test (Figures 7A, 7D, 7H, S6G, S7D, and S7F). Statistical comparisons were performed via GraphPad Prism. All quantified qPCR assays were performed on three biological replicates.

#### Analysis of immunofluorescence data

All analysis of immunofluorescence data was conducted using CellProfiler following uniform image preprocessing in FIJI.^86^ (Figure 4C) HLA-ABC/MC162R and (Figure S4G) HLA-ABC/LAMP1 colocalization were quantified via calculation of Manders coefficients on a per cell basis with CellProfiler. The M_1_ coefficient (fraction of HLA-ABC colocalized with either MC162R or LAMP1) is reported. HLA-ABC/MC162R and HLA-ABC/LAMP1 colocalization assays were analyzed using a Mann-Whitney test. Phospho-STAT1_Y701_ localization and intensity were quantified via CellProfiler using a DAPI nuclear counterstain and a uniformly expanded perinuclear region to quantify the fraction of pSTAT1_Y701_ in the nucleus relative to the combined nuclear and perinuclear regions (Figure S7H). Phospho-STAT1_Y701_ localization assays were analyzed using a Kruskal-Wallis test with multiple comparisons testing via Dunn’s multiple comparisons test. Statistical comparisons were performed via GraphPad Prism.

## Supplementary Material

MMC1

MMC7

MMC2

MMc9

MMC4

MMC6

MMC3

MMC5

MMC8

SUPPLEMENTAL INFORMATION

Supplemental information can be found online at https://doi.org/10.1016/j.cell.2026.05.024.

## Figures and Tables

**Table:** KEY RESOURCES TABLE

REAGENT or RESOURCE	SOURCE	IDENTIFIER
Antibodies		

Recombinant sdAb anti-ALFA Rabbit Fc-fusion	NanoTag	Cat# N1583; RRID: AB_3075998
beta-2 Microglobulin Monoclonal Antibody (B2M-01)	Thermo Fisher Scientific	Cat# MA1–19141; RRID: AB_1070702
APC anti-human CD54, clone HA54	BioLegend	Cat# 353111; RRID: AB_10917389
APC anti-human CD340 (erbB2/HER-2), clone 24D2	BioLegend	Cat# 324408; RRID: AB_2262301
DYKDDDDK Tag (D6W5B) Rabbit mAb	Cell Signaling Technology	Cat# 14793S; RRID: AB_2572291
APC anti-human erbB3/HER-3, clone 1B4C3	BioLegend	Cat# 324707; RRID: AB_2099568
Monoclonal ANTI-FLAG^®^ M2 antibody produced in mouse	Sigma-Aldrich	Cat# F1804; RRID:AB_262044
Anti-GAPDH Antibody (G-9)	Santa Cruz Biotechnology	Cat# sc-365062; RRID: AB_10847862
HA-Tag (C29F4) Rabbit mAb	Cell Signaling Technology	Cat# 3724S; RRID: AB_1549585
Purified anti-human HLA-A,B,C, clone W6/32	BioLegend	Cat# 311402; RRID: AB_314871
APC anti-human HLA-A,B,C, clone W6/32	BioLegend	Cat# 311410, RRID: AB_314879
APC anti-human HLA-A2, clone BB7.2	BioLegend	Cat# 343308; RRID: AB_2561567
MHC class I (HLA-A) antibody [EP1395Y]	Abcam	Cat# ab52922; RRID :AB_881225
HRS (D7T5N) Rabbit mAb	Cell Signaling Technology	Cat# 15087; RRID: AB_2798700
ITCH (D8Q6D) Rabbit mAb	Cell Signaling Technology	Cat# 12117; RRID: AB_2797822
Ku70 (D10A7) Rabbit mAb	Cell Signaling Technology	Cat# 4588S; RRID: AB_11179211
Ku80 (C48E7) Rabbit mAb	Cell Signaling Technology	Cat# 2180S; RRID: AB_2218736
IFITM1 Antibody	Cell Signaling Technology	Cat# 13126S; RRID: AB_2798126
IRF-9 (D2T8M) Rabbit mAb	Cell Signaling Technology	Cat# 76684S; RRID: AB_2799885
LAMP1 (D2D11) XP Rabbit Antibody	Cell Signaling Technology	Cat# 9091; RRID: AB_2687579
MX1 (D3W7I) Rabbit mAb	Cell Signaling Technology	Cat# 37849S; RRID: AB_2799122
OAS1 (D1W3A) Rabbit mAb	Cell Signaling Technology	Cat# 14498; RRID:AB_2798498
Phospho-Stat1 (Tyr701) (58D6) Rabbit mAb	Cell Signaling Technology	Cat# 9167S; RRID: AB_561284
Stat1 (D1K9Y) Rabbit mAb	Cell Signaling Technology	Cat# 14994S; RRID: AB_2737027
Phospho-Stat2 (Tyr690) (D3P2P) Rabbit mAb	Cell Signaling Technology	Cat# 88410S; RRID: AB_2800123
Stat2 (D9J7L) Rabbit mAb	Cell Signaling Technology	Cat# 72604S; RRID: AB_2799824
Mouse Anti-Vinculin Monoclonal Antibody, Unconjugated, Clone hVIN-1	Sigma-Aldrich	Cat# V9131; RRID: AB_477629
APC Mouse IgG1, κ Isotype Ctrl, clone MOPC-21	BioLegend	Cat# 400119; RRID: AB_2888687
Goat anti-Rabbit IgG (H+L) Secondary Antibody, HRP	Thermo Fisher Scientific	Cat# 31460; RRID: AB_228341
Goat anti-Mouse IgG (H+L) Secondary Antibody, HRP	Thermo Fisher Scientific	Cat# 31430; RRID: AB_228307
Goat anti-Mouse IgG (H+L) Highly Cross-Adsorbed Secondary Antibody, Alexa Fluor^™^ Plus 488	Thermo Fisher Scientific	Cat# A32723; RRID: AB_2633275
Goat anti-Rabbit IgG (H+L) Highly Cross-Adsorbed Secondary Antibody, Alexa Fluor^™^ Plus 488	Thermo Fisher Scientific	Cat# A32731; RRID: AB_2633280
Goat anti-Rabbit IgG (H+L) Highly Cross-Adsorbed Secondary Antibody, Alexa Fluor^™^ Plus 647	Thermo Fisher Scientific	Cat# A32733; RRID: AB_2633282

Bacterial and virus strains		

One Shot^®^ Stbl3^™^ Chemically Competent E. coli	Thermo Fisher Scientific	Cat# C7373–03
DH5α competent *E. coli*	ThermoFisher Scientific	Cat# EC0111
ElectroMAX^™^ DH10B^™^ Cells	Thermo Fisher Scientific	Cat# 18290015
BL21(DE3) Cells	Thermo Fisher Scientific	Cat# C606010

Chemicals, peptides, and recombinant proteins		

Quick CIP	New England Biolabs	Cat# M0525L
T4 DNA Ligase	New England Biolabs	Cat# M0202M
T4 Polynucleotide Kinase	New England Biolabs	Cat# M0201L
EcoRI-HF	New England Biolabs	Cat# R3101T
HindIII-HF	New England Biolabs	Cat# R3104T
BseRI	New England Biolabs	Cat# R0581L
BsmBI-v2	New England Biolabs	Cat# R0739L
SpeI-HF	New England Biolabs	Cat# R3133M
SbfI-HF	New England Biolabs	Cat# R3642L
BamHI-HF	New England Biolabs	Cat# R3136T
cOmplete^™^, Mini, EDTA-free Protease Inhibitor Cocktail	Sigma Aldritch	Cat# 11836170001
Agencourt AMPure XP	Beckman Coulter	Cat# A63881
Anti-FLAG^®^ M2 Magnetic Beads	Sigma Aldritch	Cat# M8823–1ML
Pierce^™^ Anti-HA Magnetic Beads	Thermo Fisher Scientific	Cat# 88836
ALFA Selector ST	NanoTag	Cat# N1516-L
Glutathione Agarose Beads	MCLAB	Cat# GAB-300
PreScission Protease	GE Healthcare	Cat# 27–0843-01
ProLong^™^ Gold Antifade Mountant with DAPI	Thermo Fisher Scientific	Cat# P36941
Bafilomycin A1	Cell Signaling Technology	Cat# 54645S
MG-132	Selleck Chemicals	Cat# S2619
TAK-243 (MLN7243)	Selleck Chemicals	Cat# S8341
Heclin	Selleck Chemicals	Cat# E1216
Recombinant Human IFN-β	Peprotech	Cat# 300–02BC
Recombinant Human IFN-γ	Peprotech	Cat# 300–02
Poly(dA:dT)	Invivogen	Cat# tlrl-patn-1
5-FAM labeled peptide (MC162R_469–489_: GHLPPPPPYCPVPPPYSDNTR)	GenScript	N/A
5-FAM labeled peptide (MC162R-PA_469–489_: GHLPPAAAACPVAAAASDNTR)	GenScript	N/A

Critical commercial assays		

GeneJET Genomic DNA Purification Kit	Thermo Fisher Scientific	Cat# K0721
NucleoSpin Gel and PCR Clean-up Kit	Macherey-Nagel	Cat# 740609
Gateway^™^ LR Clonase^™^ II Enzyme mix	Thermo Fisher Scientific	Cat# 11791100
Gateway^™^ BP Clonase^™^ II Enzyme mix	Thermo Fisher Scientific	Cat# 11789100
NEBuilder^®^ HiFi DNA Assembly Master Mix	New England Biolabs	Cat# E2621L
Q5^®^ High-Fidelity 2X Master Mix	New England Biolabs	Cat# M0492L
iScript cDNA Synthesis Kit	BioRad	Cat# 1708891
RNeasy Plus RNA Extraction Kit	Qiagen	Cat# 74136
Platinum SYBR Green qPCR Supermix	Invitrogen	Cat# 11733–046
CellTrace^™^ CFSE Cell Proliferation Kit, for flow cytometry	Invitrogen	Cat# C34554
CellTrace^™^ Violet Cell Proliferation Kit, for flow cytometry	Invitrogen	Cat# C34557

Deposited data		

MC162R-BirA* proteomics data (Figure 5B)	This study	PRIDE: PXD077896
MC162R whole cell proteomics (Figure S5F)	This study	PRIDE: PXD077768
YLDV 151R IP-MS proteomics (Figure S7J)	This study	PRIDE: PXD077910

Experimental models: Cell lines		

HEK-293T	ATCC	Cat# CRL-3216; RRID:CVCL_0063
A375	ATCC	Cat# CRL-1619; RRID:CVCL_0132
Immortalized HCEC	Roig et al.^74^	N/A
hTERT-RPE1	ATCC	Cat# CRL-4000; RRID:CVCL_4388
THP-1	ATCC	Cat# TIB-202; RRID:CVCL_0006

Oligonucleotides		

See Table S8	N/A	N/A

Recombinant DNA		

Viral ORFeome Library	This study	N/A
pFuji101	This study	N/A
pHAGE-TRE-DEST-Pme1-puro	Sack et al.^21^	N/A
pINDUCER20	Meerbrey et al.^75^	N/A
psPAX2	Addgene	Cat# 12260; RRID:Addgene_12260
pMD2.G	Addgene	Cat# 12259; RRID:Addgene_12259
pHAGE-TREx-DEST-PGK-NAT	Dezfulian et al.^76^	N/A
pHAGE-EF1-DEST-PGK-NAT	Dezfulian et al.^76^	N/A
pHAGE-TRE-3xHA-DEST	This study	N/A
pHAGE-TRE-BirA*-DEST-Neo	This study	N/A
LentiCRISPR v2	Addgene	Cat# 52961; RRID:Addgene_52961
LentiCRISPR v2 NAT	This study	N/A
LentiCRISPR v2 EGFP	This study	N/A
IRF9 Ultimate ORF Collection Entry Clone (Clone ID: IOH28745)	Thermo Fisher Scientific	N/A
MC162R vORF Entry Clone (Fragment ID: Q98328.1)	This study	N/A
YLDV 151R vORF Entry Clone (Fragment ID: Q9DGU2.1)	This study	N/A
YMTV 151R vORF Entry Clone (Fragment ID: Q6TUM3.1)	This study	N/A
VACV C16L vORF Entry Clone	This study	N/A
VACV C10L vORF Entry Clone (Fragment ID: P21043.0.1)	This study	N/A
VACV C4L vORF Entry Clone (Fragment ID: P21038.0.1)	This study	N/A
Monkeypox virus D13L vORF Entry Clone (Fragment ID: Q8V563.1)	This study	N/A
EBV BRRF1 vORF Entry Clone (Fragment ID: P03207.0.1)	This study	N/A
IAV NP vORF Entry Clone (Fragment ID: P15682.0.1)	This study	N/A
MC145R vORF Entry Clone (Fragment ID: Q98311.1)	This study	N/A
YOGV RdRp_1 vORF Entry Clone (Fragment ID: YP_009246486.1.1)	This study	N/A
ALHV-1 VG36 vORF Entry Clone (Fragment ID: O36385.0.1)	This study	N/A
PSHV-1 OBP vORF Entry Clone (Fragment ID: Q6UDH3.0.1)	This study	N/A
LNV VP2 vORF Entry Clone (Fragment ID: YP_460027.1.3)	This study	N/A
HAdV E1B vORF Entry Clone (Fragment ID: B5SNR2.1)	This study	N/A
EqHV VG59 vORF Entry Clone (Fragment ID: Q66661.0.1)	This study	N/A
MuHV-1 IE1 vORF Entry Clone (Fragment ID: P11210.0.1)	This study	N/A
HCMV US28 vORF Entry Clone (Fragment ID: B9EKY5.1)	This study	N/A
AvPMV M vORF Entry Clone (Fragment ID: YP_009094170.1.1)	This study	N/A
BUNV NSs vORF Entry Clone (Fragment ID: NP_047214.1.1)	This study	N/A
VZV ORF41 vORF Entry Clone (Fragment ID: Q4JQT4.1)	This study	N/A
YLDV 132R vORF Entry Clone (Fragment ID: Q9DHI1.1)	This study	N/A
Monkeypox virus A9R vORF Entry Clone (Fragment ID: Q8V4W7.1)	This study	N/A
CeHV-1 US2 vORF Entry Clone (Fragment ID: Q5EGY0.1)	This study	N/A
ABLV P vORF Entry Clone (Fragment ID: Q9QSP3.0.1)	This study	N/A
RABV P vORF Entry Clone (Fragment ID: P69479.0.1)	This study	N/A
ARAV P vORF Entry Clone (Fragment ID: YP_007641393.1.1)	This study	N/A
MOKV P vORF Entry Clone (Fragment ID: YP_142351.1.1)	This study	N/A
EBLV2 P vORF Entry Clone (Fragment ID: YP_001285394.2.1)	This study	N/A
IRKV P vORF Entry Clone (Fragment ID: YP_007641398.1.1)	This study	N/A
KHUV P vORF Entry Clone (Fragment ID: YP_009094328.1.1)	This study	N/A
GBLV P vORF Entry Clone (Fragment ID: YP_009325515.1.1)	This study	N/A
LBV P vORF Entry Clone (Fragment ID: YP_007641388.1.1)	This study	N/A
pGEX-murine ITCH (aa143–864)	Chen et al.^77^	N/A
pHAGE-EF1a-HLA-A2	Kula et al.^78^	N/A

Software and algorithms		

Bowtie 2	Langmead and Salzberg^79^; Langmead et al.^80^	http://bowtie-bio.sourceforge.net/index.shtml
Cutadapt	Martin^81^	https://cutadapt.readthedocs.io/en/stable/index.html
Mmseq2	Steinegger and Soding^55^	https://github.com/soedinglab/mmseqs2, RRID:SCR_026553
MAGeCK	Li et al.^12^	https://sourceforge.net/projects/mageck/, RRID:SCR_025016
Python v3.7	Python Software Foundation	https://www.python.org
R v4.4.2	R Project	https://www.r-project.org/
AlphaFold Server	Abramson et al.^82^	https://deepmind.google/technologies/alphafold/alphafold-server/, RRID:SCR_025885
FoldSeek Search Server	Van Kempen et al.^61^	https://search.foldseek.com/search
PDBePISA	Krissinel and Henrick^71^	https://www.ebi.ac.uk/pdbe/pisa/pistart.html
UCSF ChimeraX 1.9	Pettersen et al.^83^; Goodard et al.^84^	https://www.cgl.ucsf.edu/chimerax/, RRID:SCR_015872
FlowJo	FlowJo	https://www.flowjo.com/
FIJI	Schindelin et al.^85^	https://fiji.sc/, RRID:SCR_002285
CellProfiler	Stirling et al.^86^	http://cellprofiler.org
BioRender	BioRender	https://www.biorender.com
GraphPad Prism version 10	GraphPad Prism	https://www.graphpad.com, RRID:SCR_002798

Other		

CytoFLEX LX Flow Cytometer	Beckman Coulter	Cat# C40321
MA900 Multi-Application Cell Sorter	Sony	Cat# LE-MA900FP

## Highlights

- A viral ORFeome enables screening of ~10,000 distinct viral proteins
- Virome screens identify >700 proliferation, MHC class I, and IFN signaling regulators
- MC162R degrades MHC class I via redundant recruitment of NEDD4-like E3 ligases
- YLDV 151R binds IRF9 to suppress IFN-β signaling, diverging from poxvirus homologs

## References

[1] Wylie KM, Weinstock GM, and Storch GA (2012). Emerging view of the human virome. Transl. Res. 160, 283–290. 10.1016/j.trsl.2012.03.006. 22683423 PMC3701101

[2] Ng TL, Olson EJ, Yoo TY, Weiss HS, Koide Y, Koch PD, Rollins NJ, Mach P, Meisinger T, Bricken T, (2022). High-Content Screening and Computational Prediction Reveal Viral Genes That Suppress the Innate Immune Response. mSystems 7. e01466–21. 10.1128/msystems.01466-21. PMC9040872 35319251

[3] Sen J, Liu X, Roller R, and Knipe DM (2013). Herpes simplex virus US3 tegument protein inhibits Toll-like receptor 2 signaling at or before TRAF6 ubiquitination. Virology 439, 65–73. 10.1016/j.virol.2013.01.026. 23478027 PMC3810314

[4] Calderwood MA, Venkatesan K, Xing L, Chase MR, Vazquez A, Holthaus AM, Ewence AE, Li N, Hirozane-Kishikawa T, Hill DE, (2007). Epstein–Barr virus and virus human protein interaction maps. Proc. Natl. Acad. Sci. USA 104, 7606–7611. 10.1073/pnas.0702332104. 17446270 PMC1863443

[5] Davis ZH, Verschueren E, Jang GM, Kleffman K, Johnson JR, Park J, Von Dollen J, Maher MC, Johnson T, Newton W, (2015). Global Mapping of Herpesvirus-Host Protein Complexes Reveals a Transcription Strategy for Late Genes. Mol. Cell 57, 349–360. 10.1016/j.molcel.2014.11.026. 25544563 PMC4305015

[6] Tscharke DC, Karupiah G, Zhou J, Palmore T, Irvine KR, Haeryfar SMM, Williams S, Sidney J, Sette A, Bennink JR, (2005). Identification of poxvirus CD8+ T cell determinants to enable rational design and characterization of smallpox vaccines. J. Exp. Med. 201, 95–104. 10.1084/jem.20041912. 15623576 PMC2212779

[7] Suzek BE, Huang H, McGarvey P, Mazumder R, and Wu CH (2007). UniRef: comprehensive and non-redundant UniProt reference clusters. Bioinformatics 23, 1282–1288. 10.1093/bioinformatics/btm098. 17379688

[8] Stern-Ginossar N, Weisburd B, Michalski A, Le VTK, Hein MY, Huang S-X, Ma M, Shen B, Qian S-B, Hengel H, (2012). Decoding human cytomegalovirus. Science 338, 1088–1093. 10.1126/science.1227919. 23180859 PMC3817102

[9] Vita R, Blazeska N, Marrama D, , ; IEDB; Curation Team Members, Duesing S, Bennett J, Greenbaum J, De Almeida Mendes M, Mahita J, Wheeler DK (2025). The Immune Epitope Database (IEDB): 2024 update. Nucleic Acids Res. 53, D436–D443. 10.1093/nar/gkae1092. 39558162 PMC11701597

[10] Zhu J, Larman HB, Gao G, Somwar R, Zhang Z, Laserson U, Ciccia A, Pavlova N, Church G, Zhang W, (2013). Protein interaction discovery using parallel analysis of translated ORFs (PLATO). Nat. Biotechnol. 31, 331–334. 10.1038/nbt.2539. 23503679 PMC4110636

[11] Larman HB, Liang AC, Elledge SJ, and Zhu J (2014). Discovery of protein interactions using parallel analysis of translated ORFs (PLATO). Nat. Protoc. 9, 90–103. 10.1038/nprot.2013.167. 24336473 PMC4129458

[12] Li W, Xu H, Xiao T, Cong L, Love MI, Zhang F, Irizarry RA, Liu JS, Brown M, and Liu XS (2014). MAGeCK enables robust identification of essential genes from genome-scale CRISPR/Cas9 knockout screens. Genome Biol. 15, 554. 10.1186/s13059-014-0554-4. 25476604 PMC4290824

[13] Anand SK, and Tikoo SK (2013). Viruses as Modulators of Mitochondrial Functions. Adv. Virol. 2013, 1–17. 10.1155/2013/738794. PMC3821892 24260034

[14] Li S, Kong L, and Yu X (2015). The expanding roles of endoplasmic reticulum stress in virus replication and pathogenesis. Crit. Rev. Microbiol. 41, 150–164. 10.3109/1040841X.2013.813899. 25168431 PMC7113905

[15] Hrecka K, Gierszewska M, Srivastava S, Kozaczkiewicz L, Swanson SK, Florens L, Washburn MP, and Skowronski J (2007). Lentiviral Vpr usurps Cul4–DDB1[VprBP] E3 ubiquitin ligase to modulate cell cycle. Proc. Natl. Acad. Sci. USA 104, 11778–11783. 10.1073/pnas.0702102104. 17609381 PMC1906728

[16] Tan L, Ehrlich E, and Yu X-F (2007). DDB1 and Cul4A Are Required for Human Immunodeficiency Virus Type 1 Vpr-Induced G 2 Arrest. J. Virol. 81, 10822–10830. 10.1128/JVI.01380-07. 17626091 PMC2045451

[17] Padilla-Noriega L, Paniagua O, and Guzmán-León S (2002). Rotavirus Protein NSP3 Shuts Off Host Cell Protein Synthesis. Virology 298, 1–7. 10.1006/viro.2002.1477. 12093167

[18] Piron M (1998). Rotavirus RNA-binding protein NSP3 interacts with eIF4GI and evicts the poly(A) binding protein from eIF4F. EMBO J. 17, 5811–5821. 10.1093/emboj/17.19.5811. 9755181 PMC1170909

[19] Kong K, Kumar M, Taruishi M, and Javier RT (2015). Adenovirus E4-ORF1 Dysregulates Epidermal Growth Factor and Insulin/Insulin-Like Growth Factor Receptors To Mediate Constitutive Myc Expression. J. Virol. 89, 10774–10785. 10.1128/JVI.01463-15. 26269183 PMC4621122

[20] McLaughlin-Drubin ME, and Münger K (2009). The human papillomavirus E7 oncoprotein. Virology 384, 335–344. 10.1016/j.virol.2008.10.006. 19007963 PMC2661820

[21] Sack LM, Davoli T, Li MZ, Li Y, Xu Q, Naxerova K, Wooten EC, Bernardi RJ, Martin TD, Chen T, (2018). Profound Tissue Specificity in Proliferation Control Underlies Cancer Drivers and Aneuploidy Patterns. Cell 173, 499–514.e23. 10.1016/j.cell.2018.02.037. 29576454 PMC6643283

[22] Kwong AD, and Frenkel N (1987). Herpes simplex virus-infected cells contain a function(s) that destabilizes both host and viral mRNAs. Proc. Natl. Acad. Sci. USA 84, 1926–1930. 10.1073/pnas.84.7.1926. 3031658 PMC304554

[23] Santerre M, Chatila W, Wang Y, Mukerjee R, and Sawaya BE (2019). HIV-1 Nef promotes cell proliferation and microRNA dysregulation in lung cells. Cell Cycle 18, 130–142. 10.1080/15384101.2018.1557487. 30563405 PMC6343720

[24] Hansen TH, and Bouvier M (2009). MHC class I antigen presentation: learning from viral evasion strategies. Nat. Rev. Immunol. 9, 503–513. 10.1038/nri2575. 19498380

[25] Hewitt EW (2003). The MHC class I antigen presentation pathway: strategies for viral immune evasion. Immunology 110, 163–169. 10.1046/j.1365-2567.2003.01738.x. 14511229 PMC1783040

[26] Yewdell JW, and Hill AB (2002). Viral interference with antigen presentation. Nat. Immunol. 3, 1019–1025. 10.1038/ni1102-1019. 12407410

[27] Jugovic P, Hill AM, Tomazin R, Ploegh H, and Johnson DC (1998). Inhibition of major histocompatibility complex class I antigen presentation in pig and primate cells by herpes simplex virus type 1 and 2 ICP47. J. Virol. 72, 5076–5084. 10.1128/JVI.72.6.5076-5084.1998. 9573278 PMC110071

[28] Williams M, Roeth JF, Kasper MR, Fleis RI, Przybycin CG, and Collins KL (2002). Direct Binding of Human Immunodeficiency Virus Type 1 Nef to the Major Histocompatibility Complex Class I (MHC-I) Cytoplasmic Tail Disrupts MHC-I Trafficking. J. Virol. 76, 12173–12184. 10.1128/JVI.76.23.12173-12184.2002. 12414957 PMC136906

[29] Tomazin R, Boname J, Hegde NR, Lewinsohn DM, Altschuler Y, Jones TR, Cresswell P, Nelson JA, Riddell SR, and Johnson DC (1999). Cytomegalovirus US2 destroys two components of the MHC class II pathway, preventing recognition by CD4+ T cells. Nat. Med. 5, 1039–1043. 10.1038/12478. 10470081

[30] Hegde NR, and Johnson DC (2003). Human Cytomegalovirus US2 Causes Similar Effects on Both Major Histocompatibility Complex Class I and II Proteins in Epithelial and Glial Cells. J. Virol. 77, 9287–9294. 10.1128/JVI.77.17.9287-9294.2003. 12915544 PMC187418

[31] Ishido S, Wang C, Lee B-S, Cohen GB, and Jung JU (2000). Down-regulation of Major Histocompatibility Complex Class I Molecules by Kaposi’s Sarcoma-Associated Herpesvirus K3 and K5 Proteins. J. Virol. 74, 5300–5309. 10.1128/JVI.74.11.5300-5309.2000. 10799607 PMC110885

[32] Li W, and Godzik A (2006). Cd-hit: a fast program for clustering and comparing large sets of protein or nucleotide sequences. Bioinformatics 22, 1658–1659. 10.1093/bioinformatics/btl158. 16731699

[33] Gottlieb SL, and Myskowski PL (1994). MOLLUSCUM CONTAGIOSUM. Int. J. Dermatol. 33, 453–461. 10.1111/j.1365-4362.1994.tb02853.x. 7928025

[34] Mirkina I, Hadzijusufovic E, Krepler C, Mikula M, Mechtcheriakova D, Strommer S, Stella A, Jensen-Jarolim E, Höller C, Wacheck V, (2014). Phenotyping of human melanoma cells reveals a unique composition of receptor targets and a subpopulation co-expressing ErbB4, EPO-R and NGF-R. PLoS One 9, e84417. 10.1371/journal.pone.0084417. PMC3906015 24489649

[35] Sung E, Ko M, Won J, Jo Y, Park E, Kim H, Choi E, Jung U, Jeon J, Kim Y, (2022). LAG-3xPD-L1 bispecific antibody potentiates antitumor responses of T cells through dendritic cell activation. Mol. Ther. 30, 2800–2816. 10.1016/j.ymthe.2022.05.003. 35526096 PMC9372323

[36] Hyer ML, Milhollen MA, Ciavarri J, Fleming P, Traore T, Sappal D, Huck J, Shi J, Gavin J, Brownell J, (2018). A small-molecule inhibitor of the ubiquitin activating enzyme for cancer treatment. Nat. Med. 24, 186–193. 10.1038/nm.4474. 29334375

[37] Xu Q, Farah M, Webster JM, and Wojcikiewicz RJH (2004). Bortezomib rapidly suppresses ubiquitin thiolesterification to ubiquitin-conjugating enzymes and inhibits ubiquitination of histones and type I inositol 1,4,5-trisphosphate receptor. Mol. Cancer Ther. 3, 1263–1269. 10.1158/1535-7163.1263.3.10. 15486193

[38] Coscoy L, and Ganem D (2000). Kaposi’s sarcoma-associated herpesvirus encodes two proteins that block cell surface display of MHC class I chains by enhancing their endocytosis. Proc. Natl. Acad. Sci. USA 97, 8051–8056. 10.1073/pnas.140129797. 10859362 PMC16668

[39] Van Den Boomen DJH, and Lehner PJ (2015). Identifying the ERAD ubiquitin E3 ligases for viral and cellular targeting of MHC class I. Mol. Immunol. 68, 106–111. 10.1016/j.molimm.2015.07.005. 26210183 PMC4678111

[40] Zhang Z, Sie B, Chang A, Leng Y, Nardone C, Timms RT, and Elledge SJ (2023). Elucidation of E3 ubiquitin ligase specificity through proteome-wide internal degron mapping. Mol. Cell 83, 3377–3392.e6. 10.1016/j.molcel.2023.08.022. 37738965 PMC10594193

[41] Lu Q, Hope LW, Brasch M, Reinhard C, and Cohen SN (2003). TSG101 interaction with HRS mediates endosomal trafficking and receptor down-regulation. Proc. Natl. Acad. Sci. USA 100, 7626–7631. 10.1073/pnas.0932599100. 12802020 PMC164637

[42] Raiborg C, Bache KG, Gillooly DJ, Madshus IH, Stang E, and Stenmark H (2002). Hrs sorts ubiquitinated proteins into clathrin-coated microdomains of early endosomes. Nat. Cell Biol. 4, 394–398. 10.1038/ncb791. 11988743

[43] Katzmann DJ, Babst M, and Emr SD (2001). Ubiquitin-Dependent Sorting into the Multivesicular Body Pathway Requires the Function of a Conserved Endosomal Protein Sorting Complex, ESCRT-I. Cell 106, 145–155. 10.1016/S0092-8674(01)00434-2. 11511343

[44] Rotin D, and Kumar S (2009). Physiological functions of the HECT family of ubiquitin ligases. Nat. Rev. Mol. Cell Biol. 10, 398–409. 10.1038/nrm2690. 19436320

[45] Li P, Meng Y, Wang L, and Di L (2019). BioID: A Proximity-Dependent Labeling Approach in Proteomics Study. In Functional Proteomics Methods in Molecular Biology, Wang X and Kuruc M, eds. (Springer), pp. 143–151. 10.1007/978-1-4939-8814-3_10. 30276738

[46] Mund T, Lewis MJ, Maslen S, and Pelham HR (2014). Peptide and small molecule inhibitors of HECT-type ubiquitin ligases. Proc. Natl. Acad. Sci. USA 111, 16736–16741. 10.1073/pnas.1412152111. 25385595 PMC4250122

[47] Sadasivan B, Lehner PJ, Ortmann B, Spies T, and Cresswell P (1996). Roles for calreticulin and a novel glycoprotein, tapasin, in the interaction of MHC class I molecules with TAP. Immunity 5, 103–114. 10.1016/s1074-7613(00)80487-2. 8769474

[48] Chao MP, Jaiswal S, Weissman-Tsukamoto R, Alizadeh AA, Gentles AJ, Volkmer J, Weiskopf K, Willingham SB, Raveh T, Park CY, (2010). Calreticulin is the dominant pro-phagocytic signal on multiple human cancers and is counterbalanced by CD47. Sci. Transl. Med. 2, 63ra94. 10.1126/scitranslmed.3001375. PMC4126904 21178137

[49] Vidy A, Chelbi-Alix M, and Blondel D (2005). Rabies Virus P Protein Interacts with STAT1 and Inhibits Interferon Signal Transduction Pathways. J. Virol. 79, 14411–14420. 10.1128/JVI.79.22.14411-14420.2005. 16254375 PMC1280226

[50] Vidy A, El Bougrini J, Chelbi-Alix MK, and Blondel D (2007). The Nucleocytoplasmic Rabies Virus P Protein Counteracts Interferon Signaling by Inhibiting both Nuclear Accumulation and DNA Binding of STAT1. J. Virol. 81, 4255–4263. 10.1128/JVI.01930-06. 17287281 PMC1866157

[51] Fensterl V, Chattopadhyay S, and Sen GC (2015). No Love Lost Between Viruses and Interferons. Annu. Rev. Virol. 2, 549–572. 10.1146/annurev-virology-100114-055249. 26958928 PMC9549753

[52] García-Sastre A (2017). Ten Strategies of Interferon Evasion by Viruses. Cell Host Microbe 22, 176–184. 10.1016/j.chom.2017.07.012. 28799903 PMC5576560

[53] Shaw ML, García-Sastre A, Palese P, and Basler CF (2004). Nipah Virus V and W Proteins Have a Common STAT1-Binding Domain yet Inhibit STAT1 Activation from the Cytoplasmic and Nuclear Compartments, Respectively. J. Virol. 78, 5633–5641. 10.1128/JVI.78.11.5633-5641.2004. 15140960 PMC415790

[54] Garcin D, Latorre P, and Kolakofsky D (1999). Sendai Virus C Proteins Counteract the Interferon-Mediated Induction of an Antiviral State. J. Virol. 73, 6559–6565. 10.1128/JVI.73.8.6559-6565.1999. 10400752 PMC112739

[55] Steinegger M, and Söding J (2017). MMseqs2 enables sensitive protein sequence searching for the analysis of massive data sets. Nat. Biotechnol. 35, 1026–1028. 10.1038/nbt.3988. 29035372

[56] Downie AW, Taylor-Robinson CH, Caunt AE, Nelson GS, Manson-Bahr PEC, and Matthews TCH (1971). Tanapox: A New Disease Caused by a Pox Virus. BMJ 1, 363–368. 10.1136/bmj.1.5745.363. 5541925 PMC1795031

[57] Birkhead M, Grayson W, Grobbelaar A, Msimang V, Moolla N, Mathee A, Blumberg L, Marshall T, Morobadi D, Popara M, (2023). Tanapox, South Africa, 2022. Emerg. Infect. Dis. 29, 1206–1209. 10.3201/eid2906.230326. 37022936 PMC10202874

[58] Dhar AD, Werchniak AE, Li Y, Brennick JB, Goldsmith CS, Kline R, Damon I, and Klaus SN (2004). Tanapox Infection in a College Student. N. Engl. J. Med. 350, 361–366. 10.1056/NEJMoa031467. 14736928

[59] Lee H-J, Essani K, and Smith GL (2001). The Genome Sequence of Yaba-like Disease Virus, a Yatapoxvirus. Virology 281, 170–192. 10.1006/viro.2000.0761. 11277691

[60] Brunetti CR, Amano H, Ueda Y, Qin J, Miyamura T, Suzuki T, Li X, Barrett JW, and McFadden G (2003). Complete genomic sequence and comparative analysis of the tumorigenic poxvirus Yaba monkey tumor virus. J. Virol. 77, 13335–13347. 10.1128/jvi.77.24.13335-13347.2003. 14645589 PMC296094

[61] Van Kempen M, Kim SS, Tumescheit C, Mirdita M, Lee J, Gilchrist CLM, Söding J, and Steinegger M (2023). Fast and accurate protein structure search with Foldseek. Nat. Biotechnol. 42, 243–246. 10.1038/s41587-023-01773-0. 37156916 PMC10869269

[62] Fahy AS, Clark RH, Glyde EF, and Smith GL (2008). Vaccinia virus protein C16 acts intracellularly to modulate the host response and promote virulence. J. Gen. Virol. 89, 2377–2387. 10.1099/vir.0.2008/004895-0. 18796705 PMC2885005

[63] Scutts SR, Ember SW, Ren H, Ye C, Lovejoy CA, Mazzon M, Veyer DL, Sumner RP, and Smith GL (2018). DNA-PK Is Targeted by Multiple Vaccinia Virus Proteins to Inhibit DNA Sensing. Cell Rep. 25, 1953–1965.e4. 10.1016/j.celrep.2018.10.034. 30428360 PMC6250978

[64] Rivera-Calzada A, Arribas-Bosacoma R, Ruiz-Ramos A, Escudero-Bravo P, Boskovic J, Fernandez-Leiro R, Oliver AW, Pearl LH, and Llorca O (2022). Structural basis for the inactivation of cytosolic DNA sensing by the vaccinia virus. Nat. Commun. 13, 7062. 10.1038/s41467-022-34843-z. 36400800 PMC9674614

[65] Peters NE, Ferguson BJ, Mazzon M, Fahy AS, Krysztofinska E, Arribas-Bosacoma R, Pearl LH, Ren H, and Smith GL (2013). A Mechanism for the Inhibition of DNA-PK-Mediated DNA Sensing by a Virus. PLoS Pathog. 9, e1003649. 10.1371/journal.ppat.1003649. PMC3789764 24098118

[66] Schneider WM, Chevillotte MD, and Rice CM (2014). Interferon-Stimulated Genes: A Complex Web of Host Defenses. Annu. Rev. Immunol. 32, 513–545. 10.1146/annurev-immunol-032713-120231. 24555472 PMC4313732

[67] Rengachari S, Groiss S, Devos JM, Caron E, Grandvaux N, and Panne D (2018). Structural basis of STAT2 recognition by IRF9 reveals molecular insights into ISGF3 function. Proc. Natl. Acad. Sci. USA 115, E601–E609. 10.1073/pnas.1718426115. 29317535 PMC5789952

[68] Platanitis E, Demiroz D, Schneller A, Fischer K, Capelle C, Hartl M, Gossenreiter T, Müller M, Novatchkova M, and Decker T (2019). A molecular switch from STAT2-IRF9 to ISGF3 underlies interferon-induced gene transcription. Nat. Commun. 10, 2921. 10.1038/s41467-019-10970-y. 31266943 PMC6606597

[69] Schoggins JW, and Rice CM (2011). Interferon-stimulated genes and their antiviral effector functions. Curr. Opin. Virol. 1, 519–525. 10.1016/j.coviro.2011.10.008. 22328912 PMC3274382

[70] Fu XY, Kessler DS, Veals SA, Levy DE, and Darnell JE (1990). ISGF3, the transcriptional activator induced by interferon alpha, consists of multiple interacting polypeptide chains. Proc. Natl. Acad. Sci. USA 87, 8555–8559. 10.1073/pnas.87.21.8555. 2236065 PMC54995

[71] Krissinel E, and Henrick K (2007). Inference of Macromolecular Assemblies from Crystalline State. J. Mol. Biol. 372, 774–797. 10.1016/j.jmb.2007.05.022. 17681537

[72] Brass AL, Dykxhoorn DM, Benita Y, Yan N, Engelman A, Xavier RJ, Lieberman J, and Elledge SJ (2008). Identification of host proteins required for HIV infection through a functional genomic screen. Science 319, 921–926. 10.1126/science.1152725. 18187620

[73] Gigante CM, Gao J, Tang S, McCollum AM, Wilkins K, Reynolds MG, Davidson W, McLaughlin J, Olson VA, and Li Y (2019). Genome of Alaskapox Virus, a Novel Orthopoxvirus Isolated from Alaska. Viruses 11, 708. 10.3390/v11080708. 31375015 PMC6723315

[74] Roig AI, Eskiocak U, Hight SK, Kim SB, Delgado O, Souza RF, Spechler SJ, Wright WE, and Shay JW (2010). Immortalized epithelial cells derived from human colon biopsies express stem cell markers and differentiate in vitro. Gastroenterology 138, 1012–1021.e5. 10.1053/j.gastro.2009.11.052. 19962984

[75] Meerbrey KL, Hu G, Kessler JD, Roarty K, Li MZ, Fang JE, Herschkowitz JI, Burrows AE, Ciccia A, Sun T, (2011). The pINDUCER lentiviral toolkit for inducible RNA interference in vitro and in vivo. Proc. Natl. Acad. Sci. USA 108, 3665–3670. 10.1073/pnas.1019736108. 21307310 PMC3048138

[76] Dezfulian MH, Kula T, Pranzatelli T, Kamitaki N, Meng Q, Khatri B, Perez P, Xu Q, Chang A, Kohlgruber AC, (2023). TScan-II: A genome-scale platform for the de novo identification of CD4+ T cell epitopes. Cell 186, 5569–5586.e21. 10.1016/j.cell.2023.10.024. 38016469 PMC10841602

[77] Chen Z, Jiang H, Xu W, Li X, Dempsey DR, Zhang X, Devreotes P, Wolberger C, Amzel LM, Gabelli SB, (2017). A Tunable Brake for HECT Ubiquitin Ligases. Mol. Cell 66, 345–357.e6. 10.1016/j.molcel.2017.03.020. 28475870 PMC5489419

[78] Kula T, Dezfulian MH, Wang CI, Abdelfattah NS, Hartman ZC, Wucherpfennig KW, Lyerly HK, and Elledge SJ (2019). T-Scan: A Genome-wide Method for the Systematic Discovery of T Cell Epitopes. Cell 178, 1016–1028.e13. 10.1016/j.cell.2019.07.009. 31398327 PMC6939866

[79] Langmead B, and Salzberg SL (2012). Fast gapped-read alignment with Bowtie 2. Nat. Methods 9, 357–359. 10.1038/nmeth.1923. 22388286 PMC3322381

[80] Langmead B, Trapnell C, Pop M, and Salzberg SL (2009). Ultrafast and memory-efficient alignment of short DNA sequences to the human genome. Genome Biol. 10, R25. 10.1186/gb-2009-10-3-r25. 19261174 PMC2690996

[81] Martin M (2011). Cutadapt removes adapter sequences from high-throughput sequencing reads. EMBnet J. 17, 10. 10.14806/ej.17.1.200.

[82] Abramson J, Adler J, Dunger J, Evans R, Green T, Pritzel A, Ronneberger O, Willmore L, Ballard AJ, Bambrick J, (2024). Accurate structure prediction of biomolecular interactions with AlphaFold 3. Nature 630, 493–500. 10.1038/s41586-024-07487-w. 38718835 PMC11168924

[83] Pettersen EF, Goddard TD, Huang CC, Meng EC, Couch GS, Croll TI, Morris JH, and Ferrin TE (2021). UCSF ChimeraX: Structure visualization for researchers, educators, and developers. Protein Sci. 30, 70–82. 10.1002/pro.3943. 32881101 PMC7737788

[84] Goddard TD, Huang CC, Meng EC, Pettersen EF, Couch GS, Morris JH, and Ferrin TE (2018). UCSF ChimeraX: Meeting modern challenges in visualization and analysis. Protein Sci. 27, 14–25. 10.1002/pro.3235. 28710774 PMC5734306

[85] Schindelin J, Arganda-Carreras I, Frise E, Kaynig V, Longair M, Pietzsch T, Preibisch S, Rueden C, Saalfeld S, Schmid B, (2012). Fiji: an open-source platform for biological-image analysis. Nat. Methods 9, 676–682. 10.1038/nmeth.2019. 22743772 PMC3855844

[86] Stirling DR, Swain-Bowden MJ, Lucas AM, Carpenter AE, Cimini BA, and Goodman A (2021). CellProfiler 4: improvements in speed, utility and usability. BMC Bioinform. 22, 433. 10.1186/s12859-021-04344-9. PMC8431850 34507520

[87] Timms RT, Zhang Z, Rhee DY, Harper JW, Koren I, and Elledge SJ (2019). A glycine-specific N-degron pathway mediates the quality control of protein N -myristoylation. Science 365, eaaw4912. 10.1126/science.aaw4912. PMC7090375 31273098

[88] Nardone C, Palanski BA, Scott DC, Timms RT, Barber KW, Gu X, Mao A, Leng Y, Watson EV, Schulman BA, (2023). A central role for regulated protein stability in the control of TFE3 and MITF by nutrients. Mol. Cell 83, 57–73.e9. 10.1016/j.molcel.2022.12.013. 36608670 PMC9908011

[89] Gu X, Nardone C, Kamitaki N, Mao A, Elledge SJ, and Greenberg ME (2023). The midnolin-proteasome pathway catches proteins for ubiquitination-independent degradation. Science 381, eadh5021. 10.1126/science.adh5021. PMC10617673 37616343

[90] Navarrete-Perea J, Yu Q, Gygi SP, and Paulo JA (2018). Streamlined Tandem Mass Tag (SL-TMT) Protocol: An Efficient Strategy for Quantitative (Phospho)proteome Profiling Using Tandem Mass Tag-Synchronous Precursor Selection-MS3. J. Proteome Res. 17, 2226–2236. 10.1021/acs.jproteome.8b00217. 29734811 PMC5994137

