# Supplementary material for: A viral ORFeome library for systems-level genetic dissection of host-pathogen interactions
Source: Cell. Author manuscript; Available in PMC 2026 Aug 2. (PMC13429030; doi:10.1016/j.cell.2026.05.024)
Supplement: MMC1 [file NIHMS2180536-supplement-MMC1.pdf]

Cell, Volume *189*

Supplemental information

A viral ORFeome library

for systems-level genetic dissection

# of host-pathogen interactions

Eric Fujimura, Colin N. O'Leary, Mamie Z. Li, Rachel A. Roberts, Caleb R. Glassman, Joao A. Paulo, Hanjie Jiang, Nouran S. Abdelfattah, Eric C. Wooten, Zachary Mirman, J. Wade Harper, Philip A. Cole, and Stephen J. Elledge

Figure S1

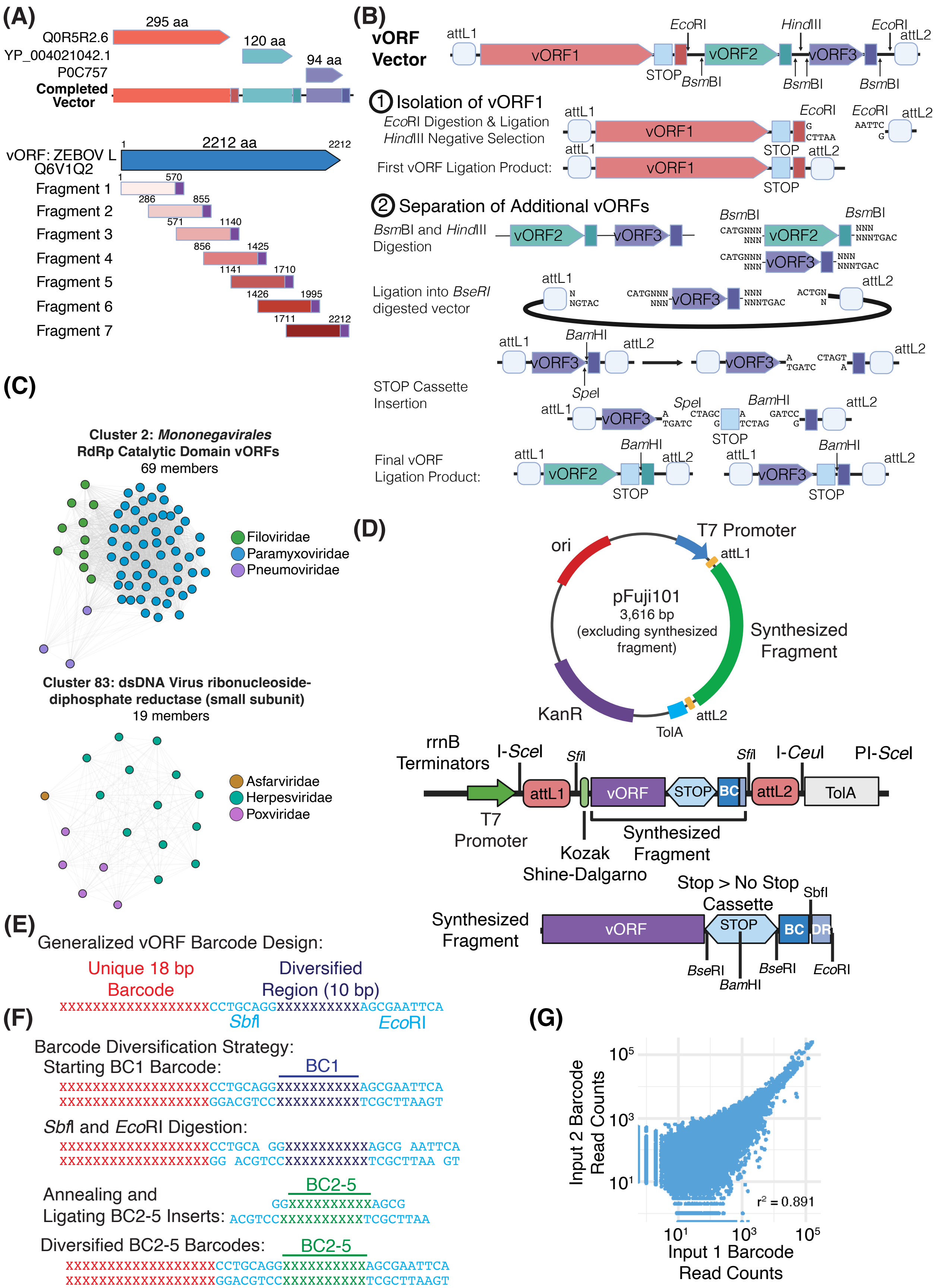

(A)
295 aa
Q0R5R2.6
120 aa
YP_004021042.1
94 aa
P0C757
Completed Vector
vORF: ZEBOV L Q6V1Q2
2212 aa
Fragment 1
Fragment 2
Fragment 3
Fragment 4
Fragment 5
Fragment 6
Fragment 7
(B)
vORF Vector
attL1
vORF1
STOP
EcoRI
BsmBI
vORF2
HindIII
vORF3
attL2
1 Isolation of vORF1
EcoRI Digestion & Ligation
HindIII Negative Selection
First vORF Ligation Product:
2 Separation of Additional vORFs
BsmBI and HindIII Digestion
Ligation into BseRI digested vector
STOP Cassette Insertion
BamHI
SpeI
Final vORF Ligation Product:
(C)
Cluster 2: Mononegavirales
RdRp Catalytic Domain vORFs
69 members
Filoviridae
Paramyxoviridae
Pneumoviridae
Cluster 83: dsDNA Virus ribonucleoside-diphosphate reductase (small subunit)
19 members
Asfarviridae
Herpesviridae
Poxviridae
(D)
ori
T7 Promoter
attL1
pFuji101
3,616 bp
(excluding synthesized fragment)
Synthesized Fragment
KanR
TolA
attL2
rrnB Terminators
I-SceI
SfiI
I-CeuI
PI-SceI
vORF
STOP
BC
TolA
T7 Promoter
Kozak
Shine-Dalgarno
Synthesized Fragment
Stop > No Stop Cassette
SbfI
BseRI
BamHI
EcoRI
(E)
Generalized vORF Barcode Design:
Unique 18 bp Barcode
Diversified Region (10 bp)
XXXXXXXXXXXXXXXXXXCCTGCAGGXXXXXXXXXXAGCGAATTCA
SbfI
EcoRI
(F)
Barcode Diversification Strategy:
Starting BC1 Barcode:
BC1
XXXXXXXXXXXXXXXXXXCCTGCAGGXXXXXXXXXXAGCGAATTCA
XXXXXXXXXXXXXXXXXXGGACGTCCXXXXXXXXXXTCGCTTAAGT
SbfI and EcoRI Digestion:
XXXXXXXXXXXXXXXXXXCCTGCA GGXXXXXXXXXXAGCG AATTCA
XXXXXXXXXXXXXXXXXXGG ACGTCCXXXXXXXXXXTCGCTTAA GT
Annealing and Ligating BC2-5 Inserts:
BC2-5
GGXXXXXXXXXXAGCG
ACGTCCXXXXXXXXXXTCGCTTAA
Diversified BC2-5 Barcodes:
BC2-5
XXXXXXXXXXXXXXXXXXCCTGCAGGXXXXXXXXXXAGCGAATTCA
XXXXXXXXXXXXXXXXXXGGACGTCCXXXXXXXXXXTCGCTTAAGT
(G)
Input 2 Barcode Read Counts
Input 1 Barcode Read Counts
r² = 0.891

Figure S1. Development of a viral ORFeome. ***Related to Figure 1.***
(A) A schematic of the strategy for concatenating small vORFs or tiling large (> 570 aa) vORFs into constructs for synthesis. Portions of this panel were generated with BioRender.
(B) A schematic representation of the cloning strategy to generate single vORF vectors from concatenated vORFs. Portions of this panel were generated with BioRender.
(C) Representative depiction of the structure of two vORF clusters containing vORFs from multiple viral families. Portions of this panel were generated with BioRender.
(D) A schematic of the design of pFuji101, the vector used for the viral ORFeome, with insets showing the features downstream of the rrnB terminators included in the design and the design of the synthesized segment of the vector that is unique to each vORF. Portions of this panel were generated with BioRender.
(E) A schematic depiction of the structure of the barcode region used for the viral ORFeome collection, consisting of a core barcode unique to each vORF, *Eco*RI and *Sbf*I restriction sites, and a diversifiable region to generate additional unique barcodes for each vORF.
(F) A schematic representation of the cloning strategy used to generate diversified barcodes.
(G) A depiction of the total read counts associated with each barcode detected following barcode amplification from genomic DNA from two populations of A375 cells transduced with the viral ORFeome following 48 h of doxycycline induction.

Figure S2

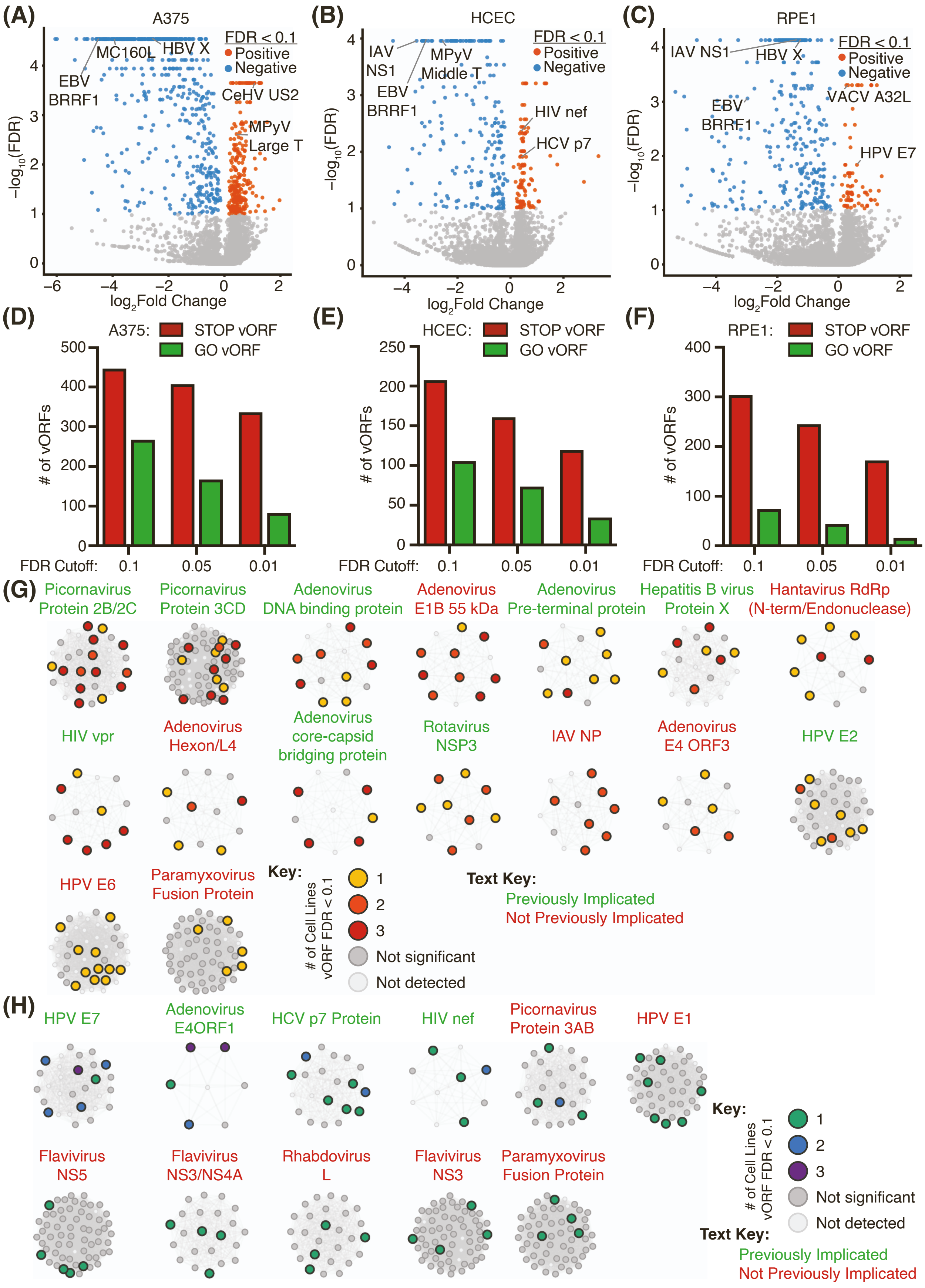

(A) A375
FDR < 0.1
Positive
Negative
MC160L
HBV X
EBV BRRF1
CeHV US2
MPyV Large T
$-\log_{10}$(FDR)
$\log_2$Fold Change
(B) HCEC
IAV NS1
MPyV Middle T
EBV BRRF1
HIV nef
HCV p7
(C) RPE1
IAV NS1
HBV X
EBV BRRF1
VACV A32L
HPV E7
(D) A375: STOP vORF GO vORF
(E) HCEC: STOP vORF GO vORF
(F) RPE1: STOP vORF GO vORF
# of vORFs
FDR Cutoff: 0.1 0.05 0.01
(G)
Picornavirus Protein 2B/2C
Picornavirus Protein 3CD
Adenovirus DNA binding protein
Adenovirus E1B 55 kDa
Adenovirus Pre-terminal protein
Hepatitis B virus Protein X
Hantavirus RdRp (N-term/Endonuclease)
HIV vpr
Adenovirus Hexon/L4
Adenovirus core-capsid bridging protein
Rotavirus NSP3
IAV NP
Adenovirus E4 ORF3
HPV E2
HPV E6
Paramyxovirus Fusion Protein
Key:
# of Cell Lines vORF FDR < 0.1
1
2
3
Not significant
Not detected
Text Key:
Previously Implicated
Not Previously Implicated
(H)
HPV E7
Adenovirus E4ORF1
HCV p7 Protein
HIV nef
Picornavirus Protein 3AB
HPV E1
Flavivirus NS5
Flavivirus NS3/NS4A
Rhabdovirus L
Flavivirus NS3
Paramyxovirus Fusion Protein
Key:
# of Cell Lines vORF FDR < 0.1
1
2
3
Not significant
Not detected
Text Key:
Previously Implicated
Not Previously Implicated

Figure S2. Identification of vORFs that regulate cellular proliferation. ***Related to Figure 2.***
(A-C) Volcano plots of the viral ORFeome proliferation screens in **(A)** A375, **(B)** HCEC, and **(C)** RPE1 cells with representative vORFs noted. Log_2fold changes and FDRs were calculated by comparing the 7 population doubling samples with an input sample from the initiation of vORF expression with doxycycline.
(D-F) The number of vORFs achieving significance at the indicated FDR cutoff values in the **(D)** A375, **(E)** HCEC, and **(F)** RPE1 proliferation screens.
(G) Clusters of ≥ 5 vORFs with greater than 25% sequence homology that reduced cellular proliferation in at least one cell line (negative FDR < 0.1) are depicted. vORF clusters indicated in green lettering are known inhibitors of cellular proliferation or survival, whilst those in red lettering are not.
(H) Clusters of ≥ 3 vORFs with greater than 25% sequence homology that increased cellular proliferation in at least one cell line (positive FDR < 0.1) are depicted. vORF clusters indicated in green lettering are known to increase cellular proliferation or survival, whilst those in red lettering are not.

Figure S3

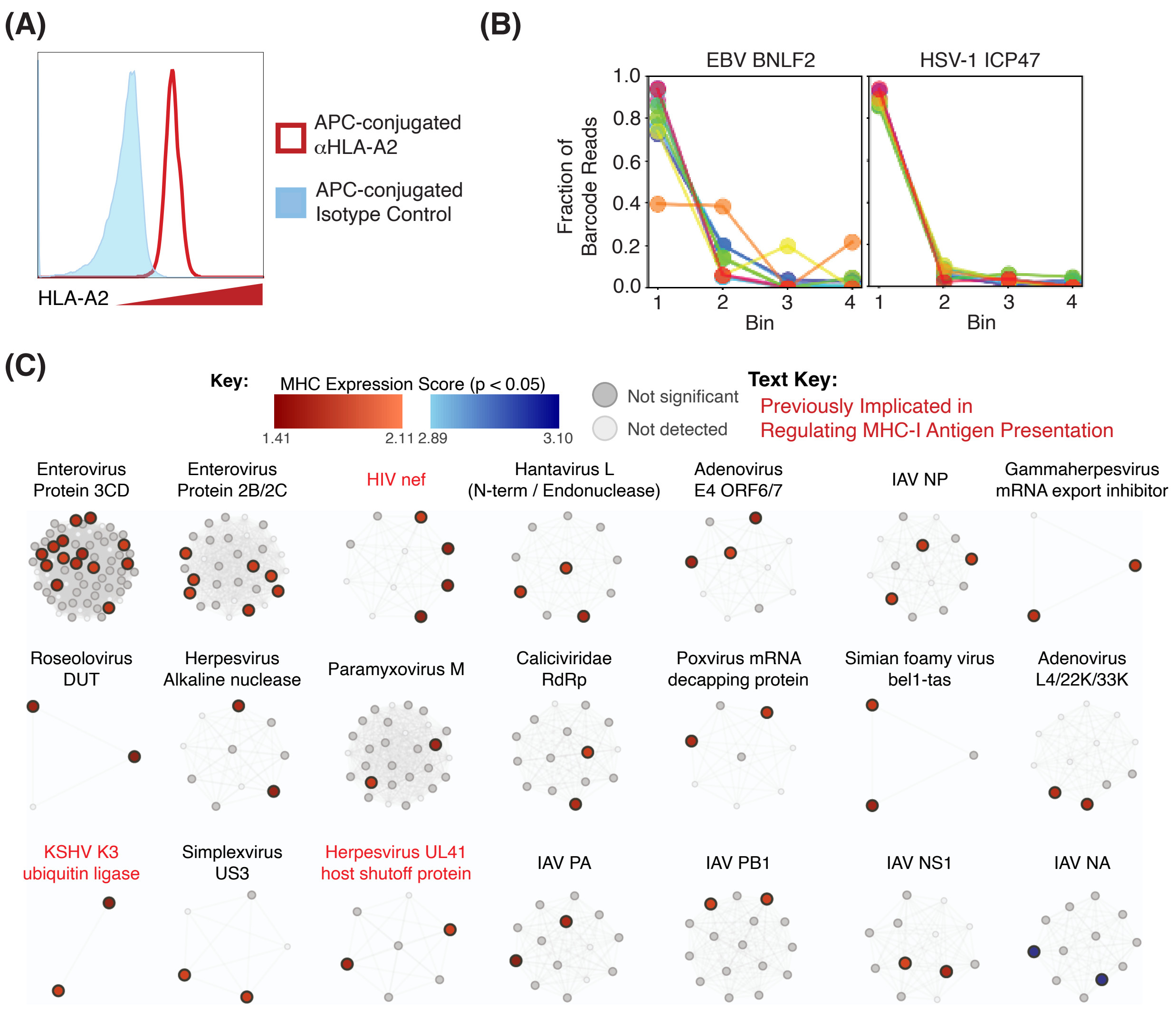
(A)
APC-conjugated αHLA-A2
APC-conjugated Isotype Control
HLA-A2
(B)
EBV BNLF2
HSV-1 ICP47
Fraction of Barcode Reads
1.0
0.8
0.6
0.4
0.2
0.0
1
2
3
4
Bin
(C)
Key:
MHC Expression Score (p < 0.05)
1.41
2.11
2.89
3.10
Not significant
Not detected
Text Key:
Previously Implicated in Regulating MHC-I Antigen Presentation
Enterovirus Protein 3CD
Enterovirus Protein 2B/2C
HIV nef
Hantavirus L (N-term / Endonuclease)
Adenovirus E4 ORF6/7
IAV NP
Gammaherpesvirus mRNA export inhibitor
Roseolovirus DUT
Herpesvirus Alkaline nuclease
Paramyxovirus M
Caliciviridae RdRp
Poxvirus mRNA decapping protein
Simian foamy virus bel1-tas
Adenovirus L4/22K/33K
KSHV K3 ubiquitin ligase
Simplexvirus US3
Herpesvirus UL41 host shutoff protein
IAV PA
IAV PB1
IAV NS1
IAV NA

Figure S3. A genetic screen identifies vORFs that regulate MHC-I antigen presentation. ***Related to Figure 3.***

(A) Flow cytometry analysis of HLA-A2 staining on A375 cells.

(B) Representation of the fraction of individual barcode reads detected in each bin for two known regulators of MHC-I expression that achieved significance in the screen. Colors represent different barcodes.

(C) Several homologous vORFs achieved significance as antagonists of HLA-A2 surface staining in A375 cells. Clusters containing at least 2 vORFs with greater than 25% sequence identity that significantly reduced HLA-A2 surface staining ($p < 0.05$) identified by mmseq2 are depicted. Clusters containing vORFs previously characterized to inhibit MHC-I expression are indicated in green, while clusters containing vORFs not previously characterized to inhibit MHC-I expression are indicated in red.

Figure S4

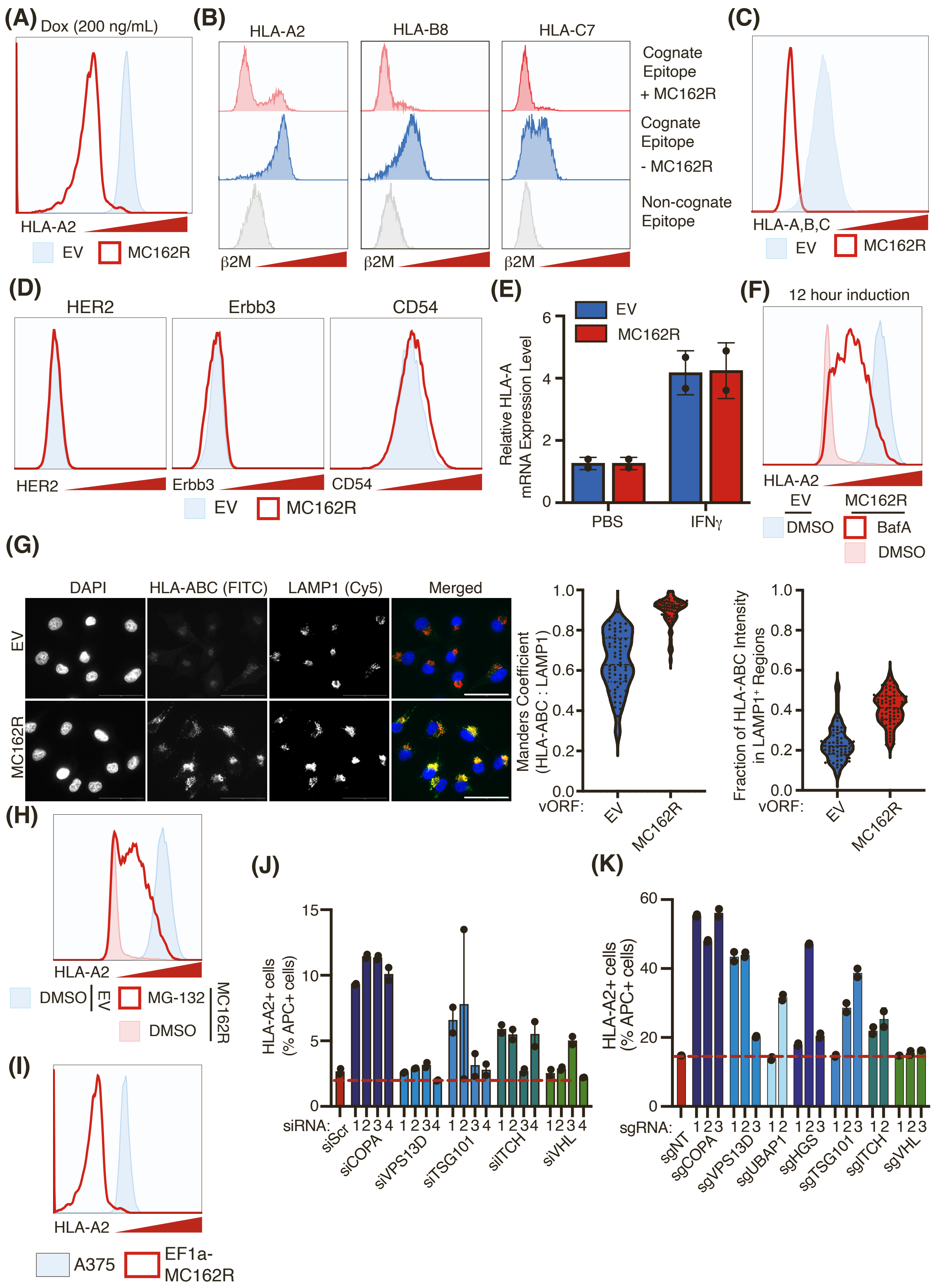

(A) Dox (200 ng/mL)
HLA-A2
EV
MC162R
(B)
HLA-A2
HLA-B8
HLA-C7
Cognate Epitope + MC162R
Cognate Epitope - MC162R
Non-cognate Epitope
β2M
(C)
HLA-A,B,C
EV
MC162R
(D)
HER2
Erbb3
CD54
EV
MC162R
(E)
EV
MC162R
Relative HLA-A mRNA Expression Level
PBS
IFNγ
(F) 12 hour induction
HLA-A2
EV
MC162R
DMSO
BafA
DMSO
(G)
DAPI
HLA-ABC (FITC)
LAMP1 (Cy5)
Merged
EV
MC162R
Manders Coefficient (HLA-ABC : LAMP1)
vORF:
EV
MC162R
Fraction of HLA-ABC Intensity in LAMP1+ Regions
vORF:
EV
MC162R
(H)
HLA-A2
DMSO
EV
MG-132
DMSO
MC162R
(I)
HLA-A2
A375
EF1a-MC162R
(J)
HLA-A2+ cells (% APC+ cells)
siRNA:
siScr
siCOPA
siVPS13D
siTSG101
siITCH
siVHL
(K)
HLA-A2+ cells (% APC+ cells)
sgRNA:
sgNT
sgCOPA
sgVPS13D
sgUBAP1
sgHGS
sgTSG101
sgITCH
sgVHL

Figure S4. MC162R antagonizes MHC-I antigen presentation via MHC-I lysosomal degradation. ***Related to Figure 4.***

(A) Expression of HLA-A2 in A375 cells expressing doxycycline-inducible Flag- MC162R or an empty vector in the presence of doxycycline as determined by flow cytometry.

(B) Expression of the indicated HA-tagged HLA constructs as determined by flow cytometry in HEK-293T MHC-I-/- TAP-/- cells expressing MC162R or empty vector in the presence or absence of their cognate epitope.

(C) Pan-HLA-ABC cell surface staining in A375 cells expressing doxycycline-inducible FlagMC162R or an empty vector control in the presence of doxycycline as determined by flow cytometry.

(D) Expression of HER2, Erbb3, or CD54 in A375 cells expressing doxycycline-inducible FlagMC162R or an empty vector control in the presence of doxycycline as determined by flow cytometry.

(E) Quantitative reverse transcription PCR for HLA-A mRNA transcripts in A375 cells expressing doxycycline-inducible FlagMC162R or an empty vector control. Data are representative of n = 2 biological replicates per condition and are depicted as the mean +/- standard deviation.

(F) Expression of cell surface HLA-A2 in A375 cells expressing doxycycline-inducible Flag- tagged MC162R or an empty vector control following treatment with bafilomycin A.

(G) (Left) Fluorescence microscopy for co-localization of HLA-ABC and late endosome/early lysosome marker LAMP1 in A375 cells expressing doxycycline-induced FlagMC162R (n = 67 cells) or an empty vector control (n = 65 cells). All cells were treated with bafilomycin A for 12 h prior to fixation to allow for observation of HLA-A localization. Scale bar, 50 μm. (Right) Quantification of the colocalization between MC162R (FLAG) and HLA-ABC.

(H) Expression of cell surface HLA-A2 in A375 cells expressing doxycycline-inducible FlagMC162R or an empty vector control following treatment with MG132.

(I) Expression of cell surface HLA-A2 in A375 cells expressing a constitutively expressed FlagMC162R compared to the parental A375 cells.

(J) Quantification of HLA-A2 surface staining in A375 cells expressing MC162R and the indicated siRNAs. Data are replicates assayed in duplicate and depicted as mean ± range with individual values plotted. Data are representative of multiple independent experiments.

(K) Quantification of HLA-A2 surface staining in A375 cells expressing MC162R and the indicated sgRNAs. Data are replicates assayed in duplicate and depicted as mean ± range with individual values plotted. Data are representative of multiple independent experiments.

Figure S5

(A)

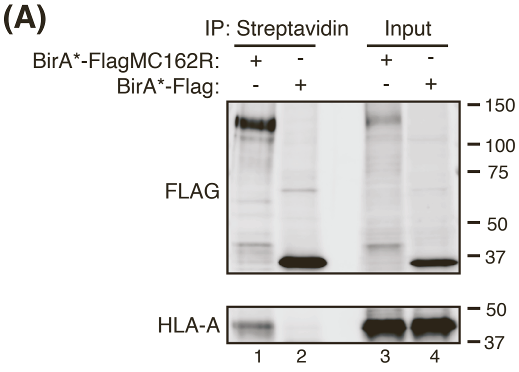

(B)

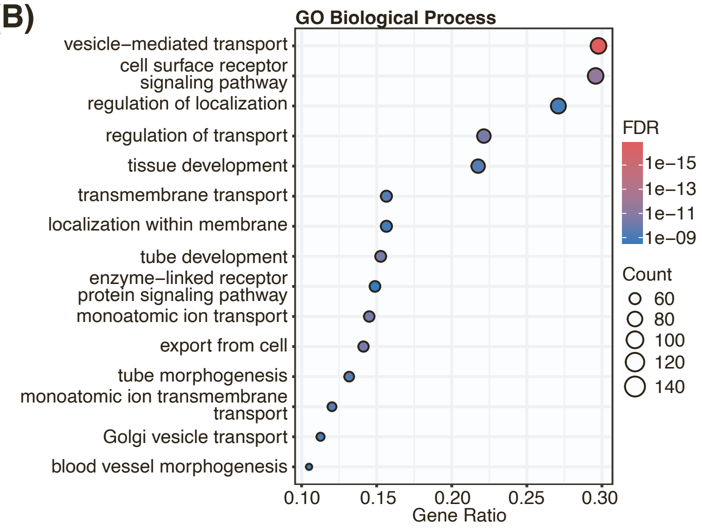

(C)

Gene Symbol	Log2FC	p–value	FDR
ITCH	6.09	6.25e–05	0.000386
NEDD4L	4.23	5.64e–05	0.000606
PJA2	4.19	1.85e–06	0.000090
NEDD4	3.96	2.45e–07	0.000022
WWP2	4.17	8.82e–06	0.000190
TRIM41	3.44	3.31e–04	0.001894
TRIM13	3.24	1.49e–04	0.001096
UBR2	3.16	7.28e–05	0.000695
WWP1	2.05	9.26e–03	0.002260
TRIM32	1.58	0.0196	0.038616

(D)

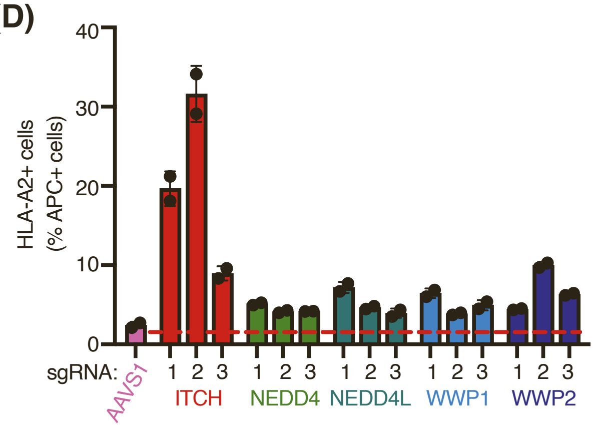

(E)

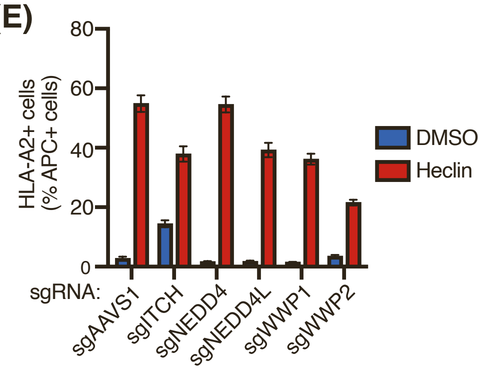

(F)

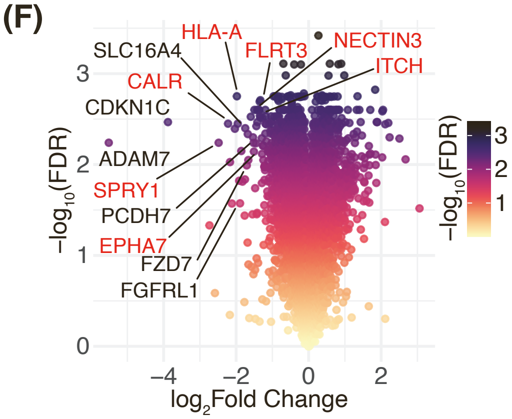

(G)

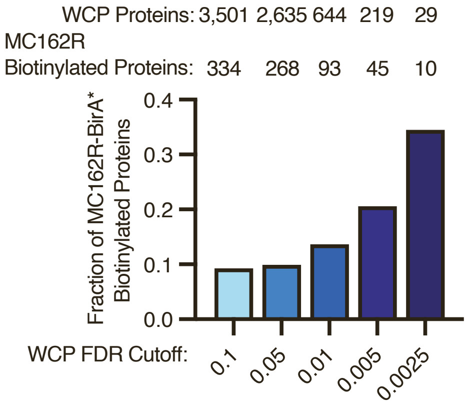

Figure S5. MC162R exploits multiple E3 ligases to degrade MHC-I. ***Related to Figure 5.***

(A) Immunoblot for Flag and HLA-A following streptavidin pulldown in A375 cells expressing either BirA*-Flag or BirA*-FlagMC162R demonstrating that BirA*-FlagMC162R results in HLA-A biotinylation and biotinylation of BirA*-MC162R and BirA*-Flag constructs.

(B) Enrichment analysis of gene sets (Gene Ontology) for proteins that were enriched in the BirA*-FlagMC162R sample. The 20 gene sets with the highest enrichment scores are shown, ranked by FDR.

(C) The top 10 ubiquitin E3 ligases enriched following streptavidin pulldown in BirA*-MC162R expressing cells, sorted by fold change in spectral counts. NEDD4-like ubiquitin E3 ligases are highlighted in red.

(D) Quantification of the fraction of HLA-A2+ cells detected by flow cytometry for the indicated sgRNA targeting a NEDD4-like E3 ligase. Data are replicates assayed in duplicate and depicted as mean ± range with individual values plotted. Data are representative of multiple independent experiments.

(E) HLA-A2 expression as detected by flow cytometry in A375 cells harboring the indicated sgRNA had MC162R expression induced for 12 h in the presence or absence of heclin. Data are technical replicates assayed in triplicate and depicted as mean ± SD. Data are representative of multiple independent experiments.

(F) Changes in protein abundance detected via whole cell proteomics in A375 cells expressing MC162R compared to wild type A375 cells. Selected proteins are labelled, with red text identifying proteins identified by MC162R proximity labeling.

(G) The fraction of proteins identified by MC162R proximity labeling (FDR < 0.1 in that assay) identified in the subset of proteins with decreased abundance identified by whole cell proteomics in MC162R expressing cells at the indicated FDR cutoffs. The total number of proteins identified by whole cell proteomics and the number of proteins identified in MC162R proximity labeling assay at each whole cell proteomics FDR cutoff are indicated above each bar.

Figure S6

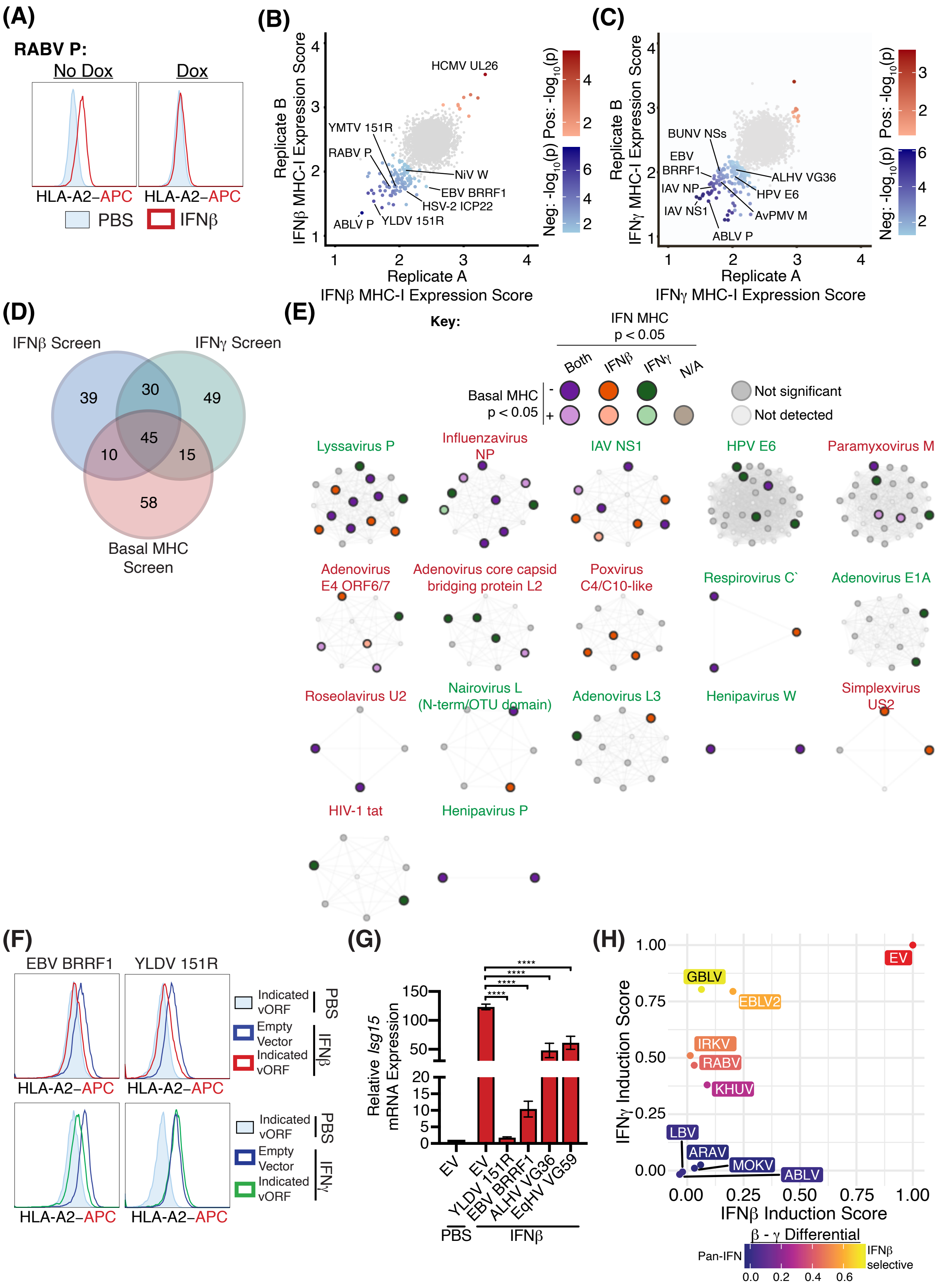

(A)
RABV P:
No Dox
Dox
HLA-A2–APC
HLA-A2–APC
PBS
IFNβ
(B)
HCMV UL26
YMTV 151R
RABV P
NiV W
EBV BRRF1
HSV-2 ICP22
ABLV P
YLDV 151R
Replicate B
IFNβ MHC-I Expression Score
Replicate A
IFNβ MHC-I Expression Score
Pos: $-\log_{10}(p)$
Neg: $-\log_{10}(p)$
(C)
BUNV NSs
EBV BRRF1
IAV NP
IAV NS1
ALHV VG36
HPV E6
AvPMV M
ABLV P
Replicate B
IFNγ MHC-I Expression Score
Replicate A
IFNγ MHC-I Expression Score
Pos: $-\log_{10}(p)$
Neg: $-\log_{10}(p)$
(D)
IFNβ Screen
IFNγ Screen
39
30
49
45
10
15
58
Basal MHC Screen
(E)
Key:
IFN MHC $p < 0.05$
Both
IFNβ
IFNγ
N/A
Basal MHC $p < 0.05$
-
+
Not significant
Not detected
Lyssavirus P
Influenzavirus NP
IAV NS1
HPV E6
Paramyxovirus M
Adenovirus E4 ORF6/7
Adenovirus core capsid bridging protein L2
Poxvirus C4/C10-like
Respirovirus C`
Adenovirus E1A
Roseolavirus U2
Nairovirus L (N-term/OTU domain)
Adenovirus L3
Henipavirus W
Simplexvirus US2
HIV-1 tat
Henipavirus P
(F)
EBV BRRF1
YLDV 151R
HLA-A2–APC
Indicated vORF
PBS
Empty Vector
Indicated vORF
IFNβ
Indicated vORF
PBS
Empty Vector
Indicated vORF
IFNγ
(G)
Relative *Isg15* mRNA Expression
****
EV
EV
YLDV 151R
EBV BRRF1
ALHV VG36
EqHV VG59
PBS
IFNβ
(H)
EV
GBLV
EBLV2
IRKV
RABV
KHUV
LBV
ARAV
MOKV
ABLV
IFNγ Induction Score
IFNβ Induction Score
β - γ Differential
Pan-IFN
IFNβ selective

Figure S6. Identification of viral ORFs that regulate interferon signaling. ***Related to Figure 6.***

(A) A tetracycline-inducible expression vector for RABV P was independently transduced into A375 cells and assessed for its ability to restrict IFNβ-mediated increases in surface HLA-A2 by flow cytometry following staining with an APC-conjugated HLA-A2 primary antibody.

(B) The IFNβ-induced HLA-A2 expression scores for all vORFs identified across two replicates.

(C) The IFNγ-induced HLA-A2 expression scores for all vORFs identified across two replicates.

(D) A Venn diagram representation of the vORFs that achieved significance in one or more of the basal MHC expression screen, the IFNβ-induced MHC expression screen, or the IFNγ -induced MHC expression screen. The number of vORFs that achieved significance in each overlapping area is indicated.

(E) Identification of clusters of related vORFs that regulate IFN signaling. vORF clusters with > 25% sequence identity (determined by mmseq2) and containing at least 2 vORFs that significantly reduced IFNβ or IFNγ-induced HLA-A2 cell surface expression, without impairing basal HLA-A2 cell surface expression, are depicted. vORFs that reduced basal HLA-A2 cell surface expression within these clusters are further annotated. Clusters containing vORFs previously characterized to inhibit IFN signaling are indicated in green lettering, while clusters containing vORFs not previously characterized to inhibit IFN signaling are indicated in red lettering.

(F) Histogram depiction of HLA-A2 cell surface staining using an APC-conjugated primary antibody in A375 cells expressing the indicated viral ORF following stimulation with IFNβ or IFNγ.

(G) Quantitative reverse transcription PCR for *ISG15* mRNA expression following IFNβ stimulus in A375 cells expressing an empty vector, YLDV 151R, EBV BRRF1, ALHV-1 VG36, or EqHV VG59 (n = 3 biological replicates). Statistical comparisons were performed with a two-way ANOVA with pairwise comparisons via Tukey's multiple comparisons test, with significant results between IFN treated samples noted: **** *adj p* < 0.0001

(H) Validation of lyssavirus P vORFs as selective regulators of IFNγ signaling. Changes in HLA-A2 staining were determined by taking the $\log_2$ of the MFIs between cells treated with IFN/cells treated with PBS and normalized to the empty vector control.

Figure S7

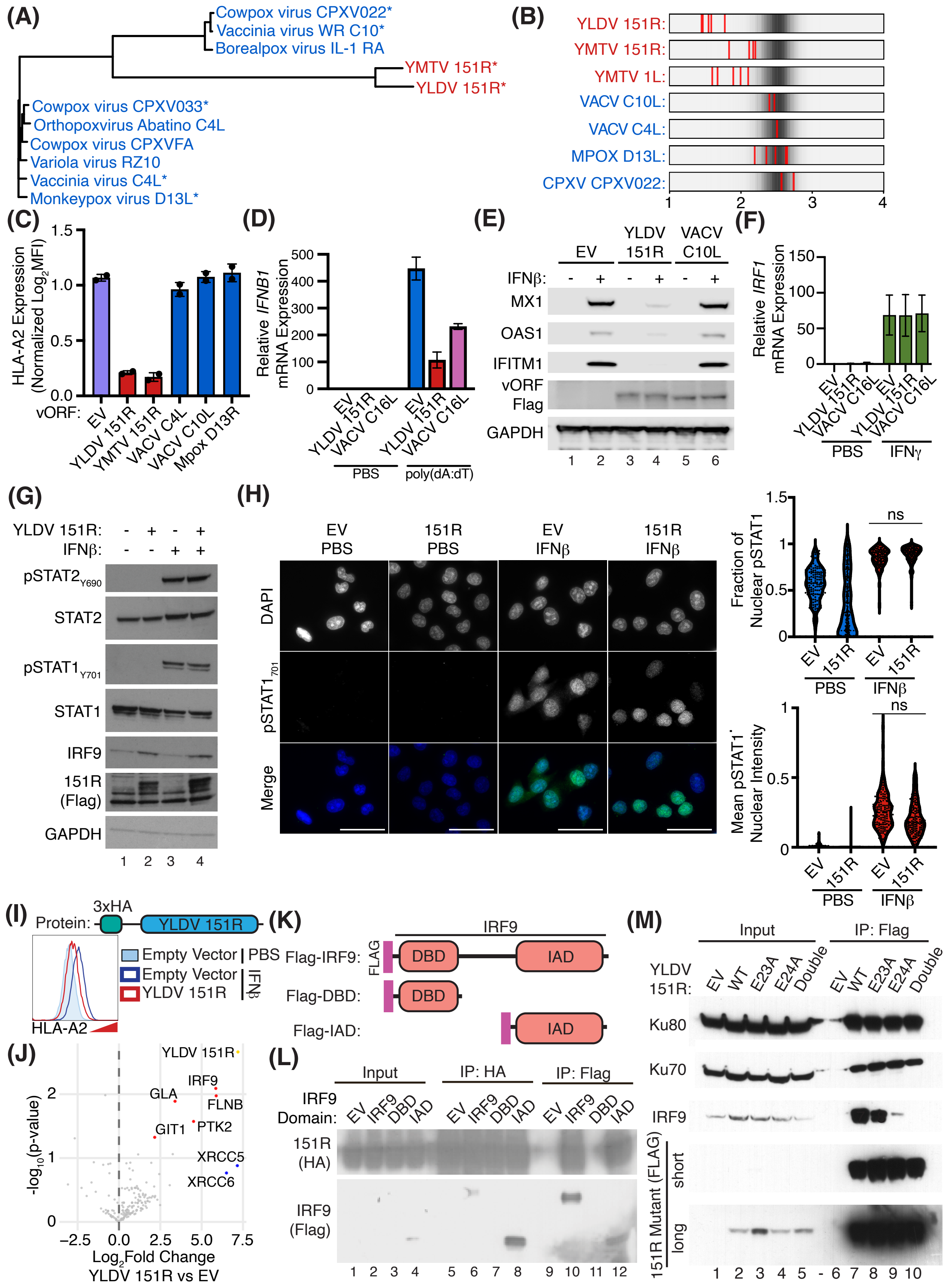

(A)
Cowpox virus CPXV022*
Vaccinia virus WR C10*
Borealpox virus IL-1 RA
YMTV 151R*
YLDV 151R*
Cowpox virus CPXV033*
Orthopoxvirus Abatino C4L
Cowpox virus CPXVFA
Variola virus RZ10
Vaccinia virus C4L*
Monkeypox virus D13L*
(B)
YLDV 151R:
YMTV 151R:
YMTV 1L:
VACV C10L:
VACV C4L:
MPOX D13L:
CPXV CPXV022:
(C)
HLA-A2 Expression (Normalized Log2MFI)
vORF:
EV
YLDV 151R
YMTV 151R
VACV C4L
VACV C10L
Mpox D13R
(D)
Relative IFNB1 mRNA Expression
PBS
poly(dA:dT)
(E)
EV
YLDV 151R
VACV C10L
IFNβ:
MX1
OAS1
IFITM1
vORF Flag
GAPDH
(F)
Relative IRF1 mRNA Expression
PBS
IFNγ
(G)
YLDV 151R:
IFNβ:
pSTAT2 Y690
STAT2
pSTAT1 Y701
STAT1
IRF9
151R (Flag)
GAPDH
(H)
EV PBS
151R PBS
EV IFNβ
151R IFNβ
DAPI
pSTAT1 701
Merge
Fraction of Nuclear pSTAT1
Mean pSTAT1 Nuclear Intensity
ns
(I)
3xHA
Protein:
YLDV 151R
Empty Vector
Empty Vector
YLDV 151R
PBS
IFNβ
HLA-A2
(J)
YLDV 151R
IRF9
GLA
FLNB
GIT1
PTK2
XRCC5
XRCC6
-log10(p-value)
Log2Fold Change YLDV 151R vs EV
(K)
IRF9
Flag-IRF9:
Flag-DBD:
Flag-IAD:
FLAG
DBD
IAD
(L)
Input
IP: HA
IP: Flag
IRF9 Domain:
EV
IRF9
DBD
IAD
151R (HA)
IRF9 (Flag)
(M)
Input
IP: Flag
YLDV 151R:
EV
WT
E23A
E24A
Double
Ku80
Ku70
IRF9
151R Mutant (FLAG)
short
long

Figure S7. Yatapoxvirus 151R vORFs specifically regulate IFNβ signaling via an interaction with IRF9. ***Related to Figure 7.***

(A) A phylogenetic tree of members of the poxvirus C4/C10 protein family identified following three rounds of PSI-BLAST on YLDV 151R and collapsed on 98% homology. vORFs present in the viral ORFeome are indicated by an asterisk. Orthopoxvirus proteins are indicated in blue, while yatapoxvirus ORFs are indicated in red.

(B) Barcode distribution plots of the performance of C4/C10 family vORFs that are present in the viral ORFeome in the IFNβ screen.

(C) Quantification of the change in HLA-A2 staining following IFNβ treatment in A375 cells expressing the indicated vORF and normalized to EV. Changes in HLA-A2 staining were determined by taking the $\log_2$ of the MFIs between cells treated with IFNβ/cells treated with PBS. Data are representative of n = 2 biological replicates per condition and are depicted as the mean ± standard deviation.

(D) Quantitative reverse transcription PCR for *IFNB1* mRNA expression following poly(dA:dT) transfection in A375 cells expressing an empty vector, YLDV 151R, or VACV WR C16L (n = 3 biological replicates). Data are represented as mean ± SD.

(E) Immunoblot for IFITM1, OAS1, MX1, FLAG, and GAPDH from A375 cells expressing an empty vector control, Flag-tagged YLDV 151R, or Flag-tagged VACV C10L and stimulated with IFNβ for 16 h.

(F) Quantitative reverse transcription PCR for *IRF1* mRNA expression following IFNγ treatment in A375 cells expressing an empty vector, YLDV 151R, or VACV WR C16L (n = 3 biological replicates). Data are represented as mean ± SD.

(G) Immunoblot for pSTAT1, STAT1, pSTAT2, STAT2, IRF9, and Flag-YLDV 151R from A375 cells expressing an empty vector control or Flag-YLDV 151R and stimulated with IFNβ for 30 min.

(H) Fluorescence microscopy imaging of A375 cells expressing Flag-YLDV 151R or an empty vector control in the presence (YLDV 151R expressing, n = 157 cells; EV, n = 143 cells) or absence (YLDV 151R expressing, n = 183 cells; EV, n = 148 cells) of IFNβ stimulus and stained with an anti-pSTAT1Y701 primary antibody and FITC-conjugated secondary antibody and mounted with DAPI. Scale bar, 50 μm. (Right) Quantification of the ratio of nuclear pSTAT1 to total pSTAT1 observed in the nuclear and perinuclear regions (top) or mean nuclear pSTAT1 signal intensity (bottom).

(I) (top) Design of an expression vector for a 3xHA-tagged YLDV 151R. (bottom) Histogram depiction of HLA-A2 cell surface staining using an APC-conjugated primary antibody in A375 cells expressing 3xHA-YLDV 151R following stimulation with IFNβ.

(J) Enrichment of proteins identified via immunoprecipitation and mass spectrometry following anti-HA immunoprecipitation in 3xHA-YLDV 151R expressing A375 cells relative to A375 cells expressing a 3xHA vector alone. Significantly enriched proteins are indicated in red, while proteins with a non-significant p-value but a $\log_2$fold change > 5 are indicated in blue.

(K) Schematic of truncation variants corresponding to the DBD and IAD of IRF9.

(L) Immunoblot for 3xHA-tagged YLDV 151R and Flag-tagged IRF9 truncation variants following immunoprecipitation of Flag-tagged full length IRF9, IRF9 DBD, and IRF9 IAD or 3xHA-YLDV 151R from A375 cells.

(M) Immunoblot for IRF9, Ku70, Ku80, and Flag-tagged YLDV 151R mutants following immunoprecipitation of Flag-tagged full length YLDV $151R_{WT}$, YLDV $151R_{E23A}$, YLDV $151R_{E24A}$, and YLDV $151R_{E23A\text{-}E24A}$ from A375 cells as indicated.

Statistical comparisons were performed with (F) a two-way ANOVA with pairwise comparisons via Tukey's multiple comparisons test, with significant results between IFN treated samples noted: **** *adj p* < 0.0001, ** *adj p* < 0.01, * *adj p* < 0.05, (H) a Kruskal-Wallis test with pairwise comparisons via Dunn's multiple comparisons test.
